# Single-cell RNA sequencing reveals that Danggui Buxue Tang decoction facilitates wound healing after anal fistula by promoting M2 macrophage polarization

**DOI:** 10.1186/s41065-025-00578-2

**Published:** 2025-10-09

**Authors:** Xue Pang, Yutao Wang, Jianzhuang Guo

**Affiliations:** 1 Department of Proctology, The First Affiliated Hospital of Shandong First Medical University & Shandong Province Qianfoshan Hospital, 16766 Jingshi Road, Jinan, 250014 Shandong China; 2 Department of Peripheral Vascular Disease, Guang’anmen Hospital Jinan Hospital, China Academy of Chinese Medical Sciences (Jinan Municipal Hospital of Traditional Chinese Medicine), Jinan, 250012 Shandong China; 3 Department of Clinical Laboratory, The First Affiliated Hospital of Shandong First Medical University & Shandong Province Qianfoshan Hospital, Jinan, 250014 Shandong China

**Keywords:** Danggui buxue tang, Macrophages, Anal fistula, Single cell RNA sequencing, Anti-inflammation

## Abstract

**Background:**

We aim to employ single-cell RNA (scRNA) sequencing technology to investigate potential regulatory mechanism of Danggui Buxue Tang (DBT) in wound healing for its utilization in post-anal fistula surgery recovery.

**Methods:**

Fistula-like wound model in mice was established and administered DBT to assess its effects. Mice were divided into control and DBT groups and collected samples on the first day and 7th day after model establishment. The DBT was prepared from *Astragalus membranaceus* and *Angelica sinensis*. ScRNA sequencing was performed on each group.

**Results:**

Our results showed that DBT treatment obviously reduced wound area in mice with anal fistula through activation of OPN/PI3K/Akt/eNOS signaling. Furthermore, the results of scRNA sequencing showed that all cells were clustered into 7 types, and the macrophages were categorized into 13 distinct clusters. In the early stages of wound formation, M1-like macrophages (M1C1) abundant in both groups at day1. However, by day 7 post-injury, the DBT-treated group exhibited a reduction in the infiltration of M1-like macrophages (M1C1) compared to the model group. Conversely, the proportion of M2-like macrophages (M2C3) showed a marked increase in the DBT group at day 7, while decreasing in the model group. Pseudo-time trajectory analysis confirmed that DBT treatment modulates macrophage polarization, potentially enhancing the wound healing process by promoting a transition from pro-inflammatory to anti-inflammatory macrophage populations.

**Conclusion:**

DBT has the potential to accelerate wound healing after anal fistula by promoting M2 macrophage polarization, likely through activation of the PI3K/Akt signaling pathway.

**Supplementary Information:**

The online version contains supplementary material available at 10.1186/s41065-025-00578-2.

## Introduction

Anal fistula is a medical condition characterized by an abnormal connection or tunnel between the anal canal and the skin near the anus [1]. This condition can lead to recurrent infections, abscess formation, and persistent discharge, causing significant discomfort and distress for affected individuals [2]. The management of anal fistulas often involves surgical intervention aimed at removing the fistula tract to promote healing and prevent recurrence [3]. However, postoperative wound care and recovery are critical aspects of the treatment process that require meticulous attention even if the trauma area is small [4]. Wound healing after fistula surgery can be a complex and prolonged process, influenced by various factors such as the size and location of the fistula, the individual’s overall health, and the presence of any underlying conditions like diabetes [5]. The healing process typically involves initial phase, inflammatory phase, proliferative phase, and maturation or remodeling phase [6]. Macrophages function vitally in all phases of wound healing [7].

Macrophages are a type of white blood cell that plays a crucial role in the immune system, acting as the body’s first line of defense against pathogens [8]. These cells exhibit remarkable versatility, capable of assuming various activation states, notably recognized as the M1 and M2 phenotypes [9]. M1 macrophages, or “classically activated” macrophages, are characterized by their pro-inflammatory role. They are typically induced by Th1 cytokines such as interferon-γ (IFN-γ) and lipopolysaccharide (LPS) [10]. M1 macrophages are efficient in eliminating intracellular pathogens through the production of reactive oxygen species (ROS) and reactive nitrogen species (RNS) [11]. They also secrete pro-inflammatory cytokines, which help recruit other immune cells to the site of infection and enhance antigen presentation to activate T cells [12]. On the other hand, M2 macrophages, or “alternatively activated” macrophages, play a role in anti-inflammatory responses and tissue repair [13, 14]. They are induced by Th2 cytokines such as interleukin-4 (IL-4) and interleukin-13 (IL-13) [15]. M2 macrophages secrete anti-inflammatory cytokines, promote wound healing, and contribute to the resolution of inflammation [16].

Danggui Buxue Tang (DBT) is a traditional Chinese herbal decoction that has been cherished for its restorative powers. The primary ingredients of this formula are the roots of *Astragalus membranaceus* (Huangqi), known for its immune-modulating and tonic effects, and the roots of *Angelica sinensis* (Danggui), which is lauded for its blood-nourishing properties [17]. This blend is effective in invigorating the blood and supporting the body’s natural healing processes, including the regeneration of tissues and the closure of wounds. The formula’s ability to enhance the body’s qi and blood, along with its wound-healing capabilities, underscores its importance in maintaining overall health and well-being [18]. According to our previous unpublished research, DBT has demonstrated efficacy in treating infectious wounds, a testament to its potent anti-inflammatory and reparative actions. By reducing inflammation and promoting skin integrity, this herbal decoction aids in the swift recovery from skin injuries and conditions.

In this study, we aim to employ single-cell RNA (scRNA) sequencing technology to investigate the regulatory effects of DBT on the polarization of macrophages in wound tissues. By analyzing the classification and gene expression of macrophages within the wound, this research intends to preliminarily elucidate the mechanism of action of DBT, thereby providing evidence for the role of DBT and promoting its utilization in wound healing post-anal fistula surgery.

## Materials and methods

### Preparation of Danggui Buxue Tang

The traditional Chinese medicinal herbs *Astragalus membranaceus* roots (Huangqi, no. 240402) and *Angelica sinensis* roots (Danggui, no. 240701) were purchased from the herbal pharmacy of Jinan city hospital of Traditional Chinese Medicine and were identified by Professor Zhang Hongxing, a nationally renowned expert in traditional Chinese medicine and a mentor in the sixth batch of national traditional Chinese medicine experts’ academic experience guidance program. *Astragalus membranaceus* and *Angelica sinensis* were prepared in a 6:1 weight ratio and decocted with water [19] to obtain the DBT decoction.

### Animal study

Ethics Statement: All animal experiments were approved by the Experimental animal Ethics Review Committee of the First Affiliated Hospital of Shandong First Medical University (No. S545 (2021)). All possible measures were taken to guarantee the well-being, attention, and compassionate care of the animals, as well as to reduce their suffering to a minimum during the entire course of the research.

A total of 30 C57BL/6 N mice were obtained from Beijing Vital River Laboratory Animal Technology Co., Ltd. (Beijing, China). All mice were housed in a controlled environment and provided with a standard diet for a minimum of 7 days prior to the commencement of experiments. All animals were randomly divided into 5 groups (*n* = 6 per group): control, model, low dose DBT (DBT_low), medium dose DBT (DBT_medium) and high dose DBT (DBT_high) groups. Mice were subjected to a 12-hour fasting period before the procedure, with water available ad libitum. Next, animals were anesthetized with isoflurane inhalation, after which the mice were positioned on a surgical board. The dorsal skin area near the scapulae was prepared, with a square region measuring 4 × 4 cm delineated for the procedure, centered by a 15 mm diameter circular marker. Following disinfection, the full thickness of the skin within the marked circle was excised using a sterile scalpel, and the underlying deep fascia was carefully exposed. After achieving complete hemostasis, 0.1 mL of fecal material was applied to the wound bed and secured with an occlusive dressing consisting of oil gauze and tape. The mice were then returned to their cages to recover from anesthesia. Throughout the study, the mice were monitored daily for signs of successful wound modeling, and the wound sites were periodically dusted with fecal suspension. The modeling process was considered successful if suppurative drainage and a characteristic fecal odor were evident at the wound site within 48 h post-treatment. Control mice did not undergo fecal intervention.

**Fig. 1:**
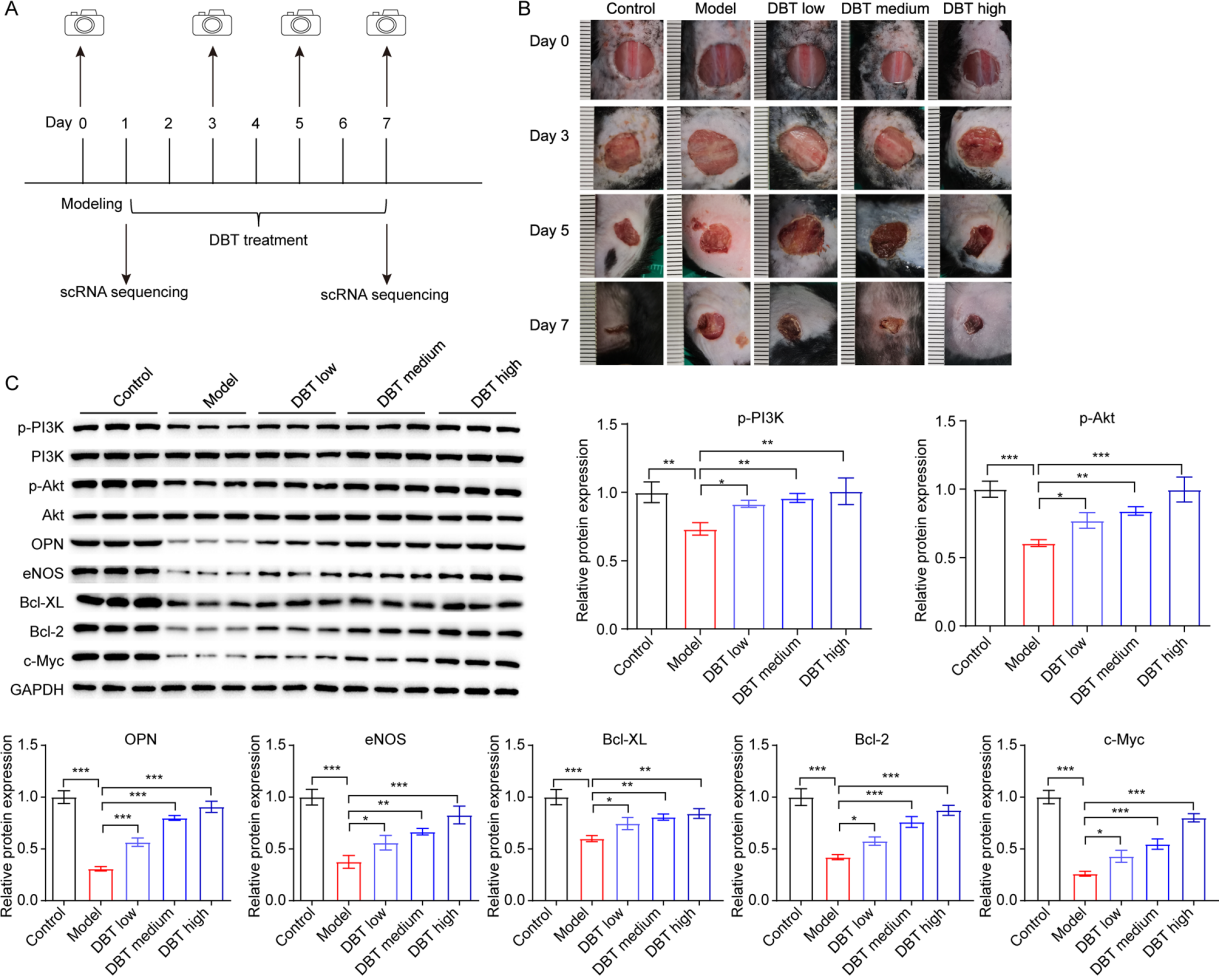
DBT treatment reduced wound area in an animal experimental model of anal fistula. (**A**) Schedule of the animal experiment. (**B**) Representative photos of mouse back skin. (**C**) Western blot assay was conducted to evaluate p-PI3K, p-Akt, OPN, eNOS, Bcl-XL, Bcl-2 and c-Myc levels in the wound tissues of mice (*n* = 3). Error bars indicate standard deviation (SD). *, *P* < 0.05; **, *P* < 0.01; ***, *P* < 0.001. Statistical analysis, one-way ANOVA with Tukey’s post hoc test

On the second day following modeling, mice in the DBT treatment groups (DBT_low, DBT_medium and DBT_high) received drug administration. In this study, according to the dose conversion between mice and humans, the mice in the DBT_low group received 0.10812 g/20 g of DBT (0.5 mL) by gavage once daily; the mice in the DBT_medium group received 0.21624 g/20 g of DBT (0.5 mL) by gavage once daily; the mice in the DBT_high group received 0.32436 g/20 g of DBT (0.5 mL) by gavage once daily. The normal and model groups were given 0.9% physiological saline by gavage (0.5 mL). On days 0, 3, 5, 7, the backs of mice were photographed using a digital camera (Fig. 1A). Seven days later, all mice were sacrificed by cervical dislocation under anesthesia, and the dorsal wound tissue from each mouse was collected.

Additionally, on Day 1 and Day 7, the dorsal wound tissue was collected from one mouse in the model (W, short for wound) and DBT_high (D, short for DBT) groups, respectively, for scRNA sequencing.

### Western blot assay

The concentration of protein was assessed by using the BCA kit. Equal amounts of protein samples underwent sodium dodecyl sulfate-polyacrylamide gel electrophoresis, followed by transfer onto a polyvinylidene fluoride membrane. The membrane was blocked with 5% nonfat milk diluted in TBST for 1 h, after which it was incubated overnight at 4 °C with primary antibodies, including anti-p-PI3K (No. AP0854, ABclonal), anti-PI3K (No. 60225-1-Ig, Proteintech), anti-p-Akt (No. AP0637, ABclonal), anti-Akt (No. A17909, ABclonal), anti-c-Myc (No. ab32072, Abcam), anti-Bcl-XL (No. 26967-1-AP, Proteintech), anti-Osteopontin (OPN, No. 22952-1-AP, Proteintech), anti-eNOS (No. 27120-1-AP, Proteintech), anti-Bcl-2 (No. 26593-1-AP, Proteintech) and anti-GAPDH (No. 10494-1-AP, Proteintech). Following incubation with the appropriate secondary antibody, the reactive bands were visualized using an ECL detection reagent.

### Data collection

Single cell sequencing data of a normal skin tissue from mouse were downloaded from the Gene Expression Omnibus (GEO, https://www.ncbi.nlm.nih.gov/geo/) database (GSE188954) as a normal control (N, short for normal).

### Sequencing data processing and dimensionality reduction

Raw gene expression matrices were initially processed using the Cell Ranger (6.1.2) Pipeline, aligning them with the mouse reference version mm10-2020-A. Subsequently, R software (4.1.2) along with the “Seurat” package (4.3.0) was employed for further analysis. Mitochondrial transcript percentages (percent.mt) were computed and appended as metadata to the Seurat object, prior to cell filtering based on criteria (n_genes > 200; percent_mito < 10%). Subsequent steps included scaling expression values to 10,000 transcripts per cell and applying logarithmic transformation. Correction for the effect of variables (percent.mt) was conducted via the “ScaleData” function using a linear model, followed by dimensionality reduction and clustering using the scaled and centered residuals.

For dimensionality reduction, 2000 genes exhibiting high cell-to-cell variation were identified through the “FindVariableFeatures” function in Seurat. The “RunPCA” function was then applied to reduce dimensionality for each cell, using default parameters on linear-transformed scaled data generated by the “ScaleData” function. The “ElbowPlot” function was subsequently utilized to determine appropriate dimensions for each dataset. To integrate cells from different datasets into a shared space for unsupervised clustering, the harmony algorithm was employed for batch effect correction.

### Cell clustering and reclustering of major cell types

Following batch effect correction and projection of all cells into two-dimensional space using the “RunUMAP” function, a cell graph was initially constructed using the K-Nearest Neighbors (KNN) algorithm. Doublets were discerned by examining cells displaying pronounced and consistent expression profiles from multiple cell types.

For identifying subtypes within macrophage cells at different states, a two-round clustering approach was employed. Initially, macrophages were isolated from the count matrix. Subsequently, akin to the procedure conducted on all cells, dimensionality reduction and cell clustering were executed.

### Gene set scoring

AddModuleScore was utilized to compute the scores of gene sets on individual cells.

### Differential gene expression analysis and functional enrichment analysis

Differential gene expression analysis was carried out using the “FindMarkers” function in Seurat, specifying the parameter “test.use = t”. Subsequently, enrichment analysis for the functions of the differentially expressed genes (DEGs) was performed using “clusterProfiler” (Version 4.7.1) in R language.

### Single-cell pseudotime trajectory analysis

Monocle (Version 2.22.0) endeavors to delineate cellular transitions during differentiation by pseudotemporally profiling scRNA-seq data. Following the input of the count matrix into the “newCellDataSet” function along with clustering information, the data underwent dimensionality reduction using the discriminative dimensionality reduction with trees (DDRTree) method. Subsequently, cells were ordered based on pseudotime.

### Cell–cell communication analysis

CellChat was employed to thoroughly evaluate the overall cell-to-cell communications and quantitatively analyze intercellular communication networks. In summary, the normalized data were inputted into CellChat, utilizing the CellChatDB. mouse database to assess cell-cell communication within our dataset.

### Statistical analysis

For western blot analysis, the data is expressed as means ± standard deviation (SD). One-way analysis of variance (ANOVA) with Tukey’s post hoc test was employed for all statistical comparisons. Statistical significance was defined as *p* < 0.05.

## Results

### DBT treatment reduced wound area in an animal experimental model of anal fistula

After modeling (Day 0), red granulation tissue was observed in the wound area on the backs of mice in the model and DBT_low/medium/high groups, along with a small amount of purulent exudate present at the edge of the wound (Fig. 1B). This observation indicates that the anal fistula-like wounds were successful established.

The PI3K/Akt/eNOS signaling pathway has been demonstrated to play significant role in skin injury [20, 21]. Additionally, OPN, as an upstream regulator of PI3K/Akt signaling, has been reported to be critical in tissue repair [22, 23]. Thus, western blot assay was conducted to evaluate the expression changes of OPN/PI3K/Akt/eNOS signaling. As shown in Fig. 1C, the levels of p-PI3K, p-Akt, OPN, eNOS, Bcl-XL, Bcl-2 and c-Myc were notably reduced in the wound tissues of the model group. Conversely, these proteins were upregulated following DBT treatment, with the most pronounced effects observed in the DBT_high group. Thus, the wound tissue from the mouse in the DBT_high was selected for scRNA sequencing.

### Overall cell cluster distribution of normal (N), wound (W1, W7), and DBT (D1, D7) samples

**Fig. 2:**
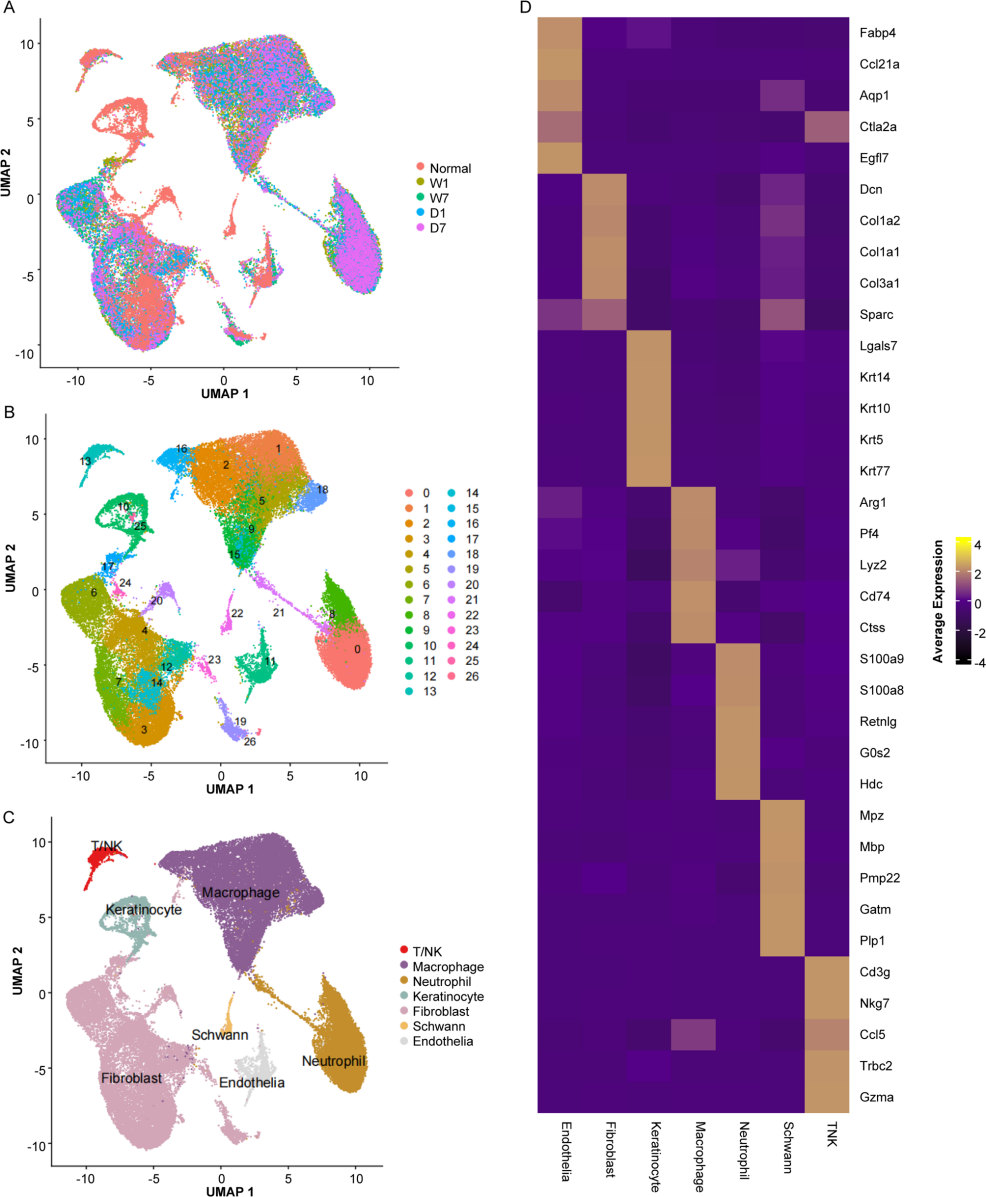
Overview of cell cluster distribution. (**A-C**). Uniform Manifold Approximation and Projection (UMAP) clustering of scRNA-seq from N, W1, W7, D1, D7 samples colored-coded by sample name, cluster name, and cell types, respectively. (**D**). Heapmap of top 5 gene expression levels of each cell type

To explore how DBT influences the polarization process of macrophages within wound tissues, scRNA sequencing was performed on wound mice and DBT-intervened wound mice. A normal sample from public databset GSE188954 was also included. Following the establishment of scRNA sequencing libraries in accordance with the established guidelines, we carried out rigorous quality assessment, data normalization, and scaling procedures. The information of cells and genes were obtained for further analyses (Fig. 2A; Table 1**)**. The clustering was performed by unsupervised Uniform Manifold Approximation and Projection (UMAP), and the cells were clustered into 26 clusters (Fig. 2B) or seven cell types including T/NK, Macrophage, Neutrophil, Keratinocyte, Fibroblast, Schwann, and Endothelia (Fig. 2C). Top 5 genes expression levels for each of these seven cell types were shown in Fig. 2D.

**Table 1:** Summary of single-cell sequencing data

Sample	Cell Count. Raw	Cell Count. Filtered	Median UMI Count. Raw	Median UMI Count. Filtered	Median Gene Count. Raw	Median Gene Count. Filtered	Median Percent MT. Raw	Median Percent MT. Filtered
Normal	9391	8418	8295	9106	2856	3024.5	3.5467321	3.36417079
W1	13,214	12,313	5650.5	6183	1921	2043	2.19416141	2.09609044
W7	11,521	10,340	3387	4328.5	1300	1631	1.72017661	1.51152185
D1	12,243	11,374	6406	6975.5	2075	2194	2.21550856	2.10756554
D7	11,553	9958	4573	5988.5	1627	1981	2.32701812	1.98096863

Abbreviations: Unique Molecular Identifier (UMI), Mitochondrial (MT)

**Fig. 3:**
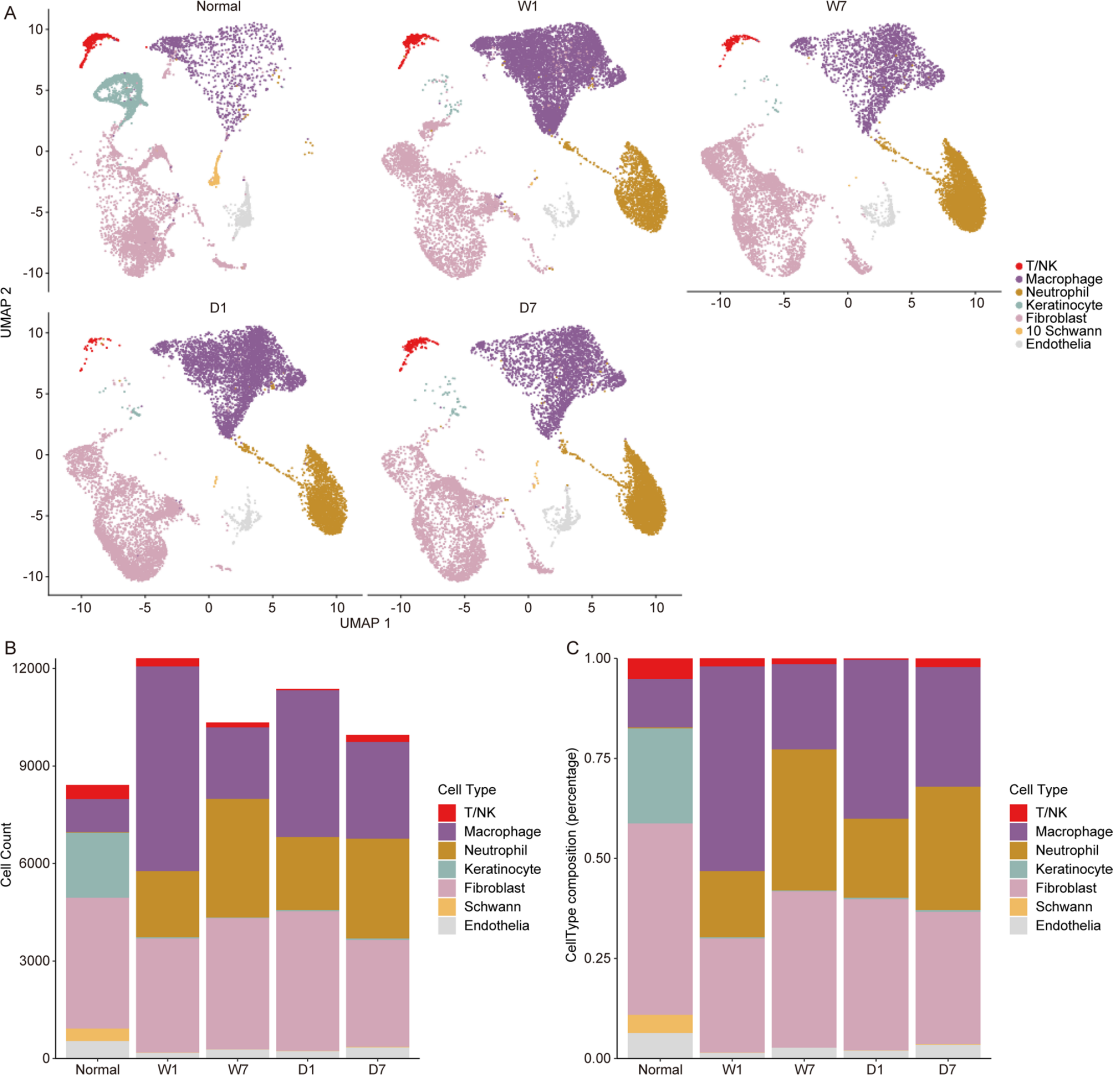
Detailed cell cluster distribution. (**A**). Uniform Manifold Approximation and Projection (UMAP) clustering of scRNA-seq from N, W1, W7, D1, D7 samples individually. (**B-C**). The number and ratio of each cell type from the five samples

The distribution of various cell types across the five samples is delineated in Fig. 3A, with the numerical counts (Fig. 3B) and proportional representations (Fig. 3C) of each cell type corroborated by the raw data enumerated in Tables 2 and 3. Following DBT intervention in sample D1, a noticeable reduction in the T/NK cell count was observed in comparison to sample W1. At the one-week mark, sample W7 exhibited a decrement in T/NK cells compared to W1 sample, whereas sample D7 demonstrated an upsurge relative to D1 (Table 2). A comparative analysis of the macrophage populations in both the wounded (W) and DBT-treated (D) groups revealed an increase in their numbers compared to the normal control sample (Table 2). Within the W group, a pronounced escalation in macrophage numbers was noted in concurrence with wound formation, followed by a swift decline over the subsequent seven days from 6300 to 2201 cells (Table 2). Conversely, in the D group, the decrement was more gradual, reducing from 4515 to 2976 cells from day 1 to day 7. The proportional trends for macrophages in both groups mirrored the numerical patterns (Tables 2 and 3).

**Table 2:** The number of different cell types of N, W1, W7, D1, D7 samples

	T/NK	Macrophage	Neutrophil	Keratinocyte	Fibroblast	Schwann	Endothelia
Normal	437	1017	19	2001	4026	382	536
W1	249	6300	2036	37	3512	6	173
W7	154	2201	3648	21	4037	2	277
D1	44	4515	2249	40	4295	7	224
D7	219	2976	3071	46	3290	15	341

**Table 3:** The ratio of different cell types of N, W1, W7, D1, D7 samples

	T/NK	Macrophage	Neutrophil	Keratinocyte	Fibroblast	Schwann	Endothelia
Normal	5.191256831	12.08125445	0.225706819	23.7704918	47.82608696	4.537894987	6.367308149
W1	2.022252903	51.16543491	16.53536912	0.300495411	28.52269959	0.048728986	1.405019086
W7	1.489361702	21.28626692	35.28046422	0.203094778	39.04255319	0.01934236	2.678916828
D1	0.386847195	39.69579743	19.77316687	0.351679269	37.76156146	0.061543872	1.969403904
D7	2.199236795	29.88551918	30.83952601	0.461940149	33.0387628	0.150632657	3.424382406

The neutrophil count in both W and D groups experienced an increase seven days post-wound construction (Table 2). Additionally, a marked decrement in keratinocyte numbers was observed following the establishment of the wound model, with the D group exhibiting a higher count than the W group (Table 2). Over the seven-day period, a reduction in keratinocytes was noted in the W group, in contrast to an increase in the D group (Table 2). Fibroblasts constituted a significant proportion of the total cell population, with their proportion diminishing immediately after wound formation (Table 3). Over the course of seven days, an increment in fibroblast numbers was observed in the W group, while a decrement was noted in the D group (Table 2). Schwann cells experienced a substantial reduction after wound generation when compared to the normal sample (Tables 2 and 3). Regarding endothelial cells, both numerical and proportional representations were higher in the D7 group than in the W7 group (Tables 2 and 3).

The patterning of all cell types in the W and D groups suggests that DBT may expedite the inflammatory response subsequent to wound occurrence and facilitate an earlier restoration of cell type distribution akin to that of normal conditions in the D group compared to the W group.

### Influence of DBT on the constitute of different macrophages types

**Fig. 4:**
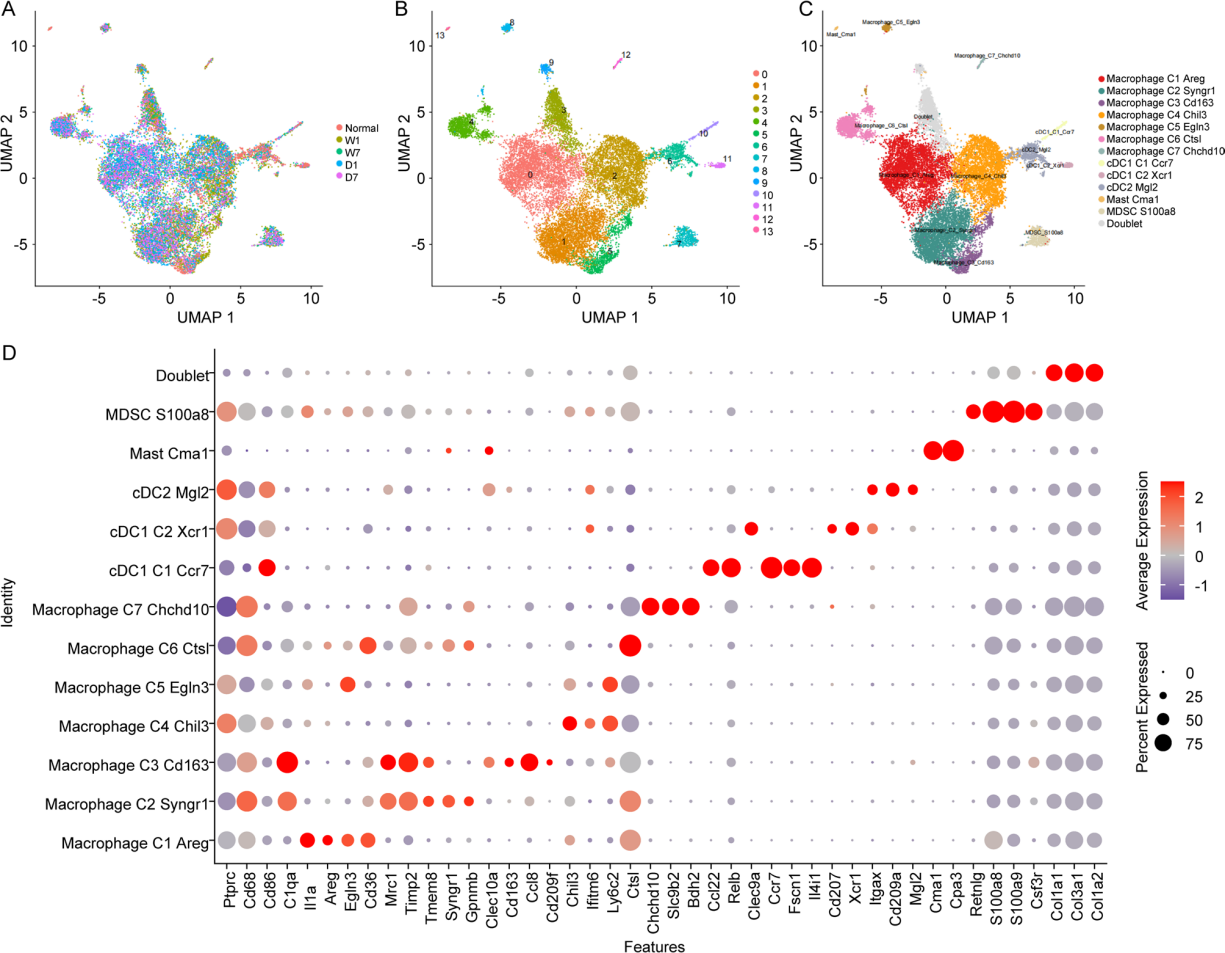
Overview of macrophages cluster distribution. (**A-C**). Uniform Manifold Approximation and Projection (UMAP) clustering of macrophages from N, W1, W7, D1, D7 samples colored-coded by sample name, cluster name, and cell types, respectively. (**D**). Featured gene expression profiles for each cluster from macrophages. The markers for each cluster were as follows: Macrophage_C1_Areg (M1-like (M1C1)): Areg, Il1a and Cd36; Macrophage_C2_Syngr1: Syngr1 and Gpnmb; Macrophage_C3_Cd163 (M2-like (M2C3)): Cd163, Ccl8, Cd209f and Clec10a; Macrophage_C4_Chil3: Chil3, Ifitm6 and Ly6c2; Macrophage_C5_Egln3: Egln3; Macrophage_C6_Ctsl: Ctsl; Macrophage_C7_Chchd10: Chchd10, Slc9b2 and Bdh2; cDC1_C1_Ccr7: Ccr7, Fscn1, Il4i1, Ccl22 and Relb; cDC1_C2_Xcr1: Cd207, Xcr1 and Clec9a; cDC2_Mgl2: Itgax, Cd209a and Mgl2; Mast_Cma1: Cma1 and Cpa3; MDSC_S100a8: Retnlg, S100a8, S100a9 and Csf3r

DBT exhibited an effect in mitigating the rate of reduction of macrophages; however, the specific types of macrophages involved remain to be elucidated. Following the extraction of macrophage cells, standardization and normalization processes were applied. A subset of 2,000 highly variable genes was then selected for Principal Component Analysis (PCA) to reduce the dimensionality of the data. Utilizing 25 principal components, the samples were batch-corrected and subjected to harmony integration analysis. Subsequently, UMAP dimensionality reduction was performed using 20 harmony principal components, as depicted in Fig. 4A. The macrophage cells were subsequently clustered into 13 distinct groups (Fig. 4B), which were designated as Macrophage_C1_Areg (short for M1-like (M1C1); markers: Areg, Il1a and Cd36), Macrophage_C2_Syngr1 (short for C2; markers: Syngr1 and Gpnmb), Macrophage_C3_Cd163 (short for M2-like (M2C3); markers: Cd163, Ccl8, Cd209f and Clec10a), Macrophage_C4_Chil3 (short for C4; markers: Chil3, Ifitm6 and Ly6c2), Macrophage_C5_Egln3 (short for C5; marker: Egln3), Macrophage_C6_Ctsl (short for C6; marker: Ctsl), Macrophage_C7_Chchd10 (short for C7; markers: Chchd10, Slc9b2 and Bdh2), cDC1_C1_Ccr7 (markers: Ccr7, Fscn1, Il4i1, Ccl22 and Relb), cDC1_C2_Xcr1 (markers: Cd207, Xcr1 and Clec9a), cDC2_Mgl2 (markers: Itgax, Cd209a and Mgl2), Mast_Cma1 (markers: Cma1 and Cpa3), MDSC_S100a8 (markers: Retnlg, S100a8, S100a9 and Csf3r), and a doublet associated with Fibroblasts (Fig. 4C). The featured gene expression profiles for these clusters were illustrated in Fig. 4D. The specific expression of markers IL1a, AREG in M1-like (M1C1) indicated this cluster was a classical M1 macrophage. Meanwhile, M2-like (M2C3) was considered as M2 macrophage due to the specific expression of CD163 [24].

**Fig. 5:**
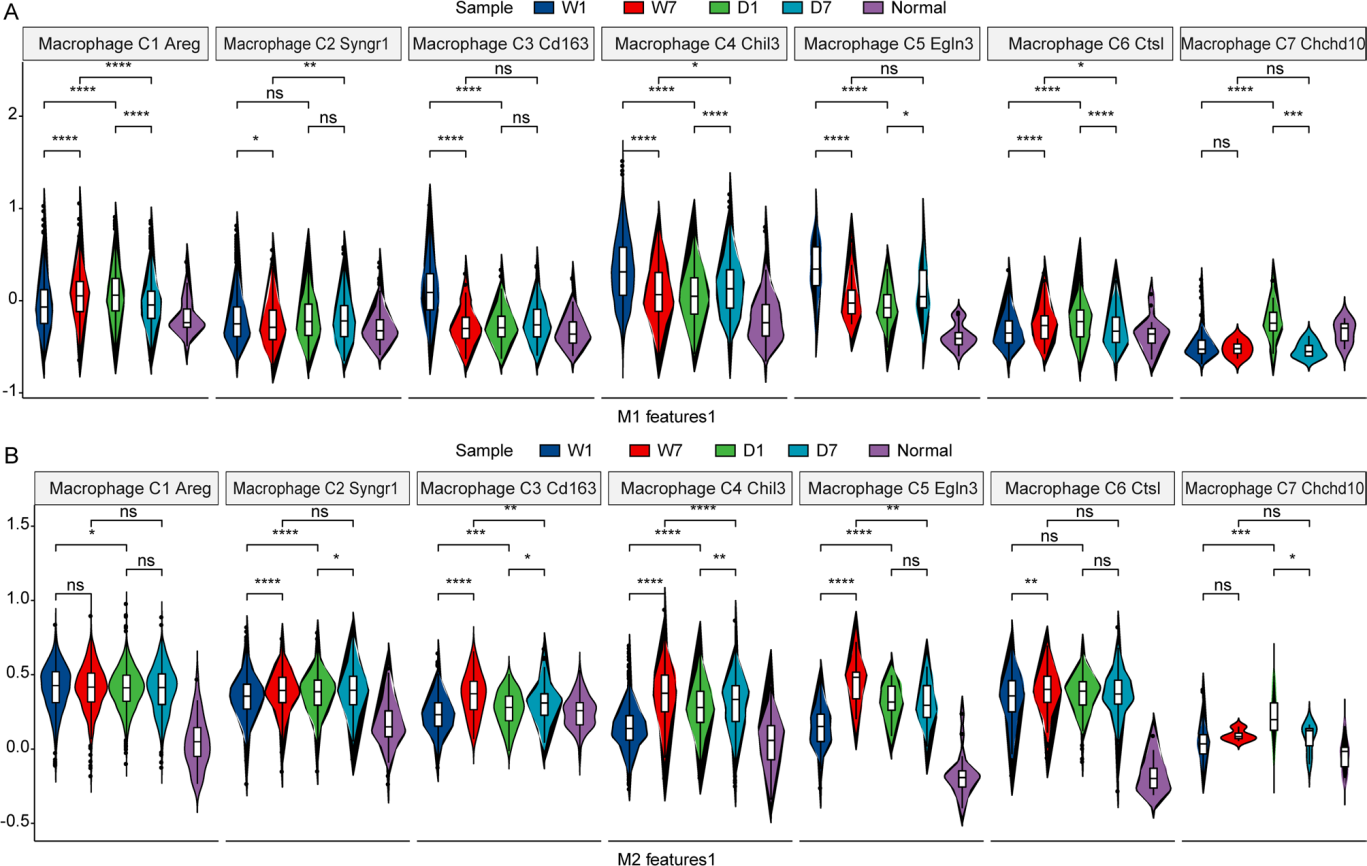
M1 and M2 feature score in various macrophages cell clusters. (**A**). M1 feature score in various macrophages cell clusters. (**B**). M2 feature score in various macrophages cell clusters. The markers for each cluster were as follows: Macrophage_C1_Areg (M1-like (M1C1)): Areg, Il1a and Cd36; Macrophage_C2_Syngr1: Syngr1 and Gpnmb; Macrophage_C3_Cd163 (M2-like (M2C3)): Cd163, Ccl8, Cd209f and Clec10a; Macrophage_C4_Chil3: Chil3, Ifitm6 and Ly6c2; Macrophage_C5_Egln3: Egln3; Macrophage_C6_Ctsl: Ctsl; Macrophage_C7_Chchd10: Chchd10, Slc9b2 and Bdh2; cDC1_C1_Ccr7: Ccr7, Fscn1, Il4i1, Ccl22 and Relb; cDC1_C2_Xcr1: Cd207, Xcr1 and Clec9a; cDC2_Mgl2: Itgax, Cd209a and Mgl2; Mast_Cma1: Cma1 and Cpa3; MDSC_S100a8: Retnlg, S100a8, S100a9 and Csf3r

Focusing on the seven distinct macrophage types, an investigation was conducted to examine the M1 and M2 characteristics, utilizing the established M1/M2 gene sets as referenced in the literature [25]. Within the M1-like (M1C1) cluster, it was observed that the M1 feature score was markedly elevated in sample D1 compared to W1 (Fig. 5A). Following a week, an increase was noted in sample W7 relative to W1; however, a decline in the M1 feature score was detected in the D7 group relative to D1 (Fig. 5A). At the onset of wound establishment, the M1 feature score in sample D surpassed that of W, but this trend reversed after a seven-day period, with the D group exhibiting a subdued score in comparison to the W group (Fig. 5A). The comprehensive score for each gene constituting the M1 feature was provided in Supplementary Fig. 1. In the M2-like (M2C3) cluster, both W and D groups demonstrated a significant enhancement in the M2 feature score seven days post-wound construction (Fig. 5B). The score for each gene included in the M2 feature was detailed in Supplementary Fig. 2.

**Fig. 6:**
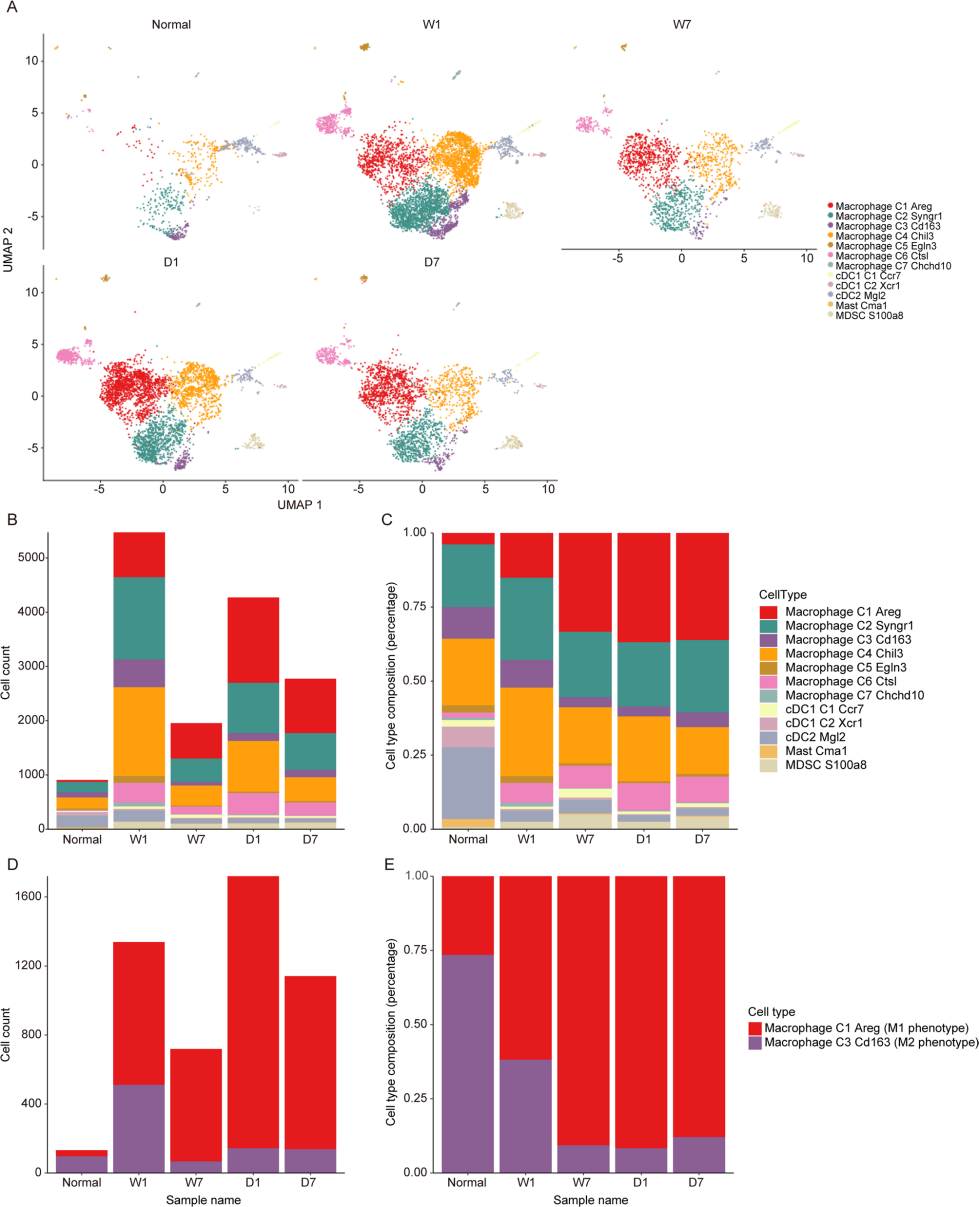
Detailed macrophage cell cluster distribution. (**A**). Uniform Manifold Approximation and Projection (UMAP) clustering of macrophages from N, W1, W7, D1, D7 samples individually. (**B-C**). The number and ratio of each cell type from the five samples. (**D-E**). The number and ratio of Macrophage_C1_Areg (M1-like (M1C1)) and Macrophage_C3_Cd163 (M2-like (M2C3)) from the five samples. The markers for each cluster were as follows: M1-like (M1C1): Areg, Il1a and Cd36; Macrophage_C2_Syngr1: Syngr1 and Gpnmb; M2-like (M2C3): Cd163, Ccl8, Cd209f and Clec10a; Macrophage_C4_Chil3: Chil3, Ifitm6 and Ly6c2; Macrophage_C5_Egln3: Egln3; Macrophage_C6_Ctsl: Ctsl; Macrophage_C7_Chchd10: Chchd10, Slc9b2 and Bdh2; cDC1_C1_Ccr7: Ccr7, Fscn1, Il4i1, Ccl22 and Relb; cDC1_C2_Xcr1: Cd207, Xcr1 and Clec9a; cDC2_Mgl2: Itgax, Cd209a and Mgl2; Mast_Cma1: Cma1 and Cpa3; MDSC_S100a8: Retnlg, S100a8, S100a9 and Csf3r

The distribution of macrophage cells across the five samples was further detailed in Fig. 6A. The numerical count and proportional representation of each macrophage type were systematically summarized in Tables 4 and 5, with corresponding graphical representations provided in Fig. 6B and C. The quantity and proportion of M1-like (M1C1) cells exhibited an increase following wound construction when compared to the normal sample (Tables 4 and 5**)**. Subsequently, a reduction in the count of M1-like (M1C1) cells was observed in both the W and D groups after seven days (Table 4). From the first to the seventh day post-wound, the proportion of M1-like (M1C1) cells in the W group increased from 15.13% to 33.37%, while in the D group, it decreased from 36.91% to 36.17% (Table 5). During the seven days, the proportion of M1-like (M1C1) cells continues to rise in the W group, while it has begun to decline in the D group (Table 5), suggesting that DBT might accelerate the inflammatory response immediately following wound formation and then quickly control this inflammatory reaction. In terms of the M2-like (M2C3) cell type, both W and D groups initially exhibited an increase in cell numbers compared to the normal sample, which was then followed by a decrease (Table 4). Over the course of seven days post-wound construction, the proportion of M2-like (M2C3) cells in the W group decreased from 9.34% to 3.43%, whereas in the D group, the proportion of M2-like (M2C3) cells increased from 3.35% to 4.98% (Table 5). C2 macrophages showed a similar pattern to M2-like (M2C3) in W and D group (Tables 4 and 5).

**Table 4:** The number of different macrophage cell types of N, W1, W7, D1, D7 samples

	Macrophage_C1_Areg	Macrophage_C2_Syngr1	Macrophage_C3_Cd163	Macrophage_C4_Chil3	Macrophage_C5_Egln3	Macrophage_C6_Ctsl	Macrophage_C7_Chchd10	cDC1_C1_Ccr7	cDC1_C2_Xcr1	cDC2_Mgl2	Mast_Cma1	MDSC_S100a8
Normal	35	192	97	204	22	16	7	20	64	219	24	7
W1	828	1518	511	1637	123	368	71	45	31	202	3	136
W7	652	431	67	369	17	147	4	59	16	88	7	97
D1	1577	927	143	935	27	394	15	40	14	93	4	104
D7	1003	677	138	438	26	241	8	35	13	68	10	116

**Table 5:** The ratio of different macrophage cell types of N, W1, W7, D1, D7 samples

	Macrophage_C1_Areg	Macrophage_C2_Syngr1	Macrophage_C3_Cd163	Macrophage_C4_Chil3	Macrophage_C5_Egln3	Macrophage_C6_Ctsl	Macrophage_C7_Chchd10	cDC1_C1_ Ccr7	cDC1_C2_ Xcr1	cDC2_Mgl2	Mast_Cma1	MDSC_ S100a8
Normal	3.858875413	21.16868798	10.69459757	22.49173098	2.425578831	1.764057332	0.771775083	2.205071665	7.056229327	24.14553473	2.646085998	0.771775083
W1	15.12881418	27.73615933	9.336744016	29.91046958	2.247396309	6.723917413	1.297277544	0.822218162	0.566416956	3.690845971	0.054814544	2.484926
W7	33.36745138	22.05731832	3.428863869	18.88433982	0.870010235	7.523029683	0.204708291	3.019447288	0.818833163	4.503582395	0.358239509	4.964176049
D1	36.90615493	21.69435993	3.346594898	21.88158203	0.631874561	9.220688041	0.351041423	0.936110461	0.327638661	2.176456822	0.093611046	2.433887199
D7	36.17021277	24.41399207	4.976559683	15.79516769	0.937612694	8.690948431	0.288496213	1.262170934	0.468806347	2.452217815	0.360620267	4.183195096

Furthermore, as shown in Fig. 6D and E, compared to the W1 group, the proportion of M1-like (M1C1) cells was increased, and the proportion of M2-like (M2C3) cells were reduced in the W7 group. Meanwhile, compared to the W7 group, DBT treatment elevated M2-like (M2C3) cell proportion, but decreased M1-like (M1C1) cell proportion (Fig. 6D and E). These findings implied that DBT might influence macrophages polarization and rapidly suppress inflammation and promote anti-inflammatory processes.

### Potential functions of M1 and M2 macrophages related DEGs

For the purpose of understanding potential function or pathways working in M1-like (M1C1) and M2-like (M2C3) cells, we performed differential expression analysis to identify significantly differentially expressed genes (DEGs) between each cluster and all other clusters (Supplementary Table 1). The marker genes for each cellular subtype demonstrated elevated expression levels relative to other subtypes (Supplementary Table 1). Next, we then performed KEGG and GO analyses on the DEGs of M1-like (M1C1) and M2-like (M2C3) screened from other cell types in macrophage cluster.

**Fig. 7:**
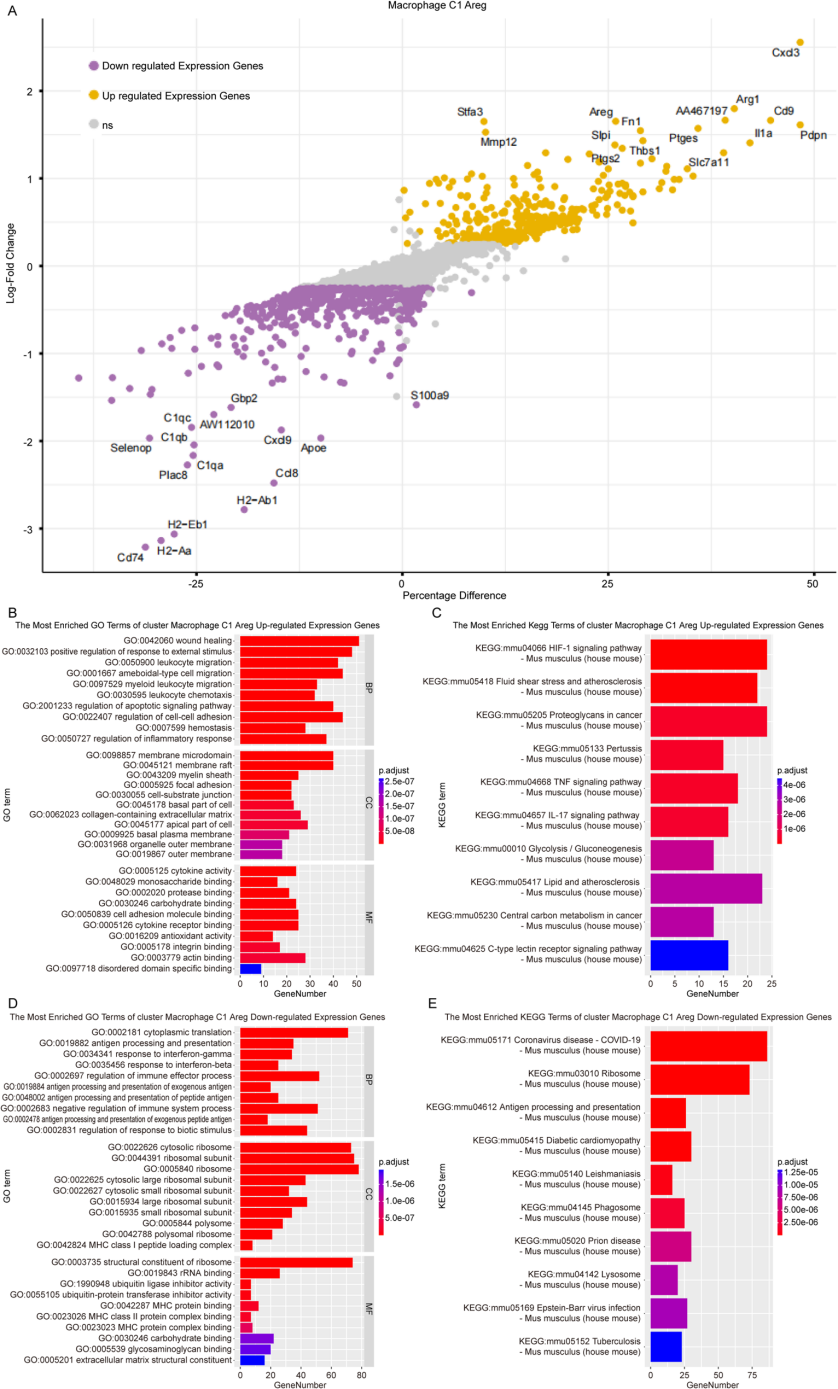
Potential functions and pathways related to M1-like (M1C1). (**A**). Volcano plot of DEGs between M1-like (M1C1) and other macrophages clusters. (**B-C**). Top 10 enriched GO terms and KEGG pathways of upregulated DEGs. (**D-E**). Top 10 enriched GO terms and KEGG pathways of downregulated DEGs

In the M1-like (M1C1) subtype, marker genes of M1-like (M1C1) macrophages, such as AREG and IL1a, exhibited increased expression compared to other types, as illustrated in Fig. 7A and detailed in Supplementary Table 1. The upregulated DEGs were significantly associated with GO terms related to wound healing, positive regulation of response to external stimuli, and membrane microdomains, as depicted in Fig. 7B. Furthermore, these DEGs were enriched on multiple KEGG pathways, including the HIF-1 signaling pathway and the TNF signaling pathway, as shown in Fig. 7C. Downregulated DEGs were linked to processes such as cytoplasmic translation and ribosome (Fig. 7D and E). Detailed information on functional enrichment analysis could be seen in Supplementary Table 2.

**Fig. 8:**
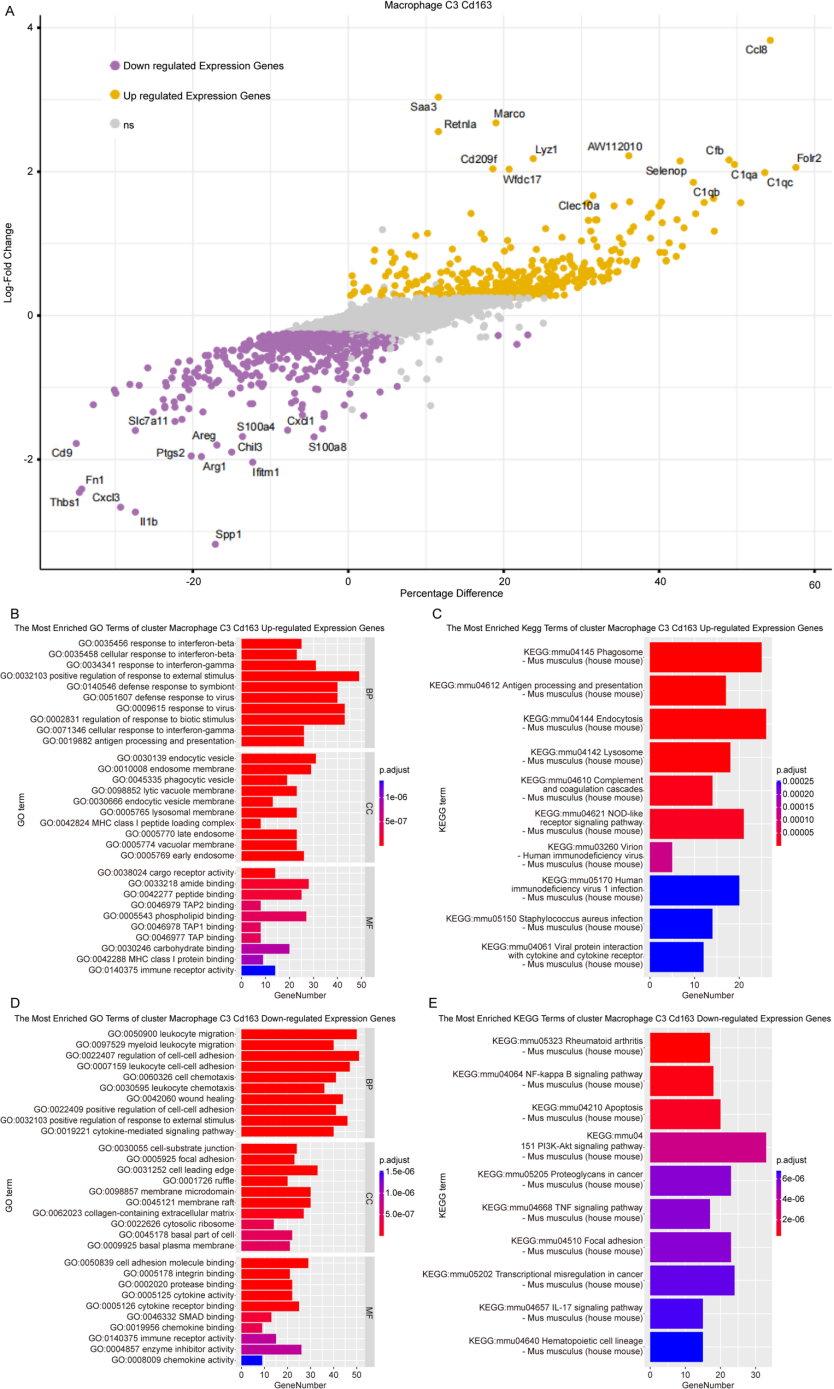
Potential functions and pathways related to M2-like (M2C3). (**A**). Volcano plot of DEGs between M2-like (M2C3) and other macrophages clusters. (**B-C**). Top 10 enriched GO terms and KEGG pathways of upregulated DEGs. (**D-E**). Top 10 enriched GO terms and KEGG pathways of downregulated DEGs

For the M2-like (M2C3) subtype, the DEGs unique to this macrophage type were displayed in Fig. 8A with detailed information in Supplementary Table 1. The upregulated DEGs were enriched on GO entries associated with the response to interferon-beta, positive regulation of response to external stimuli, endocytic vesicles, and cargo receptor activity, among others, as shown in Fig. 8B. Additionally, these DEGs were prominently involved in KEGG pathways related to phagosome function and antigen processing and presentation (Fig. 8C). Meanwhile, downregulated DEGs in the M2-like (M2C3) subtype were associated with processes such as leukocyte migration, regulation of cell-cell adhesion, wound healing, cell-substrate junctions, and cell adhesion molecule binding (Fig. 8D). Furthermore, they were linked to pathways including the NF-kappa B signaling pathway and apoptosis, as indicated in Fig. 8E. Detailed information was provided in Supplementary Table 3.

*3.5 Pseudo-time trajectory analysis revealed significant role of DBT treatment in modulating macrophage polarization and facilitating the wound healing process*.

**Fig. 9:**
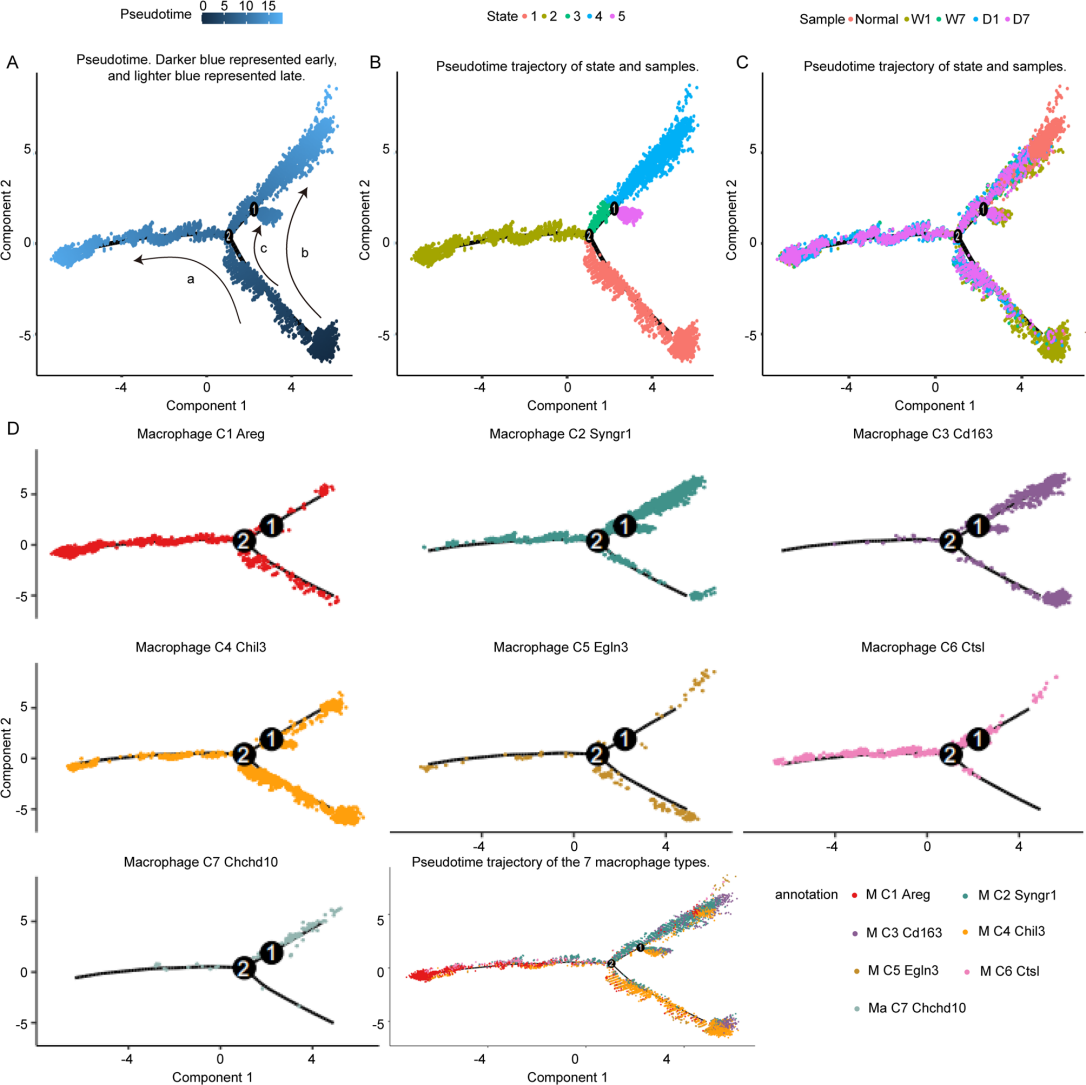
Pseudotime trajectory analysis of the macrophage type. (**A**). Pseudotime. Darker blue represented early, and lighter blue represented late. (**B-C**). Pseudotime trajectory of state and samples. (**D**). Pseudotime trajectory of the 7 macrophage types

Analysis of pseudo-time trajectories is instrumental in elucidating the developmental kinetics and temporal progression of macrophage polarization, facilitating the tracking of sequential alterations within distinct macrophage types following wound formation and subsequent DBT treatment. By constructing a pseudotime trajectory (Fig. 9A), the progression of states (Fig. 9B), samples (Fig. 9C), and macrophage types (Fig. 9D) were simulated, revealing the dynamic landscape of macrophage polarization and wound healing. The trajectory was bifurcated into three primary branches, labeled as “a,” “b,” and “c” (Fig. 9A).

In the “a” branch, the trajectory traced a transition from W1 to D7, indicative of a developmental shift from C4 to the M1-like (M1C1) subtype (Fig. 9D). The “b” branch encapsulated the healing process, starting from the initial wound state (W1), through the recovery phases (D1, W7, D7), culminating in the normalization state (N) (Fig. 9C). Along this branch, the macrophage types evolved from C4 to C2, with M2-like (M2C3) emerging as the dominant subtype by the end of the trajectory (Fig. 9D). Conversely, the “c” branch depicted a scenario where the wound state (W1) was followed by a recovery process (D1, W7, D7). Still, it ultimately failed to reach a state of complete healing (W1) (Fig. 9C). This analysis highlighted the significant role of DBT treatment in modulating macrophage polarization and facilitating the wound healing process, as evidenced by the distinct trajectories and the ultimate fate of the macrophage subtypes in the context of tissue repair and inflammation resolution.

### Cell-cell communication analysis within different cell types

Cell-cell interaction analysis in single-cell sequencing reveals complex communication networks among individual cells, providing valuable insights into intercellular signaling pathways and microenvironmental dynamics. Utilizing the CellChat tool, we conducted comprehensive cell-cell interaction analysis across all cells within the macrophage cluster (Supplementary Fig. 3). We observed active interactions of fibroblasts with various cell types, while keratinocytes displayed significant self-interaction patterns.

Furthermore, we investigated ligand-receptor pairs specific to M1-like (M1C1) and M2-like (M2C3) subpopulations. Notably, both M1-like (M1C1) and M2-like (M2C3) demonstrated close interactions with cDC clusters mediated by amyloid precursor protein (App)-Cd74 pairs (Supplementary Fig. 4A-B). Additionally, interactions involving Fn1-Sdc4, Fn1-Sdc1, and Fn1-Cd44 were frequently observed in M1-like (M1C1) with other cell types, whereas such interactions were rare in M2-like (M2C3) with other cell types (Supplementary Fig. 44A-B).

## Discussion

DBT is a traditional Chinese medicine extensively utilized in the management of various conditions, attributed to its anti-inflammatory effects [26]. Its function for wound healing has also been detected [27]. The duration of recovery from an anal fistula surgery varies based on the treatment undergone and the complexity level of the patient’s condition [28]. In our study, scRNA sequencing was performed to explore the underlying mechanism of DBT recovery function.

Macrophages play a crucial role in orchestrating the wound healing process by transitioning from predominantly pro-inflammatory states (M1-like phenotypes) observed early after injury, to anti-inflammatory states (M2-like phenotypes) that emerge later to regulate skin repair and wound closure [16]. In this research, pro-inflammatory M1 type macrophages were stimulated by DBT as soon as the wound was constructed, then they were rapidly controlled and anti-inflammatory M2 type increased to accelerate tissue repair. C4 (CHIL3) and C2 (MARC1) macrophages were considered as M2-like macrophages [29], and they displayed a similar pattern to M2-like (M2C3) in W and D groups. Pseudotime trajectory analysis in this research also revealed the effect of DBT on macrophage polarization. Zhang et al. reported that in a wound model using diabetic rats, DBT reduced the size of the wounds, the presence of inflammation with Notch signaling pathway activated, and promoted the development of granulation tissue, ECM synthesis, and CD31 deposition [27]. It has been shown that the Notch signaling pathway promotes the repair of damaged tissues through the modulation of macrophage-mediated inflammatory responses [30]. These findings demonstrated that DBT may play a role in promoting in wound healing in mice with anal fistula through mediating macrophage polarization.

According to previous research, dried DBT extract contains ferulic acid (FA) and ligustilide derived from *Angelica sinensis* roots, calycosin and formononetin derived from *Astragalus membranaceus* roots [31–34]. FA, a compound naturally present in various fruits and vegetables, has demonstrated the ability in facilitating the recovery of wounds in diabetic rat models, probably due to lipid peroxidation inhibition, and elevation of catalase, superoxide dismutase, glutathione and nitric oxide, as well as zinc and copper in serum [35]. FA can reduce the production of macrophage inflammatory protein-2 and TNF-α induced by lipopolysaccharide which is related to M1 type [36]. FA is able to prompt a shift in the activation state of microglia/macrophages from the pro-inflammatory “M1” to the anti-inflammatory “M2” phenotype, thereby reducing inflammation. The mechanism underlying this effect may involve FA’s inhibition of the ROS/NF-κB pathway [37]. Ligustilide has the capacity to reduce inflammation by suppressing the production of nitric oxide and prostaglandin E2 (PGE2) [38]. Furthermore, DBT is rich in flavonoids, including calycosin and formononetin, which are considered essential and valuable constituents [39]. Laboratory-based studies demonstrated that calycosin reduced the Ti-triggered shift towards M1 macrophage activation, encouraged the M2 macrophage polarization, and consequently improved the bone-forming capabilities of MC3T3-E1 cells [40]. Calycosin modulates macrophage inflammatory reactions by affecting the JNK and NF-κB signaling cascades [41]. Formononetin can regulate the polarization of macrophages and impede the JAK/STAT signaling mechanism, leading to inflammation inhibition [42]. These monomeric compounds isolated from the the roots of *Angelica sinensis* and *Astragalus membranaceus* exhibit anti-inflammatory or promote polarization toward the anti-inflammatory M2 macrophage phenotype. These findings further support the involvement of macrophages in the wound-healing mechanism of DBT.

In certain contexts, feature genes of M1-type macrophages may be expressed at higher levels in M2-type macrophages due to a variety of factors, and vice versa. The plasticity of macrophages allows them to adjust their phenotype and function in response to different environmental signals and stimuli [43], which means that genes typically associated with M1 macrophages may also be expressed in M2 macrophages to accommodate specific physiological or pathological conditions. The polarization of macrophages is often considered a continuum with M1 and M2 representing two extremes, yet the activation and polarization process is fluid with multiple intermediate states [44]. Research methodologies and data analysis can also impact the interpretation of gene expression results. Furthermore, in chronic infections, macrophages may exhibit polarization characteristics different from those in healthy tissue [45], where M1-type genes may be expressed in M2 macrophages to adapt to the unique microenvironment and immune regulatory demands of the disease. In summary, the polarization state of macrophages is dynamic and complex, and the expression of M1-type characteristic genes in M2 macrophages may reflect the diversity and adaptability of macrophage functions, and vice versa. A deeper understanding of the mechanisms behind these phenomena requires further in-depth research and analysis.

PI3K/Akt signaling is crucial for wound healing [46, 47]. Additionally, this pathway has been demonstrated to play a critical role in the polarization of macrophages towards an M2-like phenotype [48]. Mechanistically, the activation of either the PI3K/Akt1 or PI3K/Akt3 signaling pathways can enhance macrophage M2 polarization by increasing the levels of IL-10 and TGF-β [49]. Notably, Gu et al. found that the absence of Akt3 in macrophages resulted in a diminished capacity to facilitate wound healing [50]. These findings underscore the significant relationship between PI3K/Akt signaling and macrophage M2 polarization in wound healing. In our study, we observed that DBT could accelerate wound healing in mice with anal fistula through activation of PI3K/Akt signaling. Additionally, DBT has the potential to promote macrophage M2 polarization and thereby accelerating wound healing. Thus, we propose that DBT treatment may accelerate wound healing after anal fistula by the promotion of macrophage M2 polarization, likely mediated through the activation of the PI3K/Akt signaling pathway (Supplementary Fig. 5).

However, this study also has some limitations. First, although we established an anal fistula-like wound model on the dorsum of mice that allowed precise control over wound size, depth, and contamination, it does not fully replicate the complex anatomical and physiological structure of a true human anal fistula. Therefore, in the future, we plan to establish an animal model of anal fistula that more closely mimics the clinical anatomical and physiological environment, thereby further validating the therapeutic effects and mechanisms of DBT. Second, we acknowledge that the scRNA-seq data in this study were generated from a single mouse per group, which limits our ability to account for potential inter-individual biological variation. Future studies incorporating additional biological replicates will be essential to assess such variability and to validate the consistency of the proportions of the observed cell populations.

## Conclusions

In our exploration of regulatory effects of DBT on macrophage polarization in wound healing using scRNA sequencing technology, macrophage’s trend of transition to an anti-inflammatory M2 or M2-like phenotype was observed, thereby accelerating the wound healing process. Pro-inflammatory M1 macrophages were stimulated at the beginning of DBT treatment but controlled rapidly. Additionally, the pseudotime trajectory analysis and cell-cell communication studies further substantiated the dynamic and interactive nature of macrophage polarization under the influence of DBT. Furthermore, DBT could enhance wound healing in mice with anal fistula in vivo by activating of PI3K/Akt signaling. Thus, our findings suggest that DBT treatment may promote wound healing following anal fistula by facilitating macrophage M2 polarization, likely through the activation of the PI3K/Akt signaling pathway, which was shown to be upregulated by DBT treatment. These insights contribute to a deeper understanding of the immunomodulatory actions of DBT and highlight its potential application in facilitating wound healing, particularly after anal fistula surgery.

## Supplementary Information

Below is the link to the electronic supplementary material.

**Supplementary Material 1**: **Supplementary Fig. 1**. The comprehensive score for each gene constituting the M1 feature.

**Supplementary Material 2**: **Supplementary Fig. 2**. The comprehensive score for each gene constituting the M2 feature.

**Supplementary Material 3**: **Supplementary Fig. 3** Cell-cell interaction analysis. Interaction heatmap of different cell types. The column names in the heatmap represent the names of cells expressing receptor genes, while the row names represent the names of cells expressing ligand genes. The bar chart above represents the sum of the corresponding column numbers. The numbers displayed in the bars on the right show the number of interactions between cells. The bar chart on the right represents the sum of the corresponding row numbers.

**Supplementary Material 4**: **Supplementary Fig. 4**. Ligand-receptor pairs analysis. (A). Ligand-receptor pairs between Macrophage_C1_Areg (M1-like (M1C1)) and other cell types. The vertical axis represents pairs of ligand and receptor genes, while the horizontal axis represents pairs of ligand-expressing cells and receptor-expressing cells. The color of the points represents the magnitude of the communication probability value, and the size of the points represents the magnitude of the *P*-value. (B). Ligand-receptor pairs between Macrophage_C3_Cd163 (M2-like (M2C3)) and other cell types.

**Supplementary Material 5**: **Supplementary Fig. 5**. Mechanisms of DBT-mediated wound healing in mice with anal fistula. DBT may promote wound healing following anal fistula through facilitating macrophage M2 polarization by activating PI3K/Akt signaling. Furthermore, DBT can reduce wound area following anal fistula through activating OPN/PI3K/Akt/eNOS signaling.

**Supplementary Material 6**: **Supplementary Table 1**. Differential gene expression analysis between each cluster and all other clusters.

**Supplementary Material 7**: **Supplementary Table 2**. Functional enrichment analysis on DEGs between Macrophage_C1_Areg (M1-like (M1C1)) and other macrophages clusters.

**Supplementary Material 8**: **Supplementary Table 3**. Functional enrichment analysis on DEGs between Macrophage_C3_Cd163 (M2-like (M2C3)) and other macrophages clusters.

Supplementary Material 9

## Data Availability

Data is provided within the manuscript or supplementary information files.
