# Supplementary figures and images for: Single-cell RNA sequencing reveals that Danggui Buxue Tang decoction facilitates wound healing after anal fistula by promoting M2 macrophage polarization
Source: Hereditas. 2025 Oct 9;162:204. doi: 10.1186/s41065-025-00578-2 (PMC12512633; doi:10.1186/s41065-025-00578-2)

# Ccl5

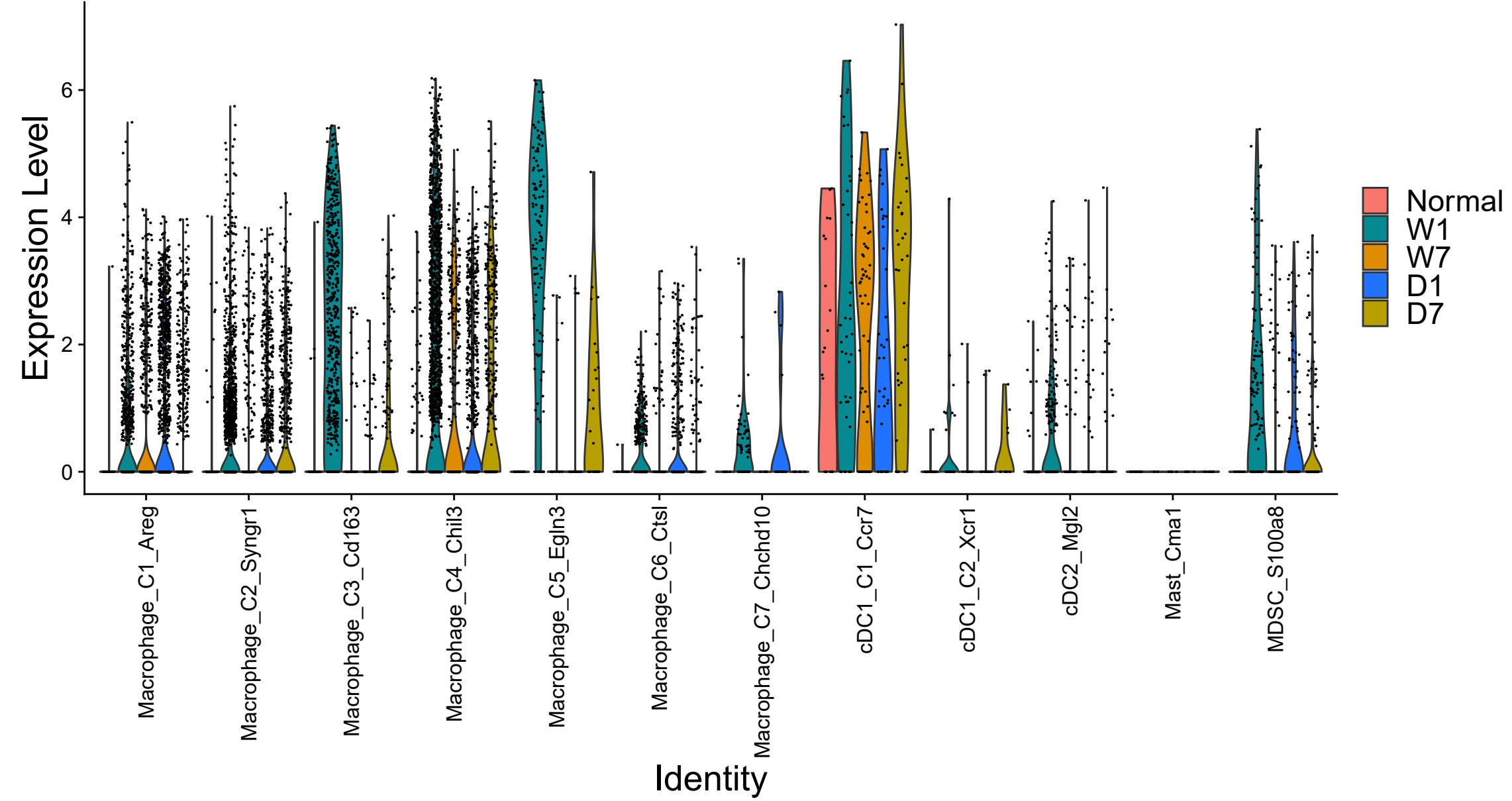

# Ccr7

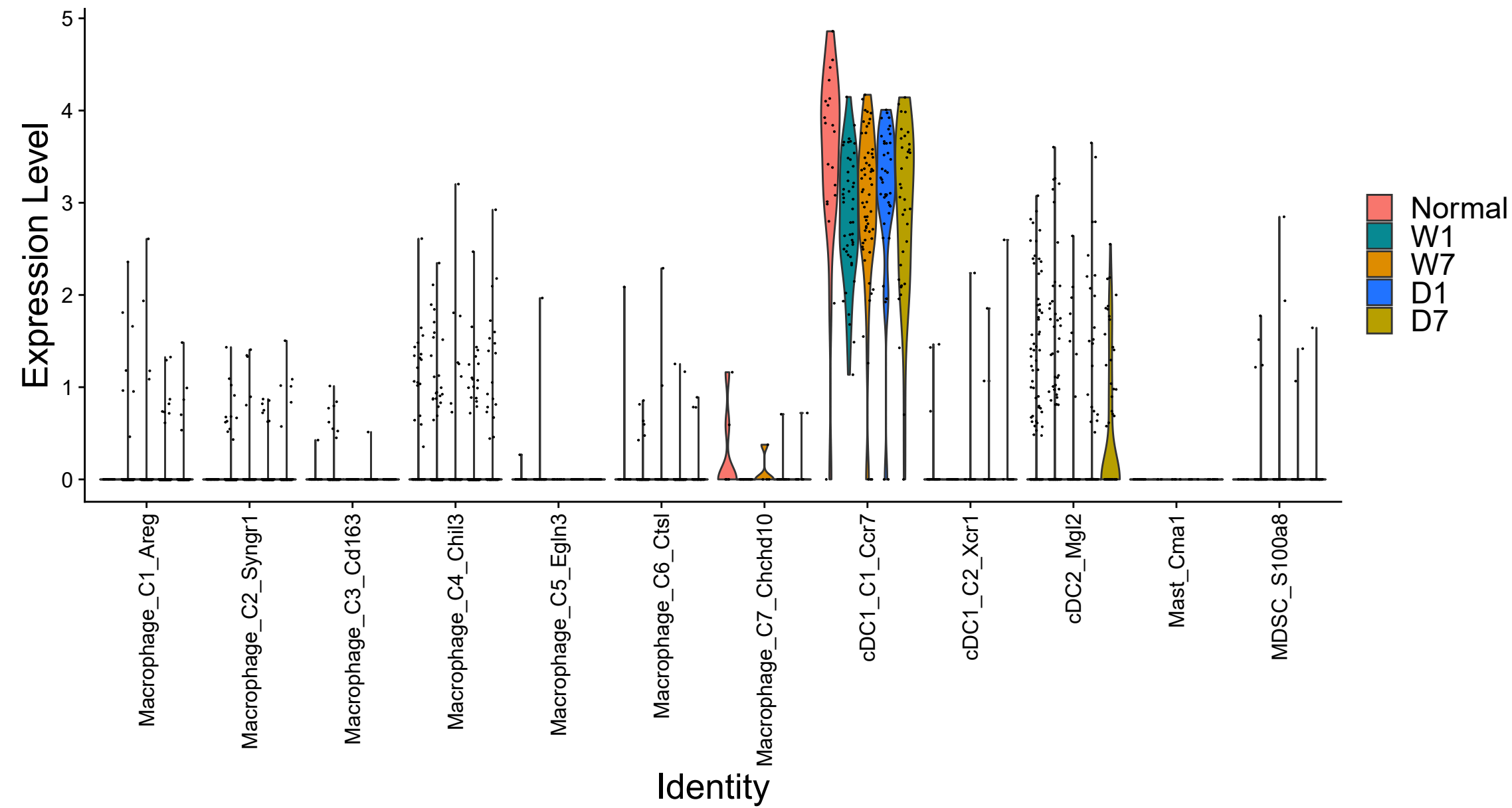

Cd40

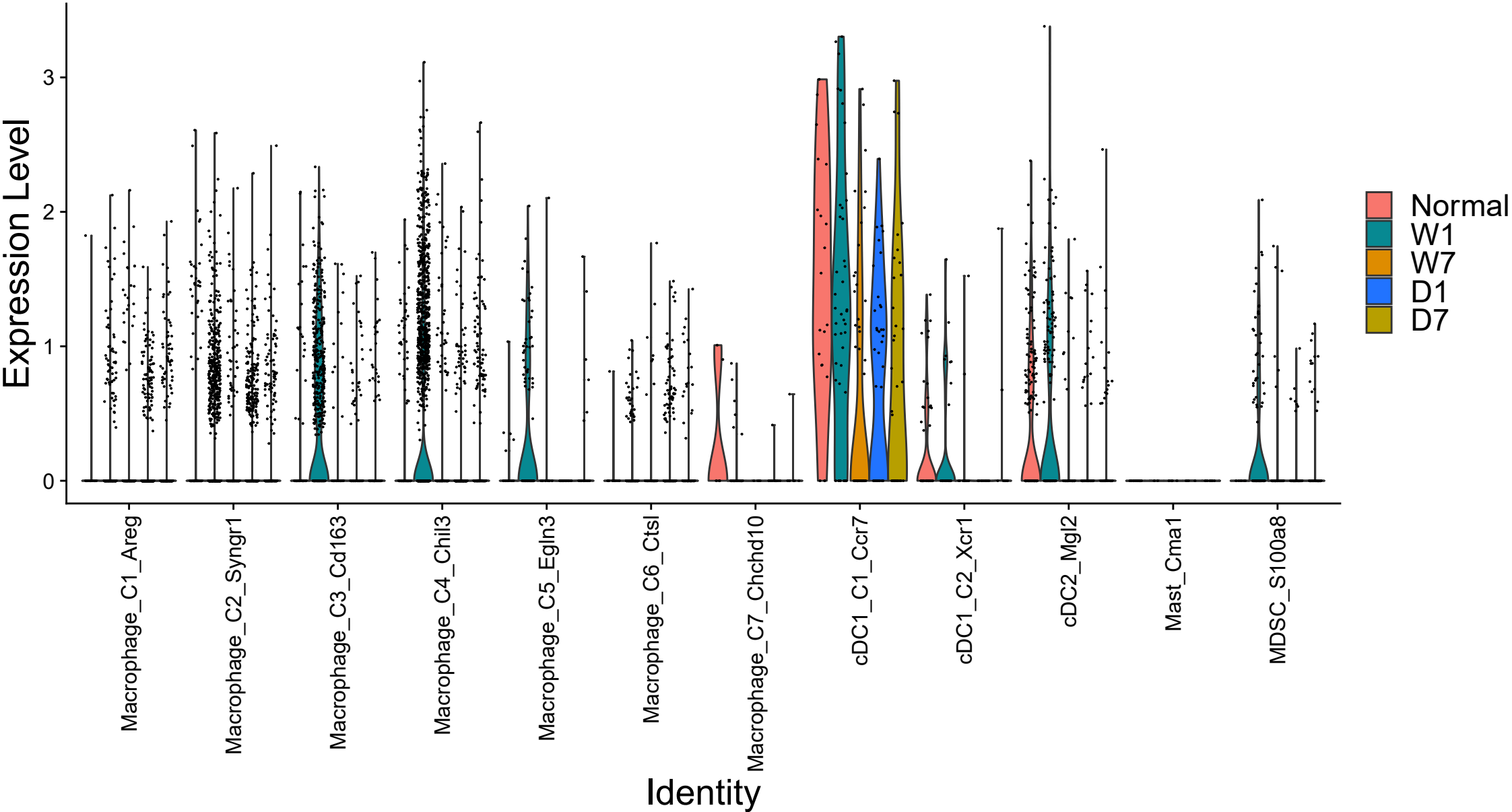

Cd86

Expression Level

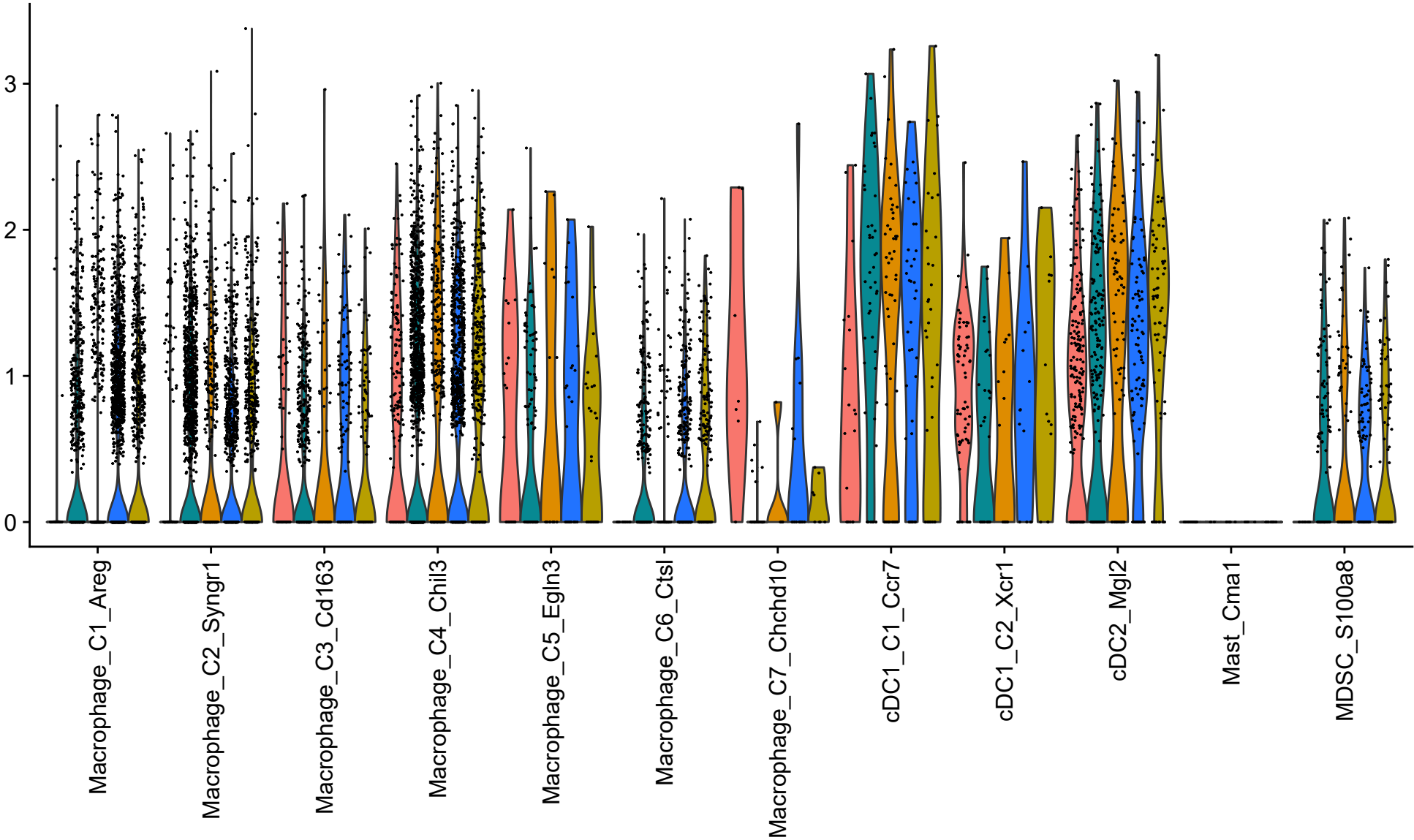

Normal  
W1  
W7  
D1  
D7

Identity

# Cxcl9

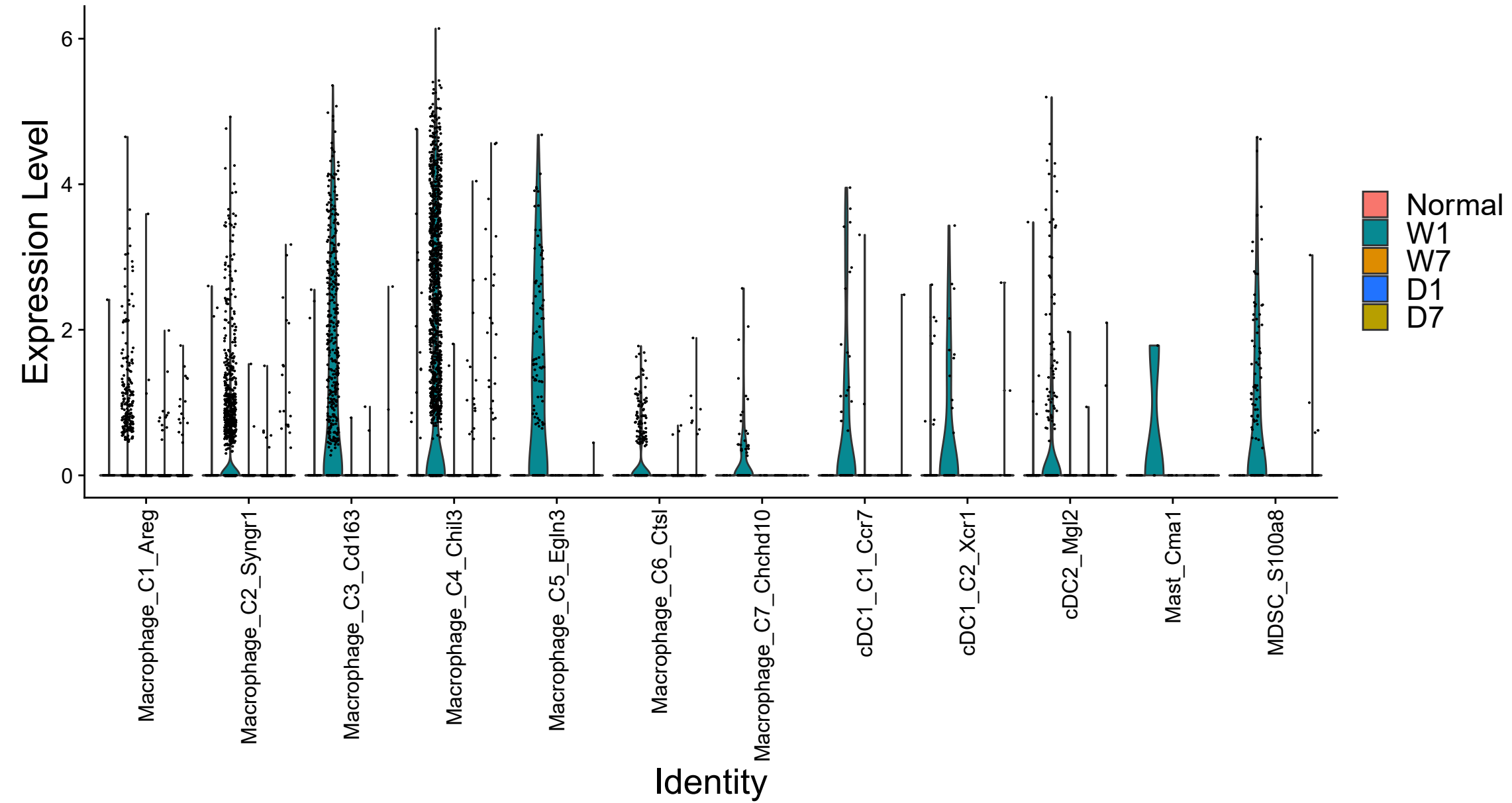

# Cxcl10

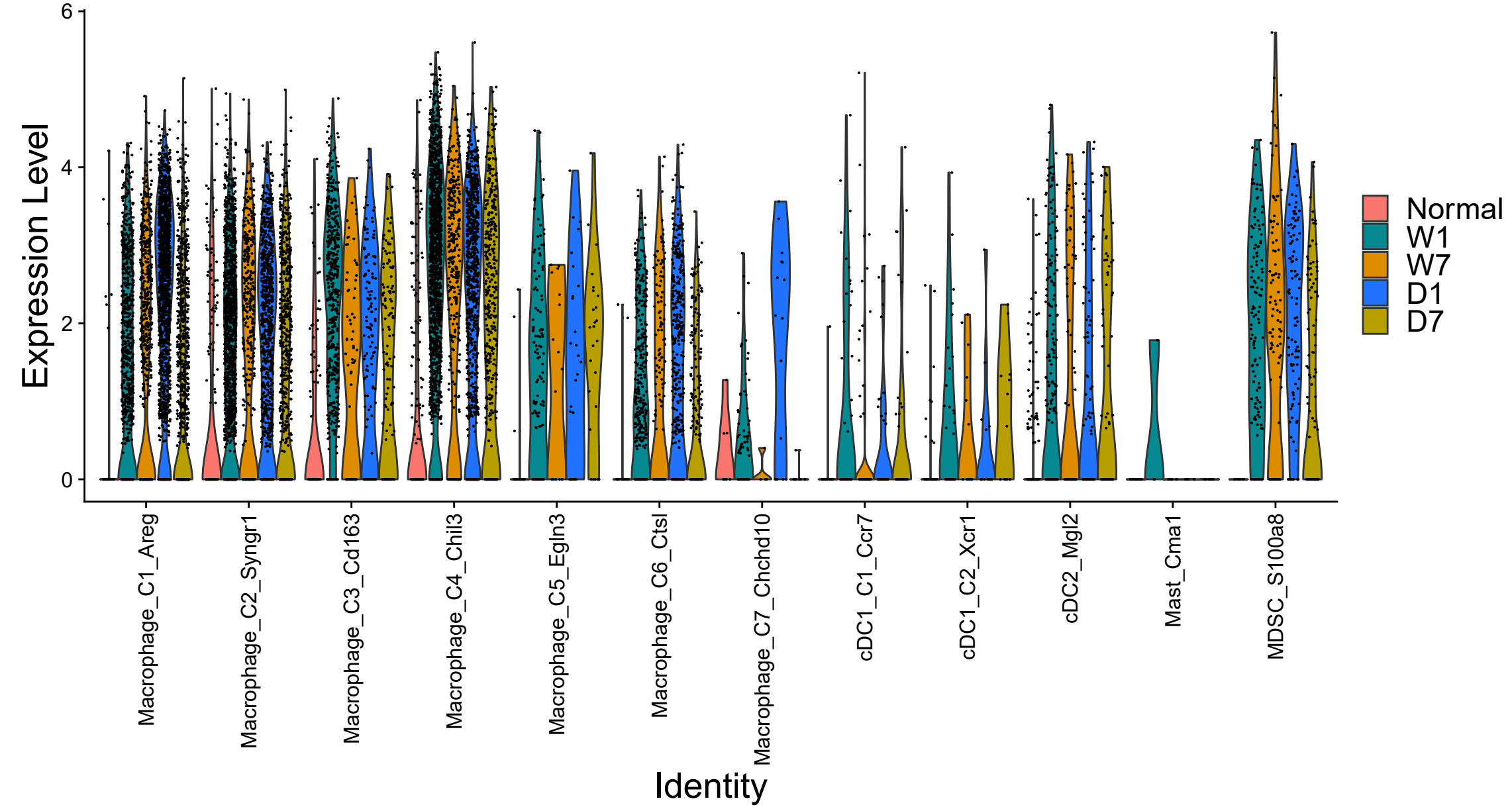

# Ido1

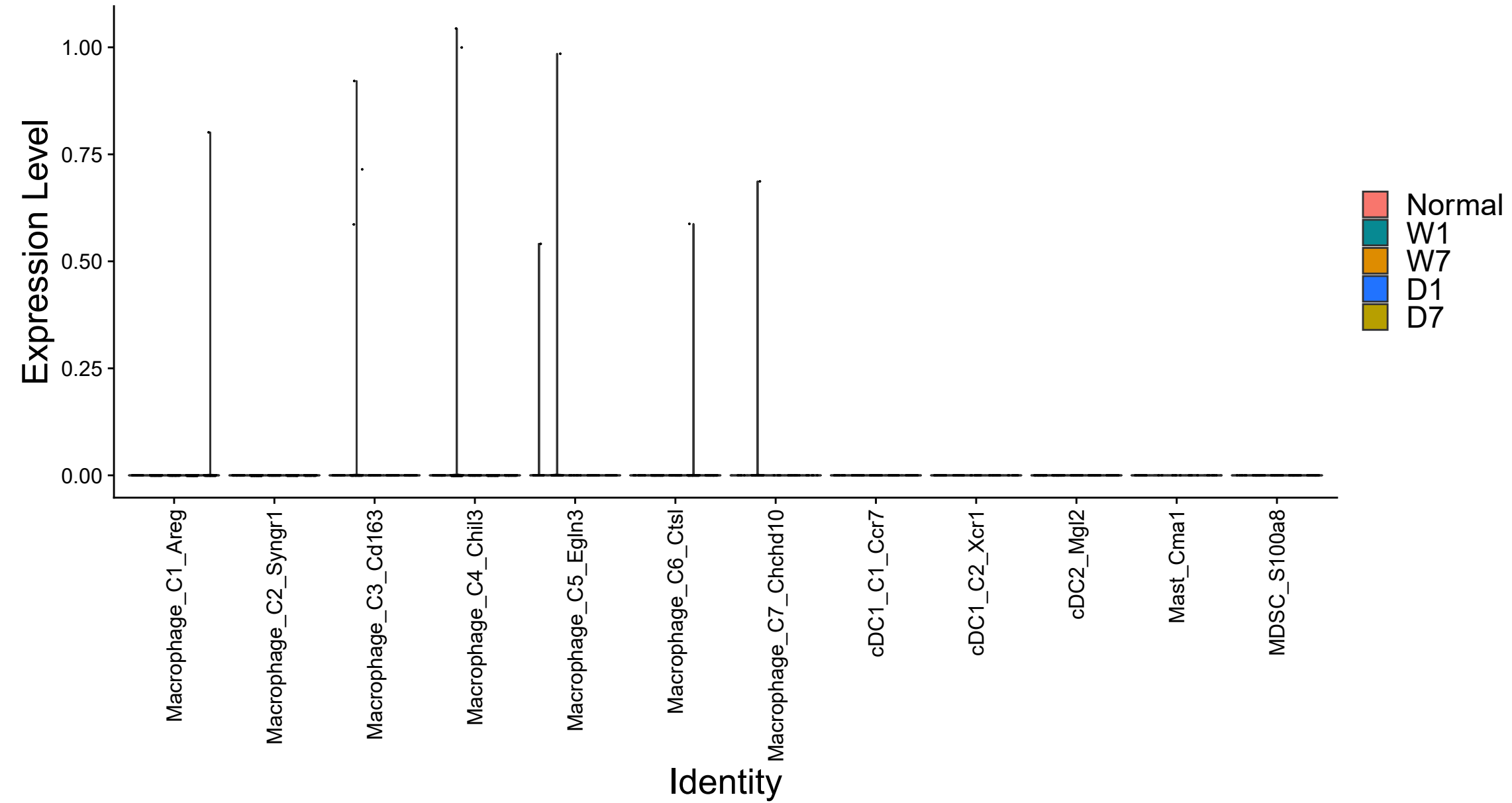

II1a

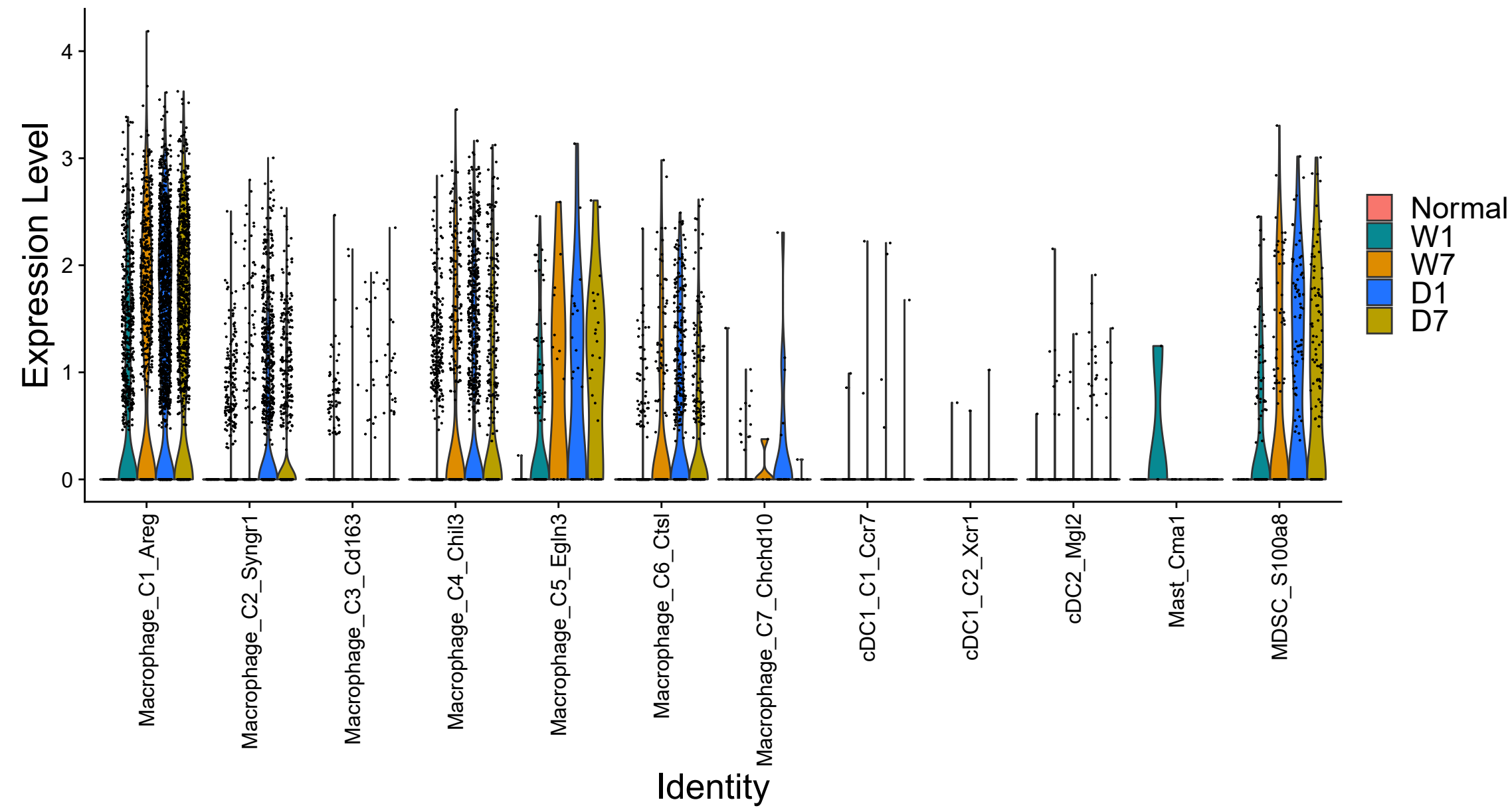

II1b

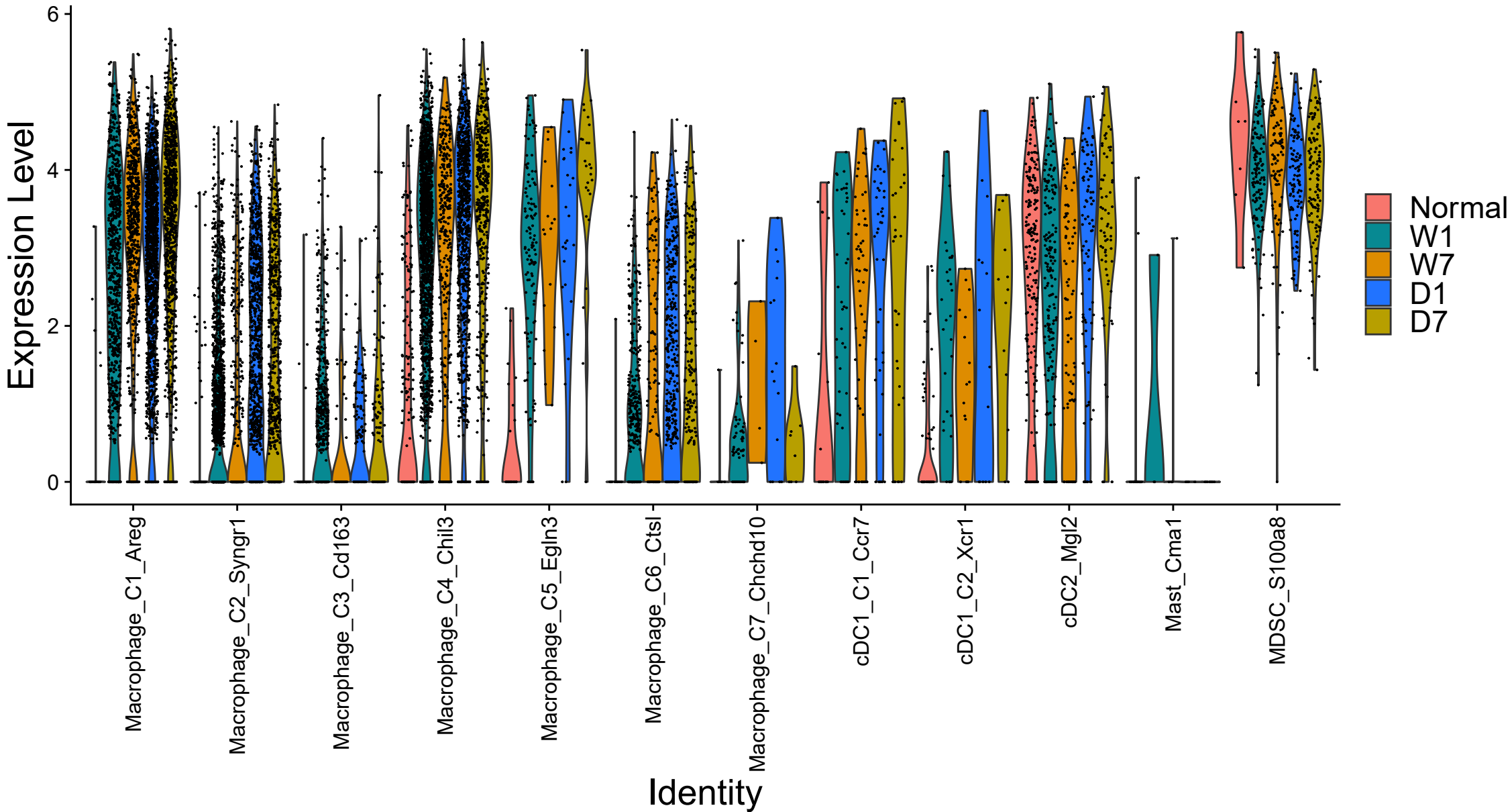

II6

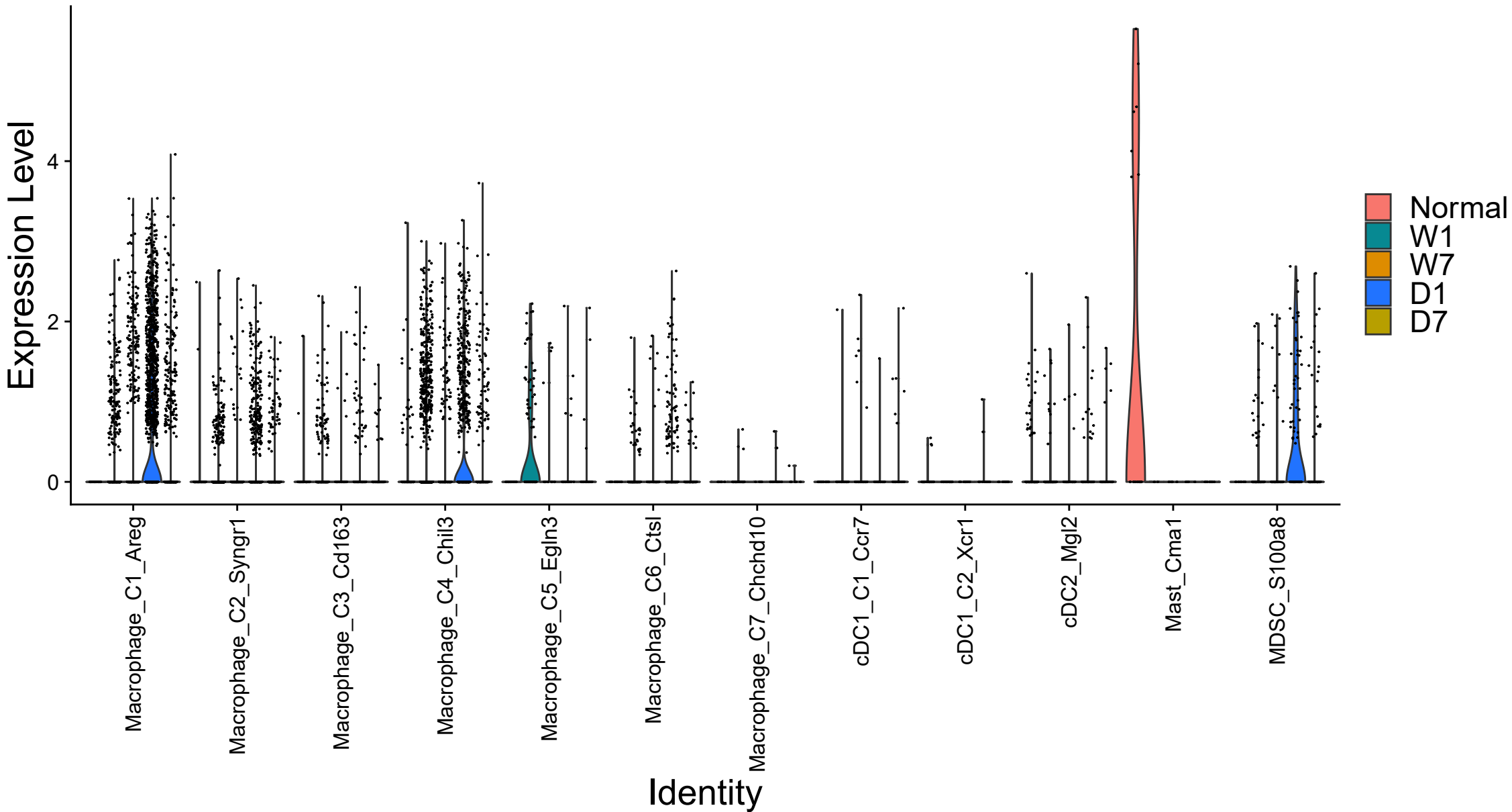

Irf1

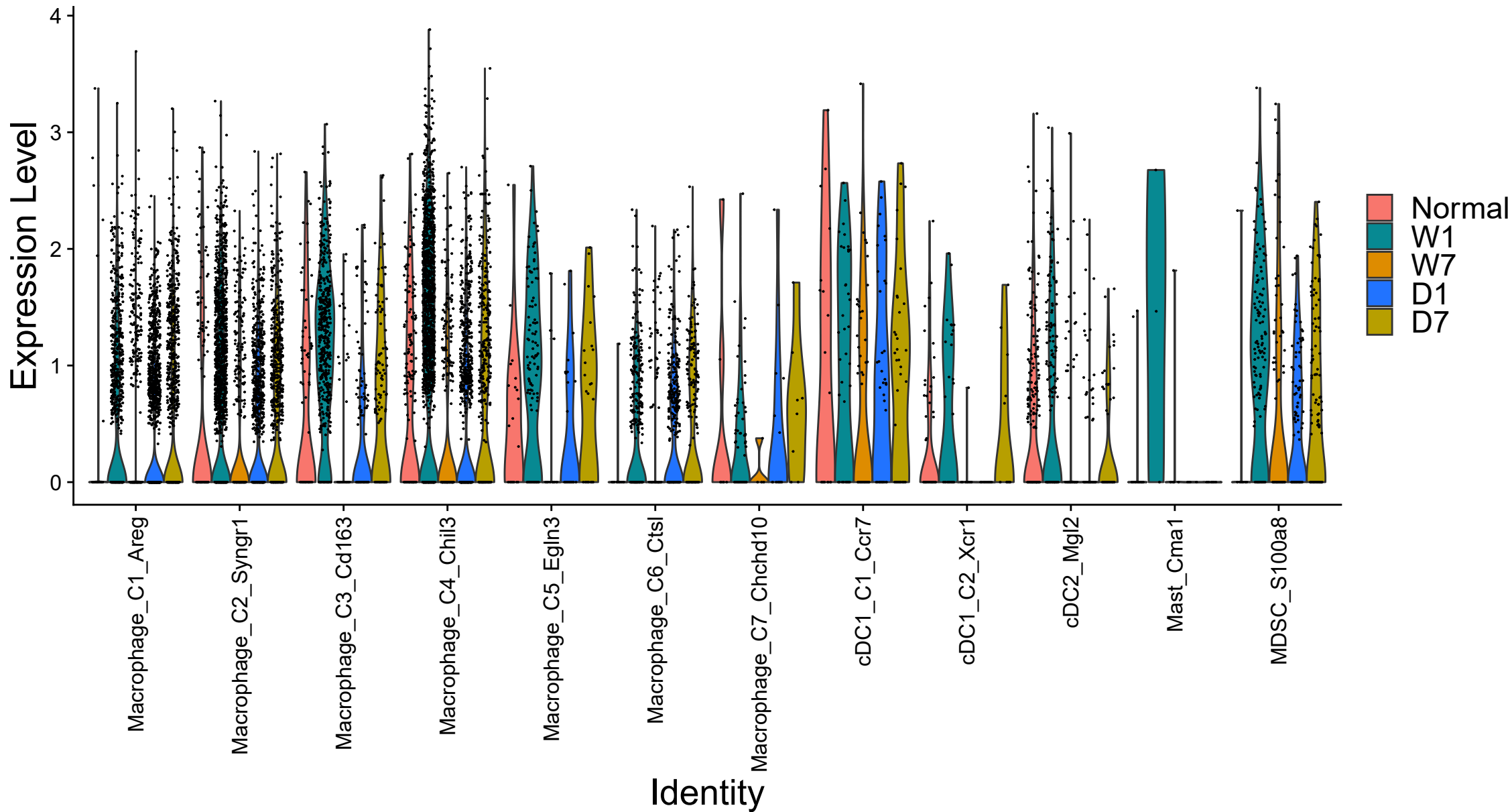

# Irf5

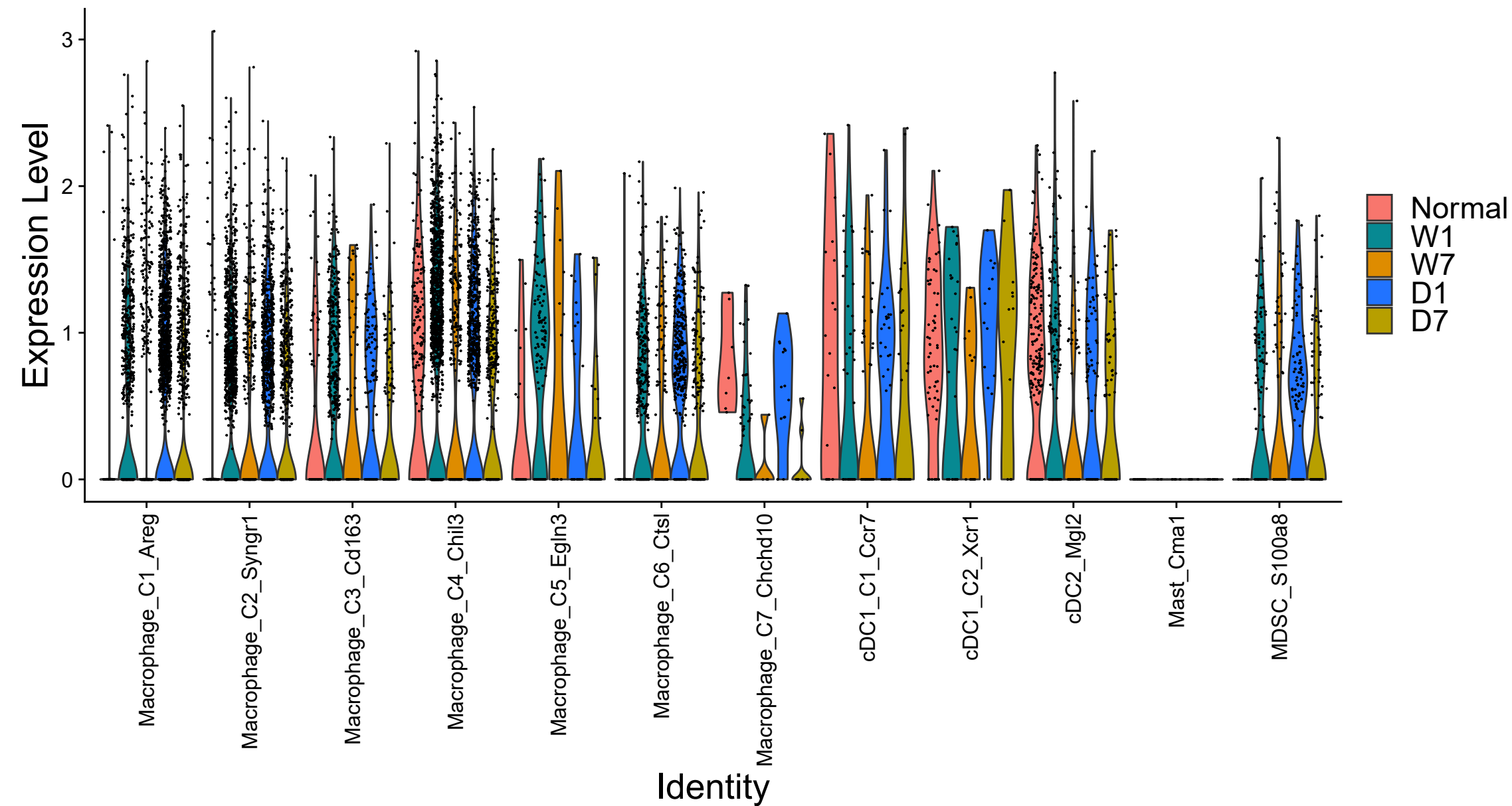

# Kynu

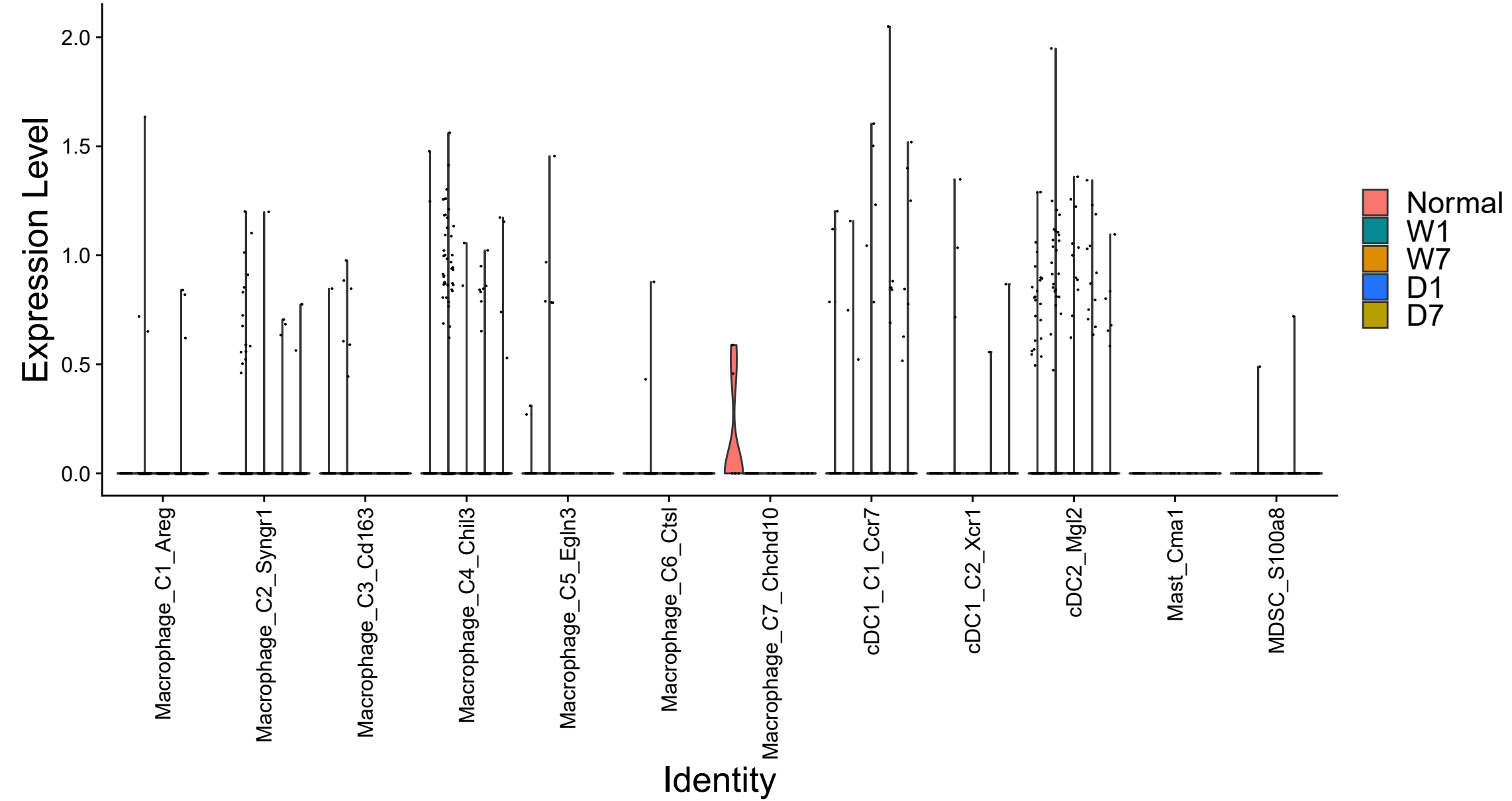

Supplement: Supplementary file 1 — Supplementary Material 1: Supplementary Fig. 1. The comprehensive score for each gene constituting the M1 feature. [file 41065_2025_578_MOESM1_ESM.pdf]

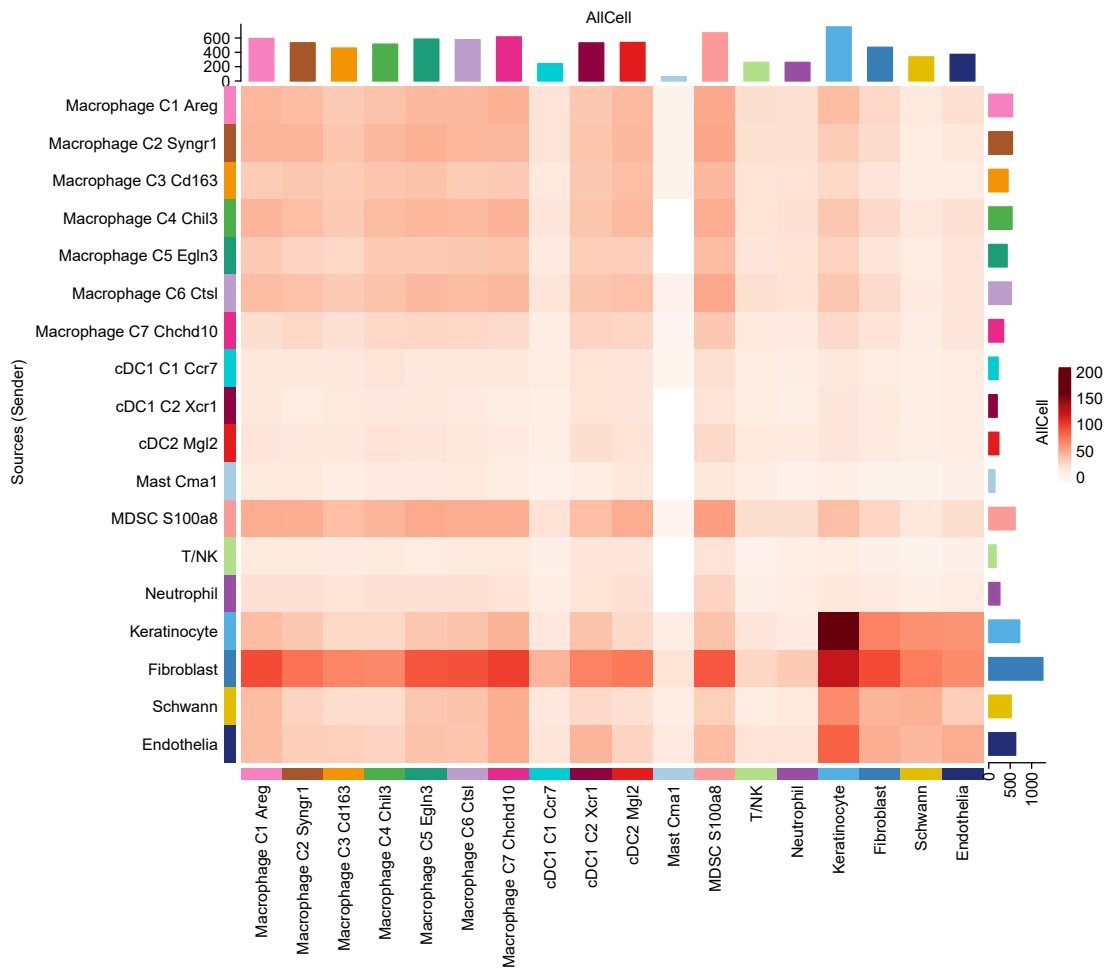

Supplement: Supplementary file 3 — Supplementary Material 3: Supplementary Fig. 3 Cell-cell interaction analysis. Interaction heatmap of different cell types. The column names in the heatmap represent the names of cells expressing receptor genes, while the row names represent the names of cells expressing ligand genes. The bar chart above represents the sum of the corresponding column numbers. The numbers displayed in the bars on the right show the number of interactions between cells. The bar chart on the right represents the sum of the corresponding row numbers. [file 41065_2025_578_MOESM3_ESM.pdf]

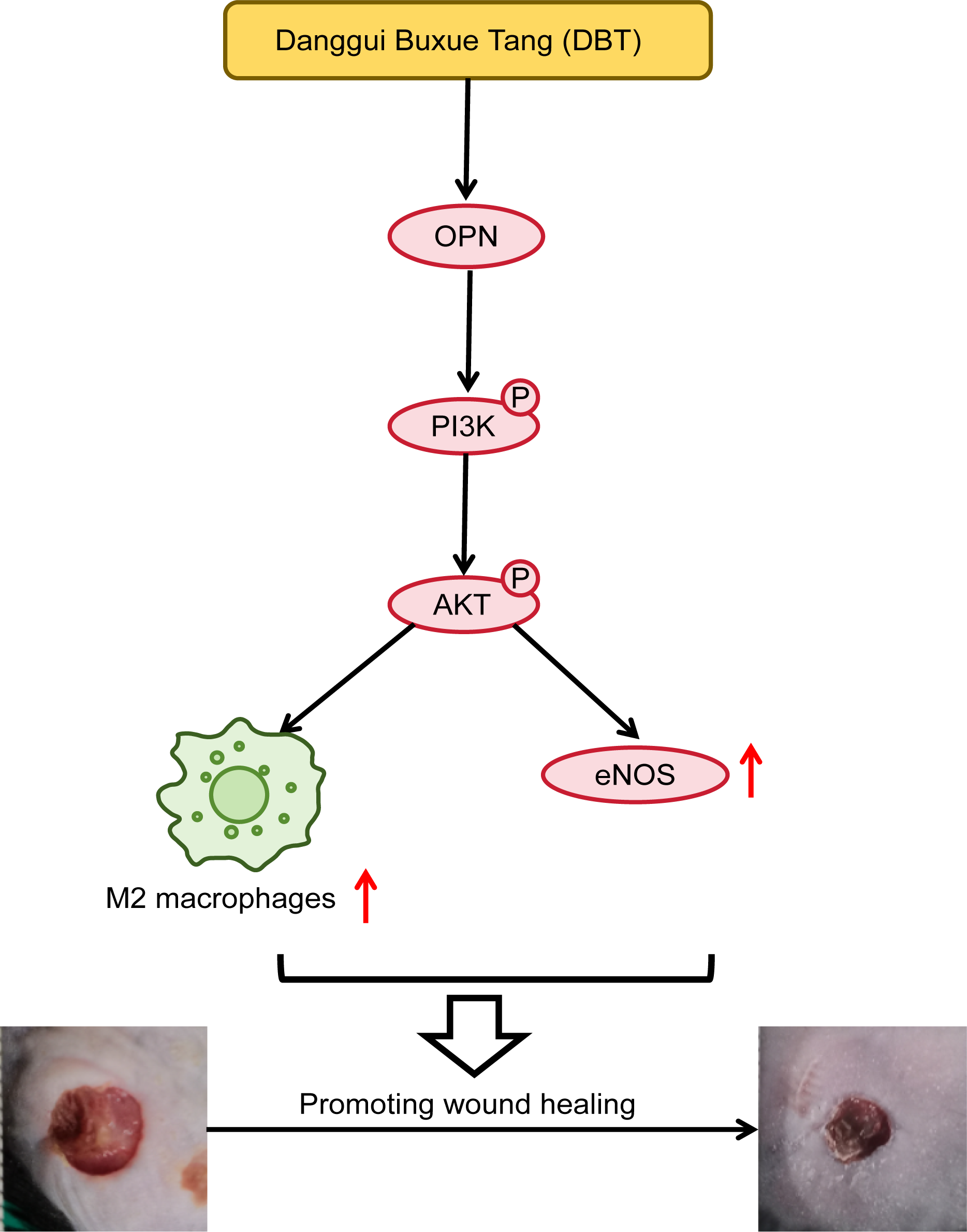

Supplement: Supplementary file 5 — Supplementary Material 5: Supplementary Fig. 5. Mechanisms of DBT-mediated wound healing in mice with anal fistula. DBT may promote wound healing following anal fistula through facilitating macrophage M2 polarization by activating PI3K/Akt signaling. Furthermore, DBT can reduce wound area following anal fistula through activating OPN/PI3K/Akt/eNOS signaling. [file 41065_2025_578_MOESM5_ESM.tif]
