# Supplementary material for: Single-cell RNA sequencing reveals that Danggui Buxue Tang decoction facilitates wound healing after anal fistula by promoting M2 macrophage polarization
Source: Hereditas. 2025 Oct 9;162:204. doi: 10.1186/s41065-025-00578-2 (PMC12512633; doi:10.1186/s41065-025-00578-2)
Supplement: Supplementary file 2 — Supplementary Material 2: Supplementary Fig. 2. The comprehensive score for each gene constituting the M2 feature. [file 41065_2025_578_MOESM2_ESM.pdf]

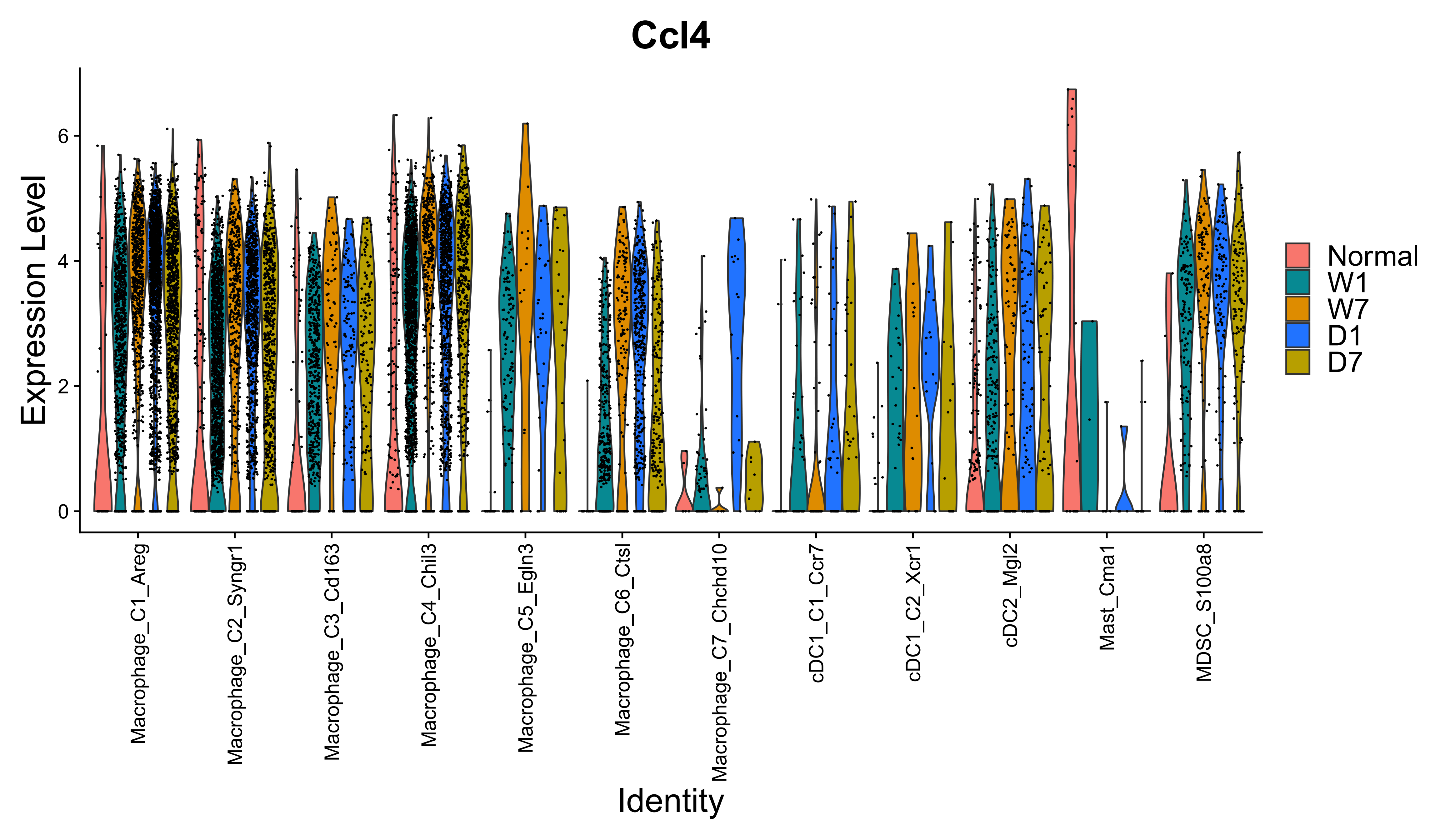

Ccl4
Expression Level
0
2
4
6
Macrophage_C1_Areg
Macrophage_C2_Syngr1
Macrophage_C3_Cd163
Macrophage_C4_Chil3
Macrophage_C5_Egln3
Macrophage_C6_Ctsl
Macrophage_C7_Chchd10
cDC1_C1_Ccr7
cDC1_C2_Xcr1
cDC2_Mgl2
Mast_Cma1
MDSC_S100a8
Identity
Normal
W1
W7
D1
D7

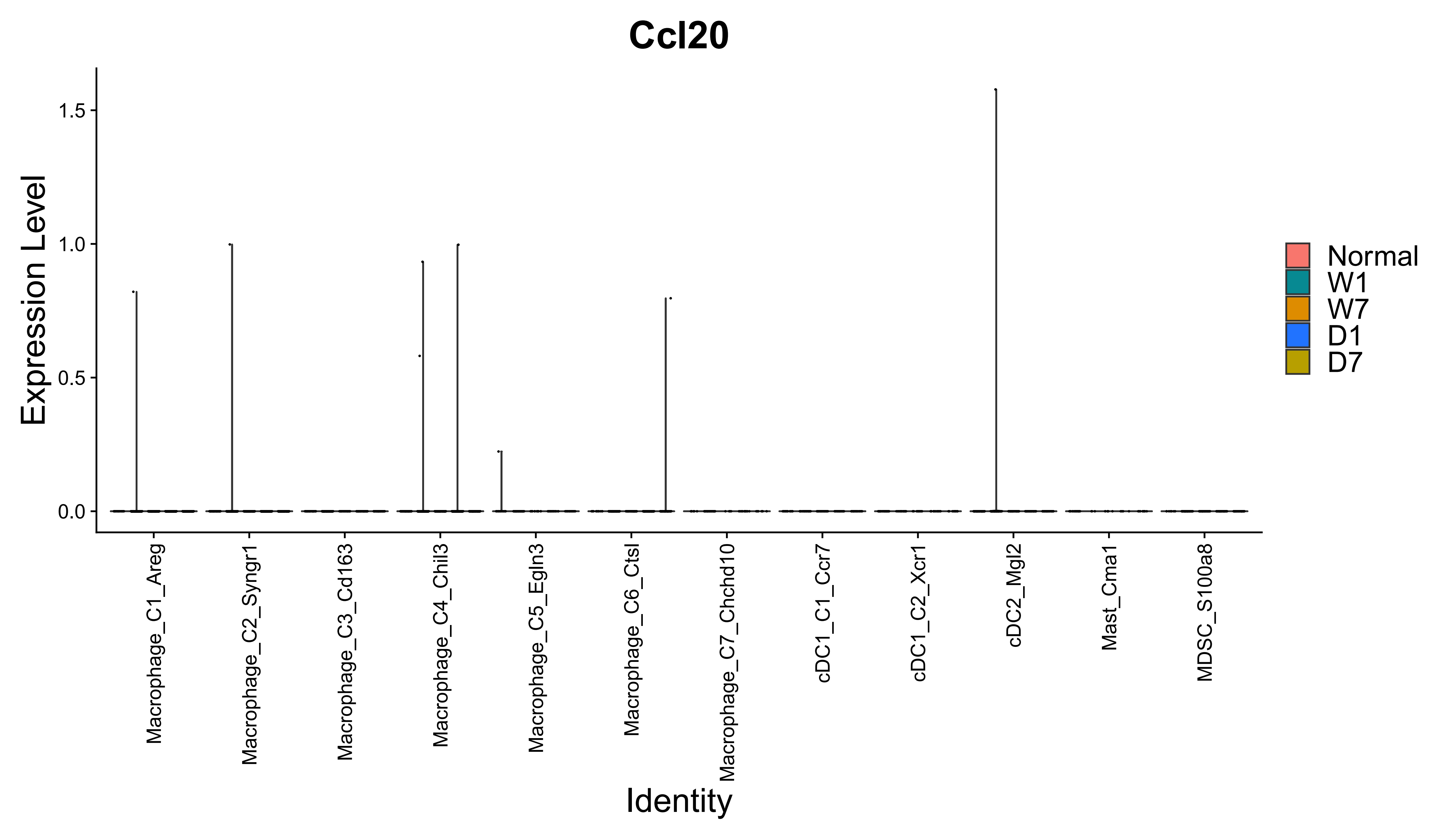

Ccl20
Expression Level
1.5
1.0
0.5
0.0
Macrophage_C1_Areg
Macrophage_C2_Syngr1
Macrophage_C3_Cd163
Macrophage_C4_Chil3
Macrophage_C5_Egln3
Macrophage_C6_Ctsl
Macrophage_C7_Chchd10
cDC1_C1_Ccr7
cDC1_C2_Xcr1
cDC2_Mgl2
Mast_Cma1
MDSC_S100a8
Identity
Normal
W1
W7
D1
D7

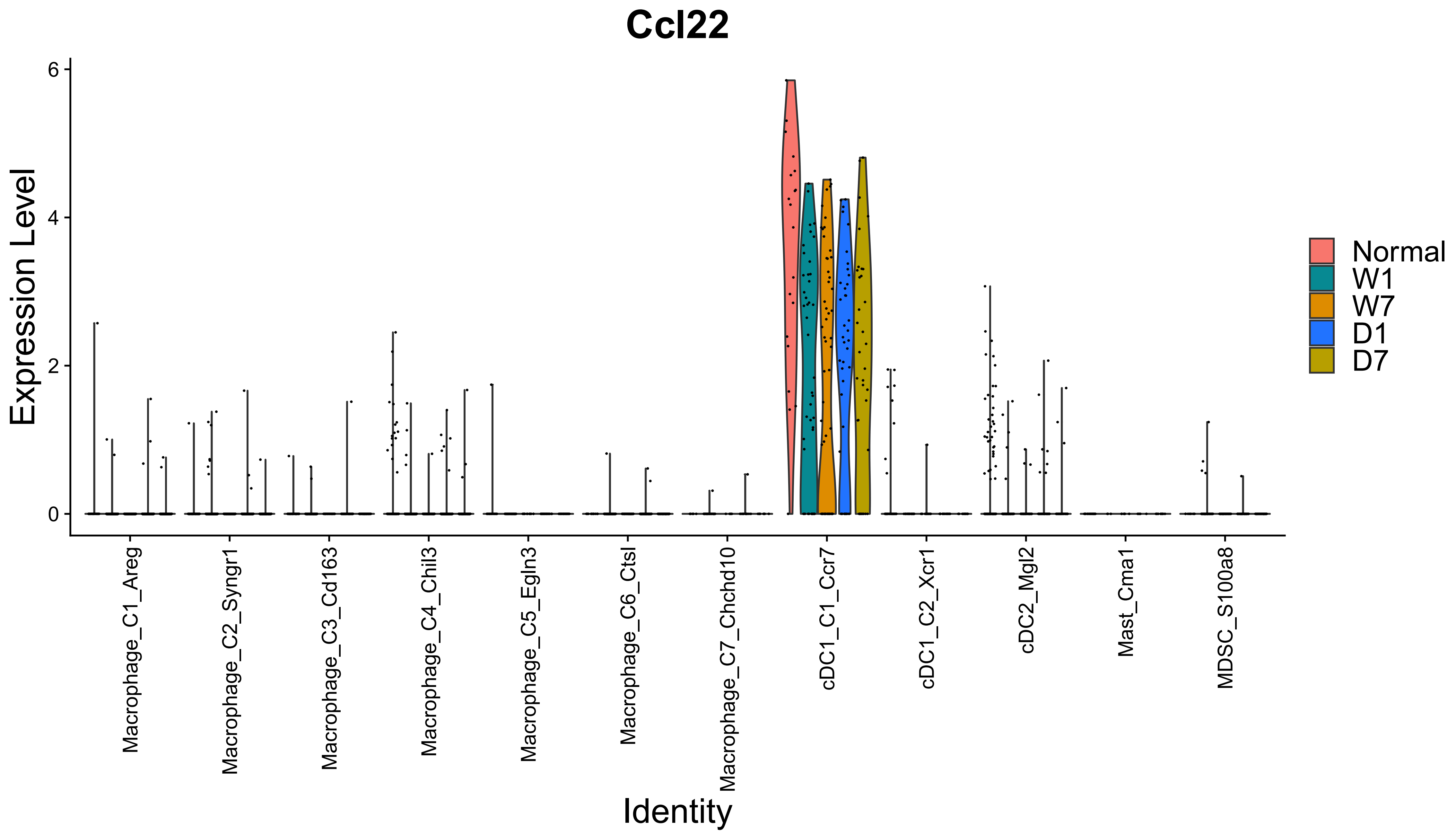

Ccl22
Expression Level
6
4
2
0
Normal
W1
W7
D1
D7
Macrophage_C1_Areg
Macrophage_C2_Syngr1
Macrophage_C3_Cd163
Macrophage_C4_Chil3
Macrophage_C5_Egln3
Macrophage_C6_Ctsl
Macrophage_C7_Chchd10
cDC1_C1_Ccr7
cDC1_C2_Xcr1
cDC2_Mgl2
Mast_Cma1
MDSC_S100a8
Identity

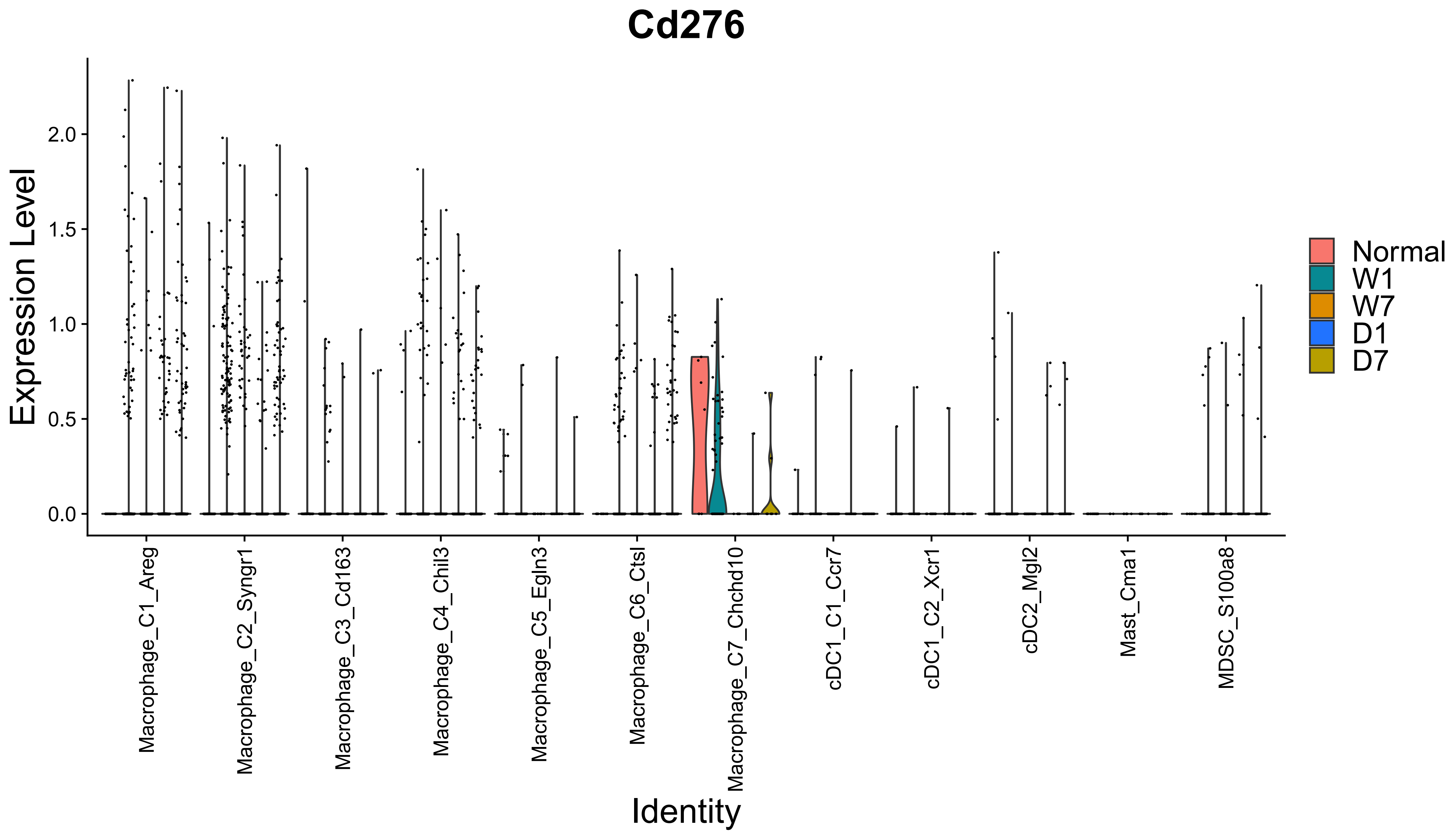
Cd276
Expression Level
2.0
1.5
1.0
0.5
0.0
Identity
Macrophage_C1_Areg
Macrophage_C2_Syngr1
Macrophage_C3_Cd163
Macrophage_C4_Chil3
Macrophage_C5_Egln3
Macrophage_C6_Ctsl
Macrophage_C7_Chchd10
cDC1_C1_Ccr7
cDC1_C2_Xcr1
cDC2_Mgl2
Mast_Cma1
MDSC_S100a8
Normal
W1
W7
D1
D7

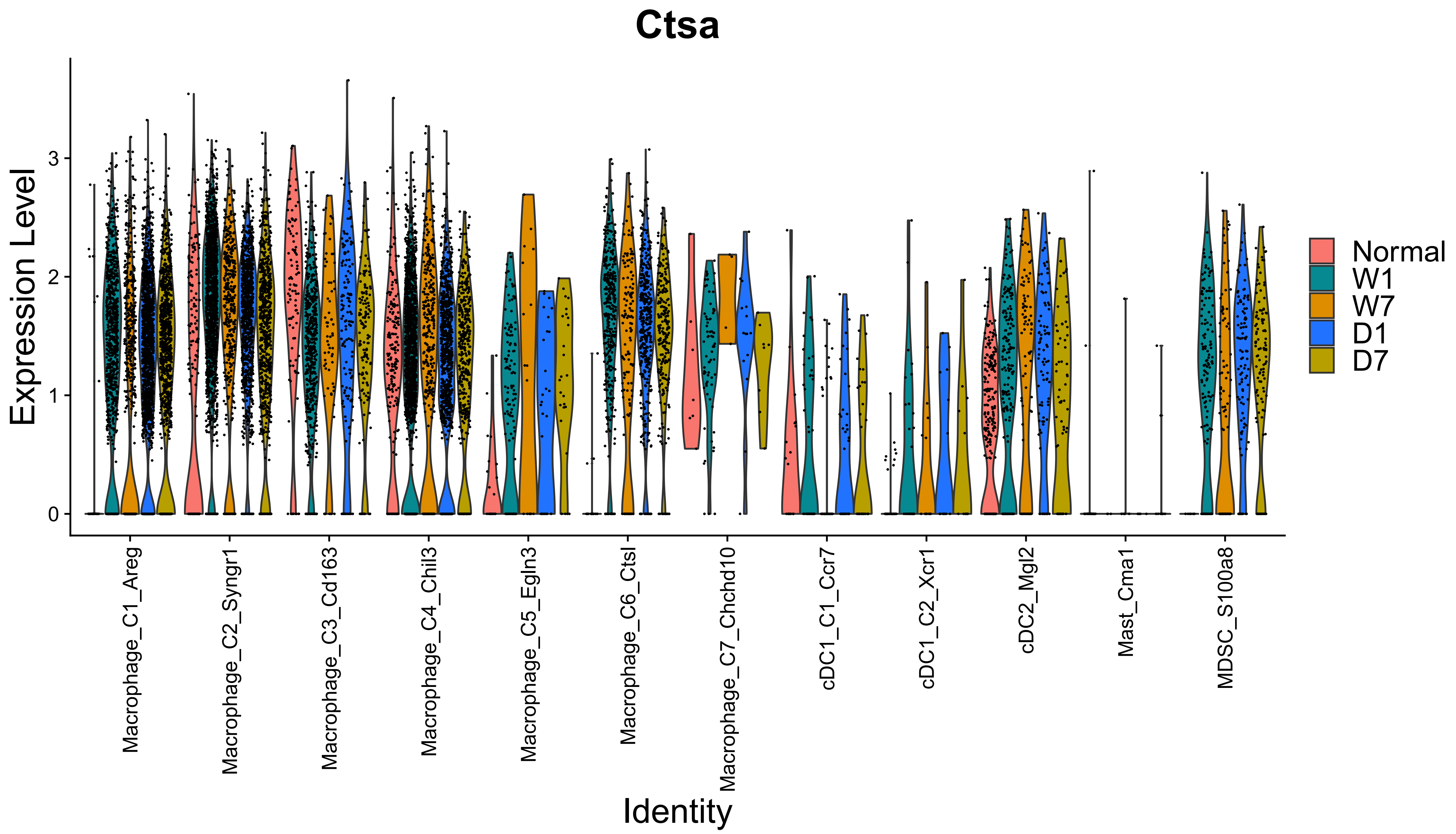

Ctsa
Expression Level
0
1
2
3
Normal
W1
W7
D1
D7
Macrophage_C1_Areg
Macrophage_C2_Syngr1
Macrophage_C3_Cd163
Macrophage_C4_Chil3
Macrophage_C5_Egln3
Macrophage_C6_Ctsl
Macrophage_C7_Chchd10
cDC1_C1_Ccr7
cDC1_C2_Xcr1
cDC2_Mgl2
Mast_Cma1
MDSC_S100a8
Identity

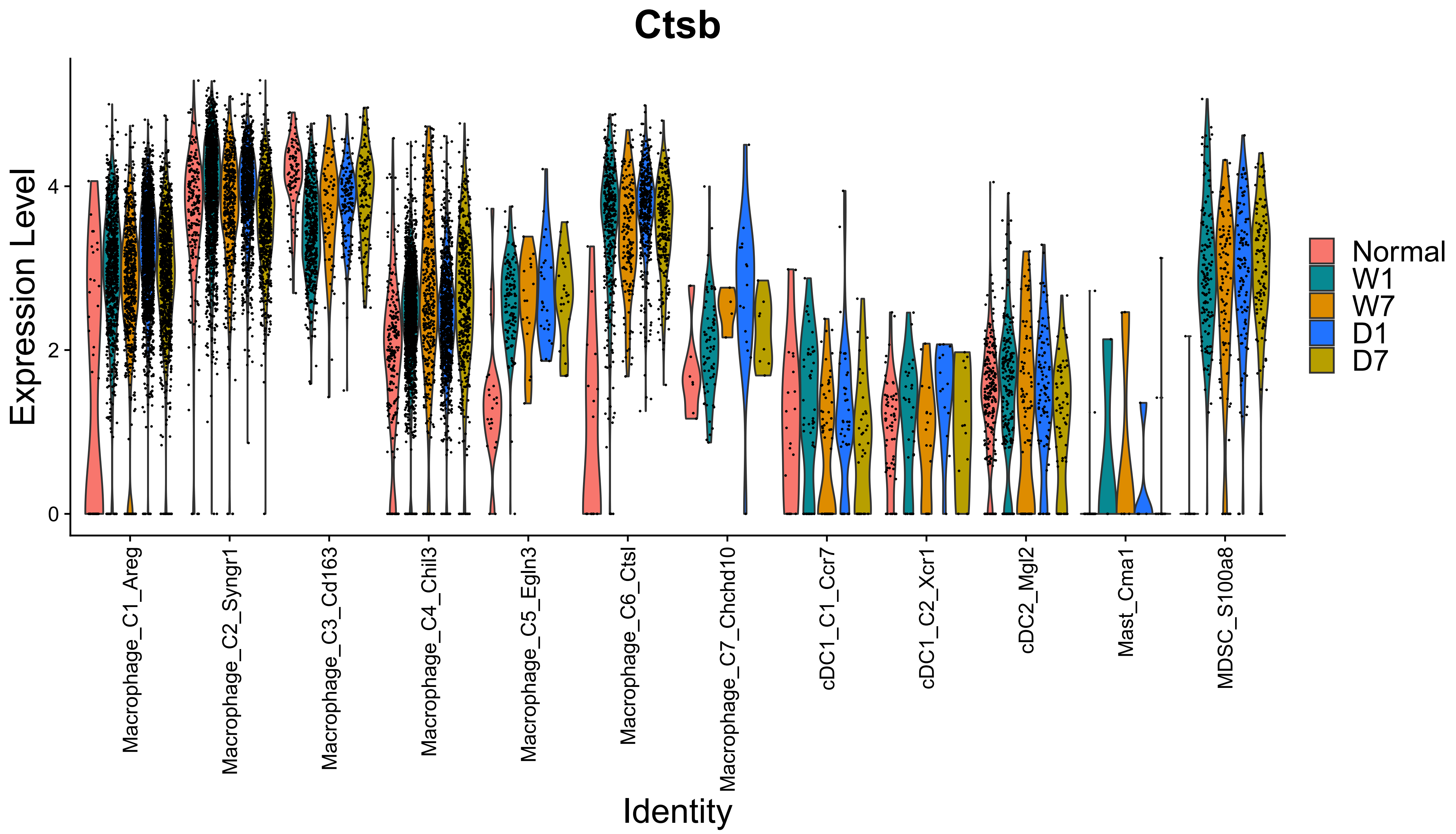

Ctsb
Expression Level
0
2
4
Normal
W1
W7
D1
D7
Macrophage_C1_Areg
Macrophage_C2_Syngr1
Macrophage_C3_Cd163
Macrophage_C4_Chil3
Macrophage_C5_Egln3
Macrophage_C6_Ctsl
Macrophage_C7_Chchd10
cDC1_C1_Ccr7
cDC1_C2_Xcr1
cDC2_Mgl2
Mast_Cma1
MDSC_S100a8
Identity

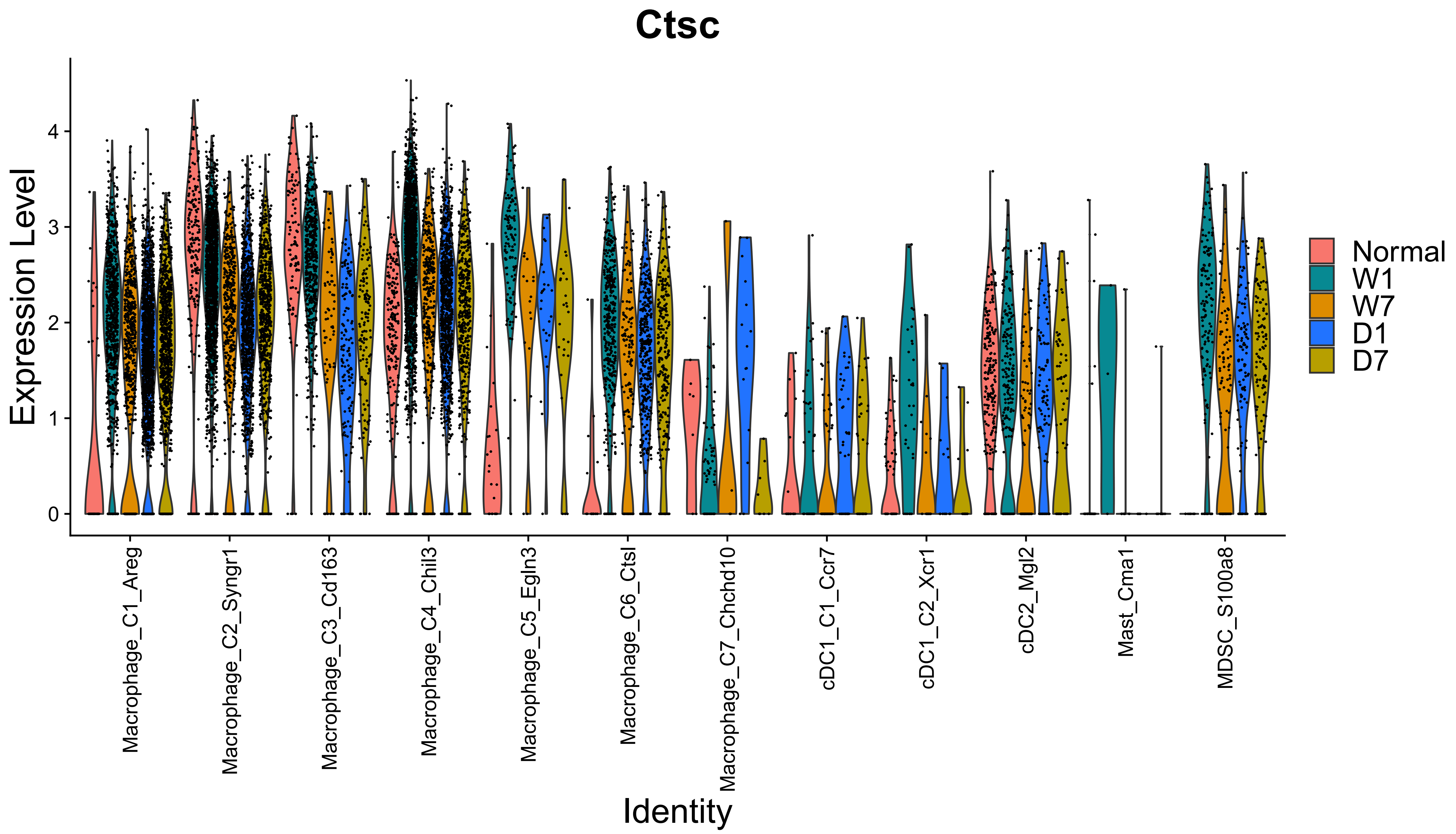

Ctsc
Expression Level
0
1
2
3
4
Identity
Macrophage_C1_Areg
Macrophage_C2_Syngr1
Macrophage_C3_Cd163
Macrophage_C4_Chil3
Macrophage_C5_Egln3
Macrophage_C6_Ctsl
Macrophage_C7_Chchd10
cDC1_C1_Ccr7
cDC1_C2_Xcr1
cDC2_Mgl2
Mast_Cma1
MDSC_S100a8
Normal
W1
W7
D1
D7

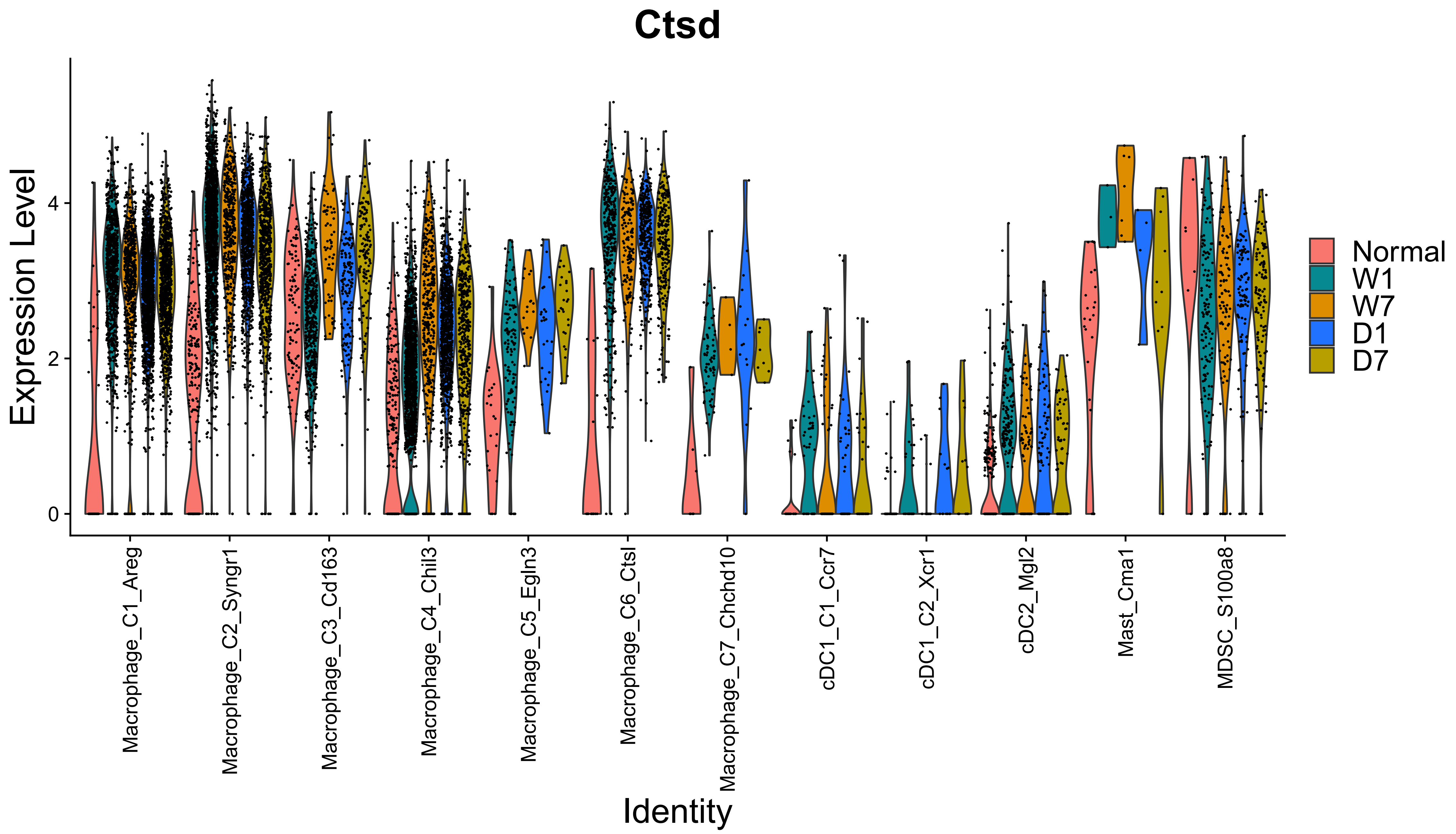
Ctsd
Expression Level
0
2
4
Identity
Macrophage_C1_Areg
Macrophage_C2_Syngr1
Macrophage_C3_Cd163
Macrophage_C4_Chil3
Macrophage_C5_Egln3
Macrophage_C6_Ctsl
Macrophage_C7_Chchd10
cDC1_C1_Ccr7
cDC1_C2_Xcr1
cDC2_Mgl2
Mast_Cma1
MDSC_S100a8
Normal
W1
W7
D1
D7

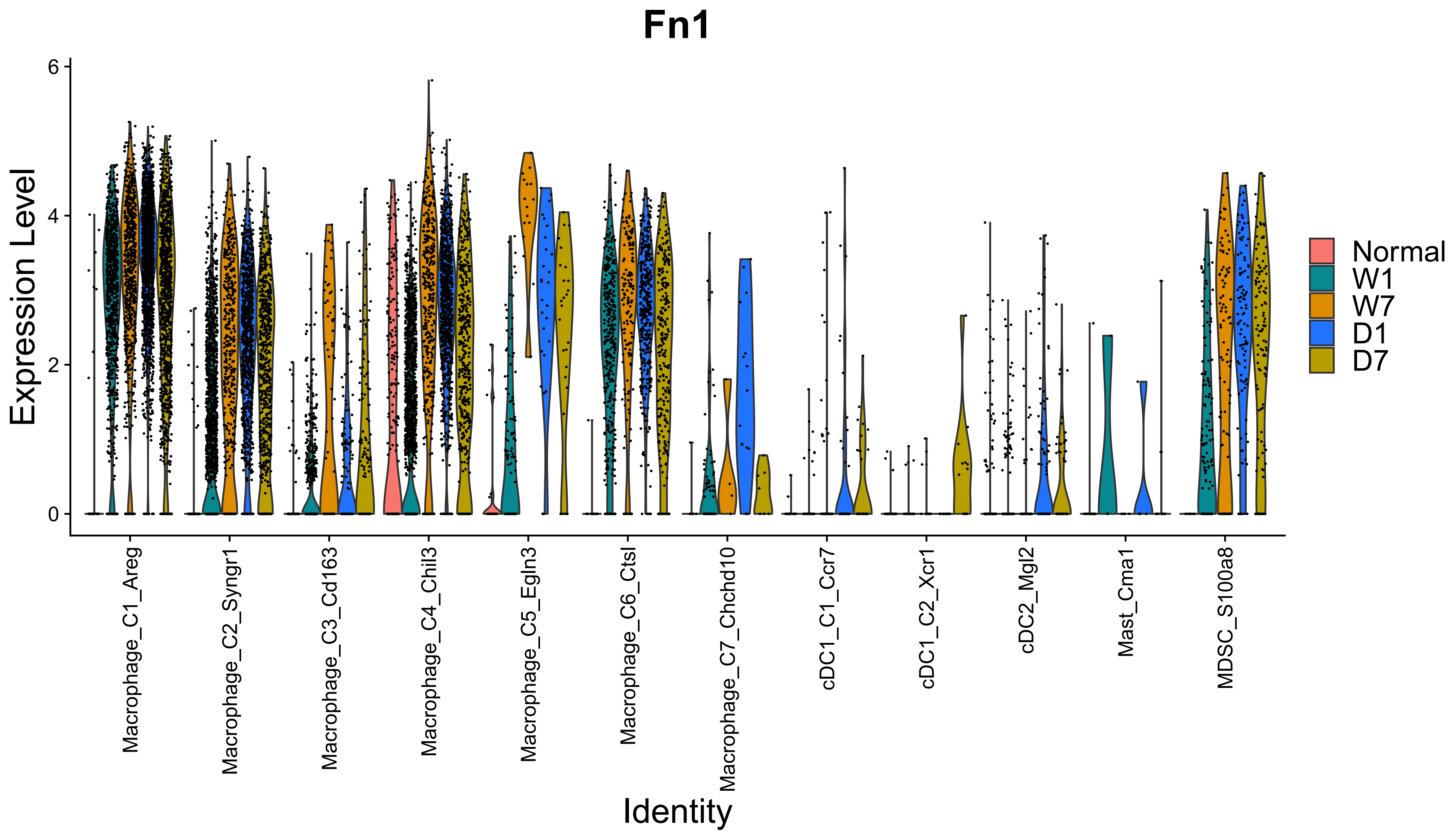
Fn1
Expression Level
0
2
4
6
Identity
Macrophage_C1_Areg
Macrophage_C2_Syngr1
Macrophage_C3_Cd163
Macrophage_C4_Chil3
Macrophage_C5_Egln3
Macrophage_C6_Ctsl
Macrophage_C7_Chchd10
cDC1_C1_Ccr7
cDC1_C2_Xcr1
cDC2_Mgl2
Mast_Cma1
MDSC_S100a8
Normal
W1
W7
D1
D7

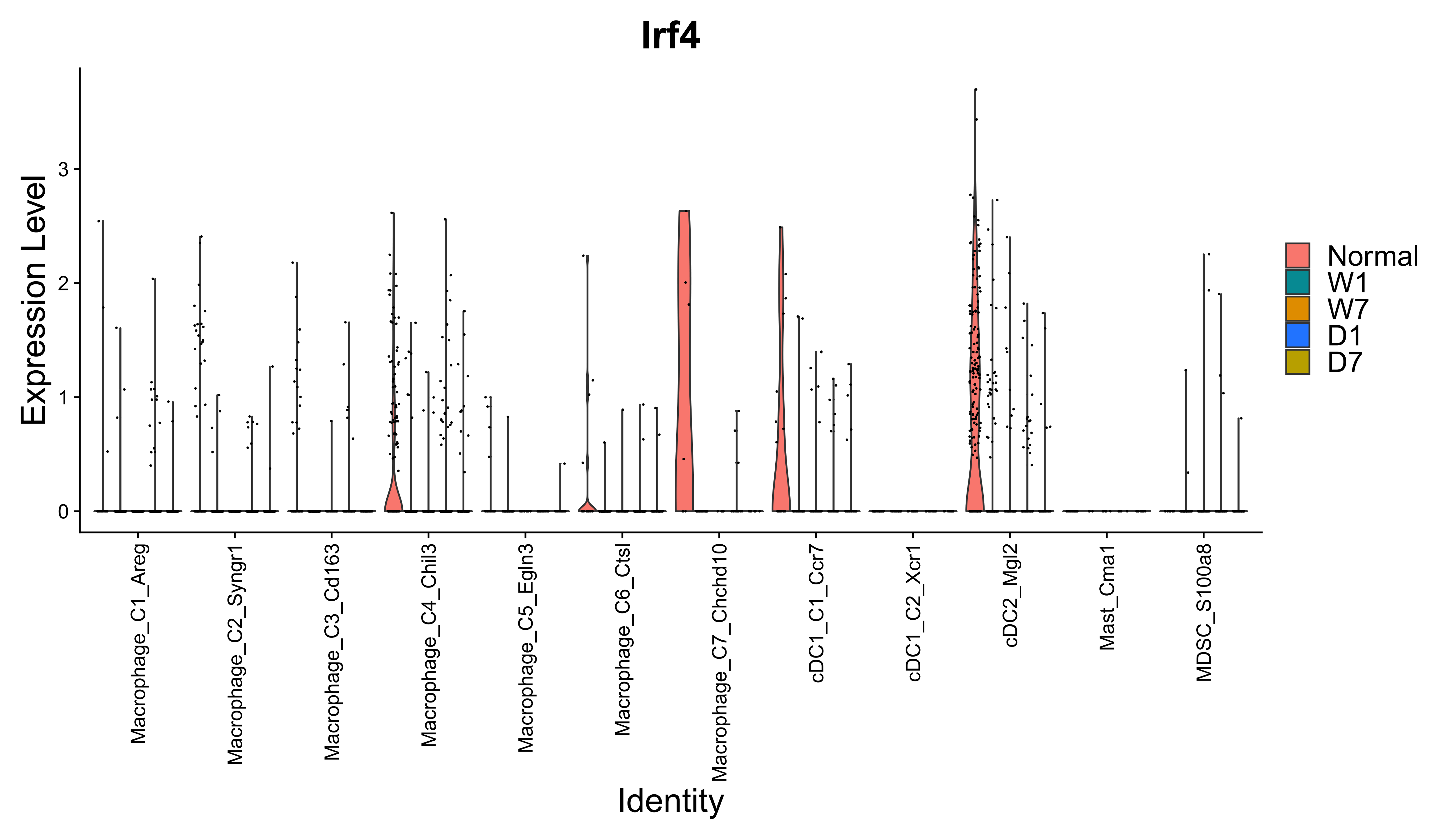

Irf4
Expression Level
0
1
2
3
Macrophage_C1_Areg
Macrophage_C2_Syngr1
Macrophage_C3_Cd163
Macrophage_C4_Chil3
Macrophage_C5_Egln3
Macrophage_C6_Ctsl
Macrophage_C7_Chchd10
cDC1_C1_Ccr7
cDC1_C2_Xcr1
cDC2_Mgl2
Mast_Cma1
MDSC_S100a8
Identity
Normal
W1
W7
D1
D7

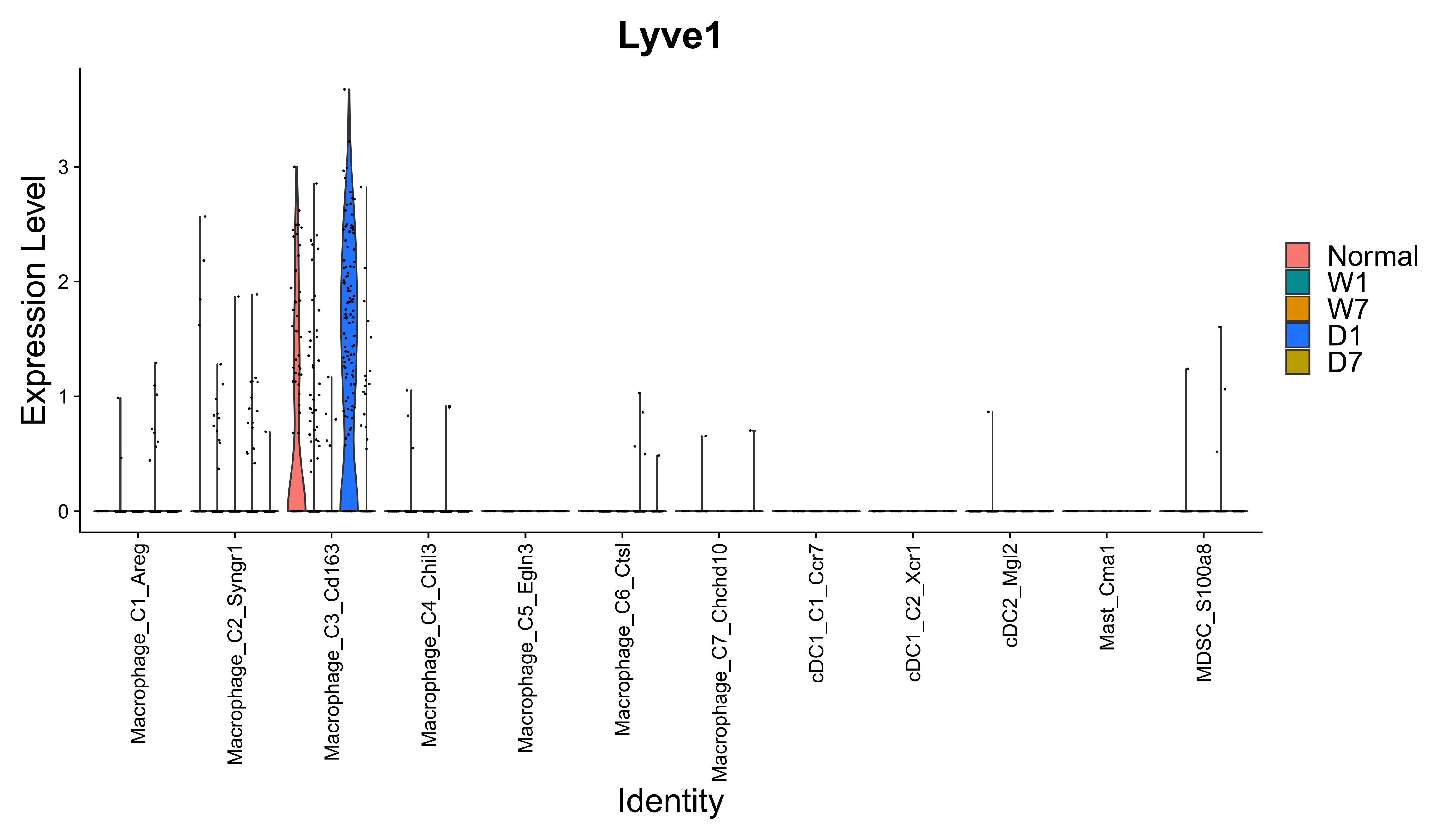

Lyve1
Expression Level
0
1
2
3
Identity
Macrophage_C1_Areg
Macrophage_C2_Syngr1
Macrophage_C3_Cd163
Macrophage_C4_Chil3
Macrophage_C5_Egln3
Macrophage_C6_Ctsl
Macrophage_C7_Chchd10
cDC1_C1_Ccr7
cDC1_C2_Xcr1
cDC2_Mgl2
Mast_Cma1
MDSC_S100a8
Normal
W1
W7
D1
D7

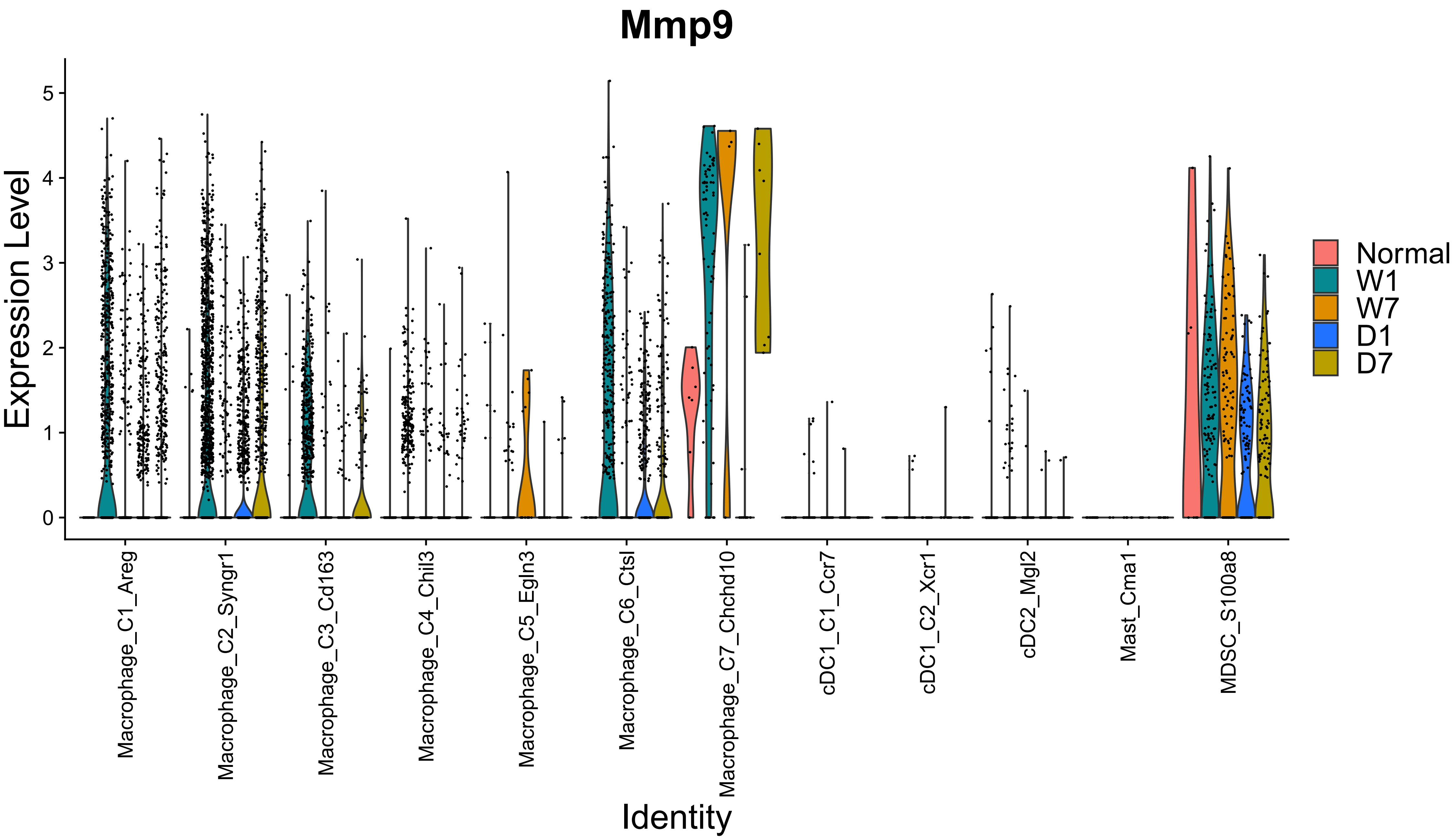

Mmp9
Expression Level
5
4
3
2
1
0
Identity
Macrophage_C1_Areg
Macrophage_C2_Syngr1
Macrophage_C3_Cd163
Macrophage_C4_Chil3
Macrophage_C5_Egln3
Macrophage_C6_Ctsl
Macrophage_C7_Chchd10
cDC1_C1_Ccr7
cDC1_C2_Xcr1
cDC2_Mgl2
Mast_Cma1
MDSC_S100a8
Normal
W1
W7
D1
D7

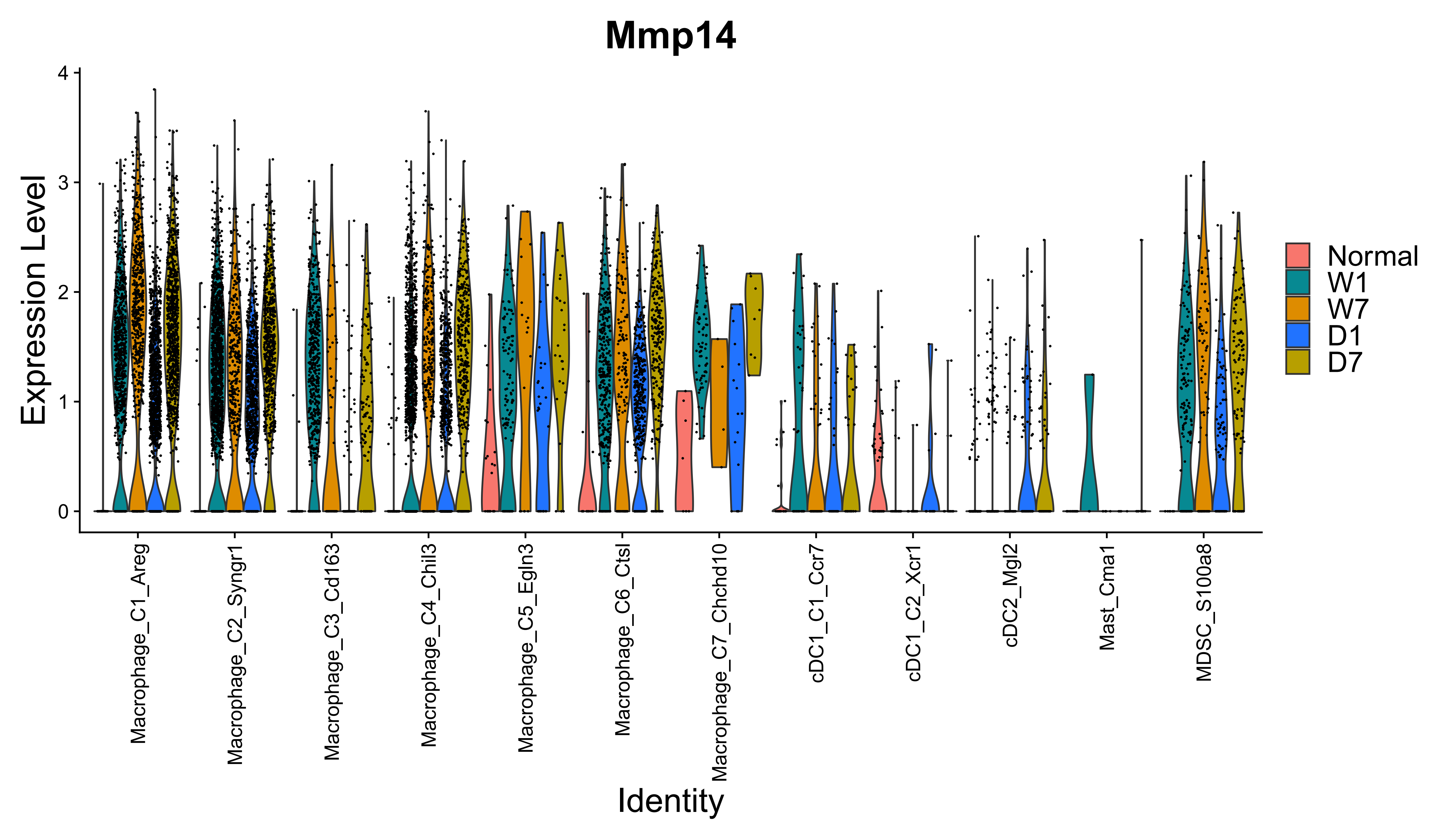

Mmp14
Expression Level
0
1
2
3
4
Identity
Macrophage_C1_Areg
Macrophage_C2_Syngr1
Macrophage_C3_Cd163
Macrophage_C4_Chil3
Macrophage_C5_Egln3
Macrophage_C6_Ctsl
Macrophage_C7_Chchd10
cDC1_C1_Ccr7
cDC1_C2_Xcr1
cDC2_Mgl2
Mast_Cma1
MDSC_S100a8
Normal
W1
W7
D1
D7

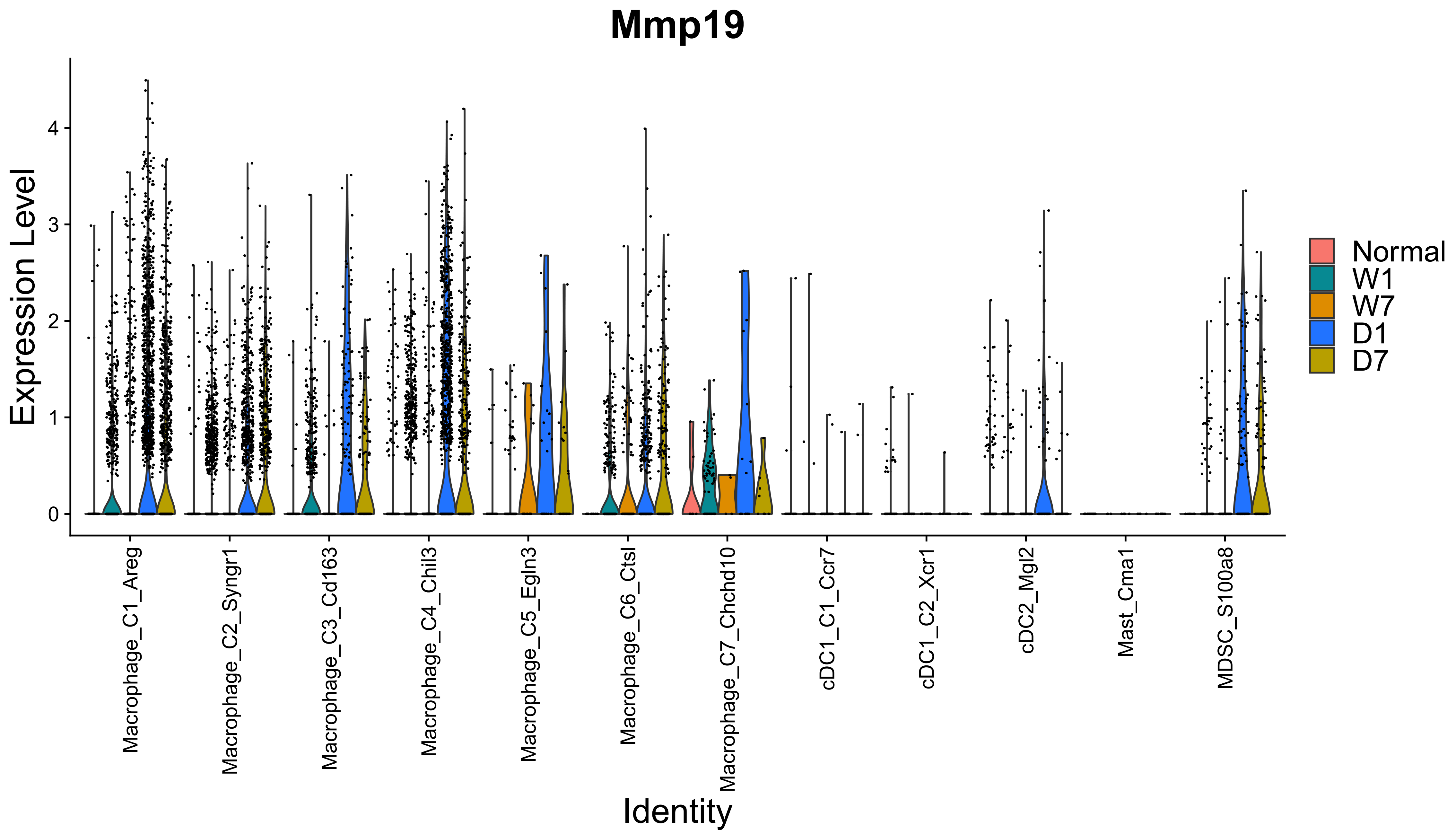

Mmp19
Expression Level
0
1
2
3
4
Identity
Macrophage_C1_Areg
Macrophage_C2_Syngr1
Macrophage_C3_Cd163
Macrophage_C4_Chil3
Macrophage_C5_Egln3
Macrophage_C6_Ctsl
Macrophage_C7_Chchd10
cDC1_C1_Ccr7
cDC1_C2_Xcr1
cDC2_Mgl2
Mast_Cma1
MDSC_S100a8
Normal
W1
W7
D1
D7

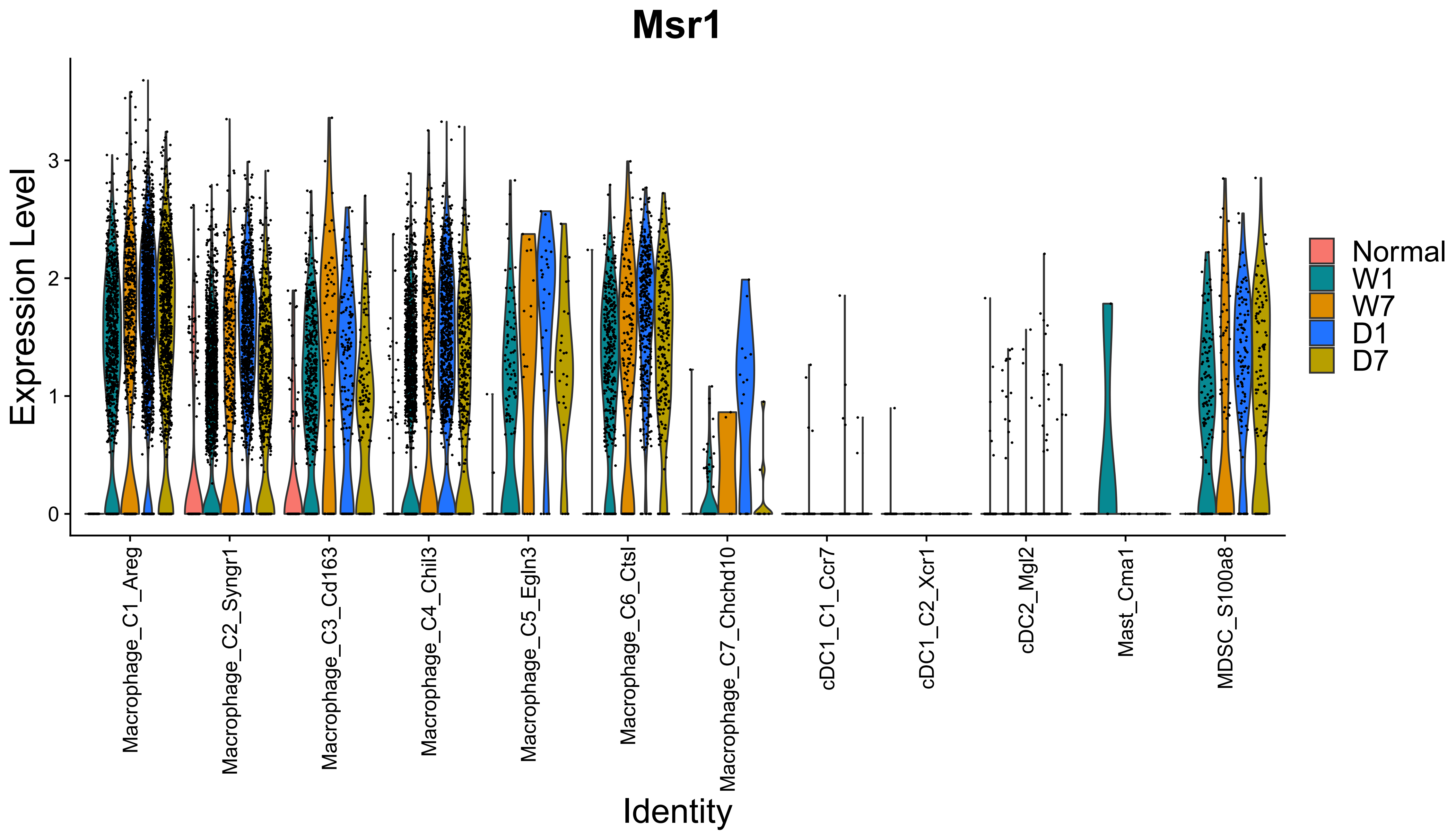

Msr1
Expression Level
0
1
2
3
Identity
Macrophage_C1_Areg
Macrophage_C2_Syngr1
Macrophage_C3_Cd163
Macrophage_C4_Chil3
Macrophage_C5_Egln3
Macrophage_C6_Ctsl
Macrophage_C7_Chchd10
cDC1_C1_Ccr7
cDC1_C2_Xcr1
cDC2_Mgl2
Mast_Cma1
MDSC_S100a8
Normal
W1
W7
D1
D7

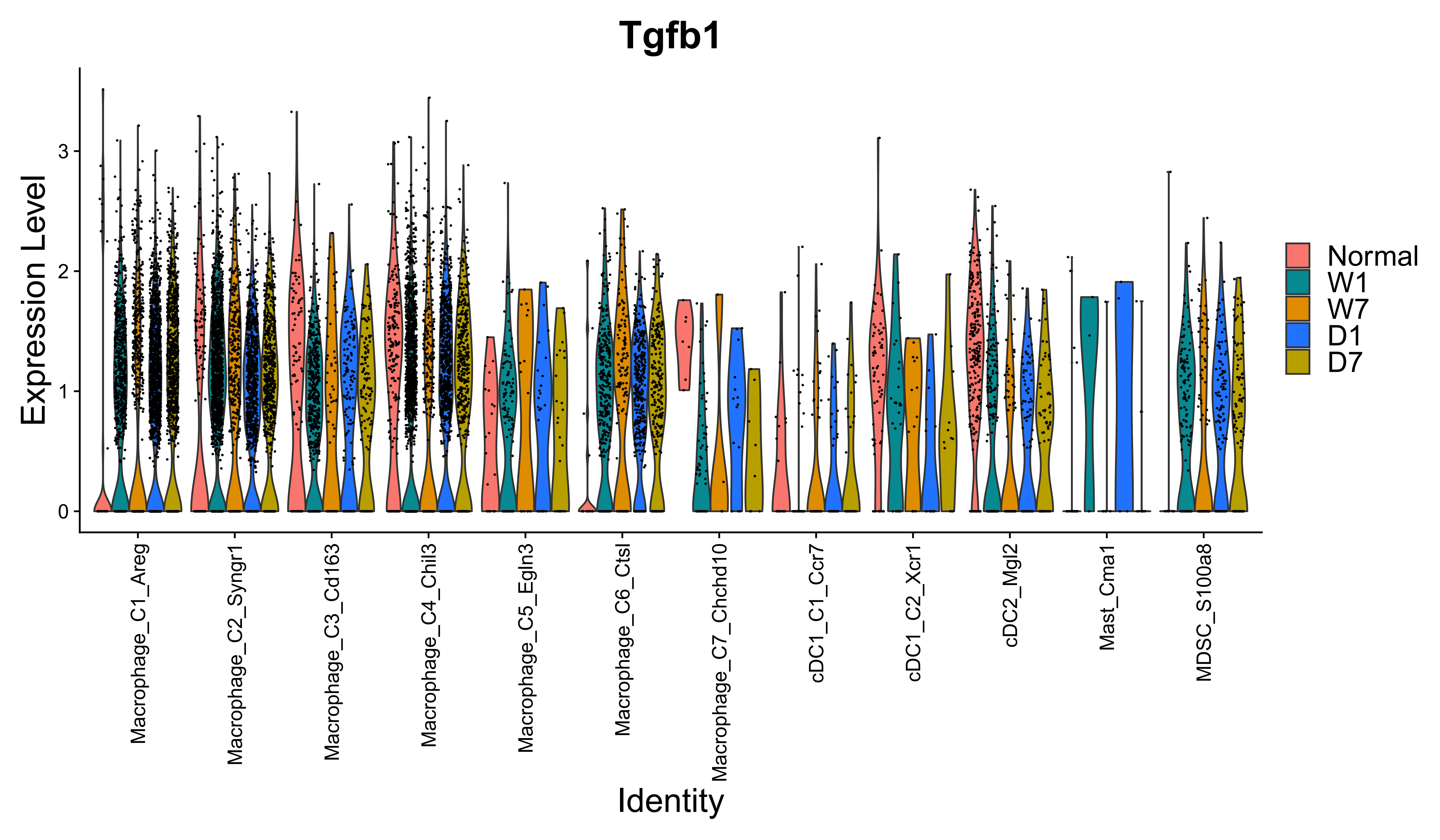
Tgfb1
Expression Level
0
1
2
3
Normal
W1
W7
D1
D7
Macrophage_C1_Areg
Macrophage_C2_Syngr1
Macrophage_C3_Cd163
Macrophage_C4_Chil3
Macrophage_C5_Egln3
Macrophage_C6_Ctsl
Macrophage_C7_Chchd10
cDC1_C1_Ccr7
cDC1_C2_Xcr1
cDC2_Mgl2
Mast_Cma1
MDSC_S100a8
Identity

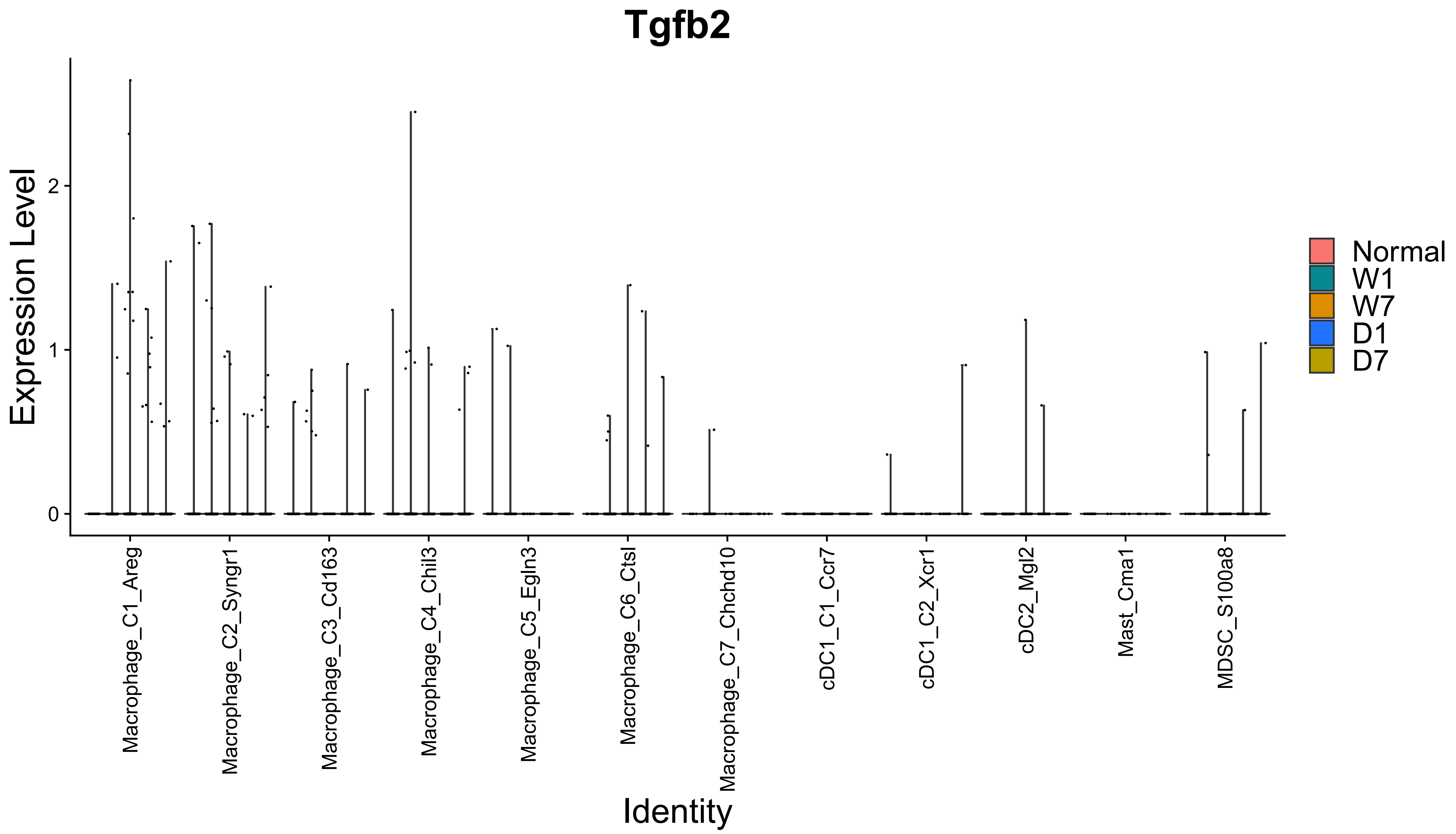

Tgfb2
Expression Level
0
1
2
Identity
Macrophage_C1_Areg
Macrophage_C2_Syngr1
Macrophage_C3_Cd163
Macrophage_C4_Chil3
Macrophage_C5_Egln3
Macrophage_C6_Ctsl
Macrophage_C7_Chchd10
cDC1_C1_Ccr7
cDC1_C2_Xcr1
cDC2_Mgl2
Mast_Cma1
MDSC_S100a8
Normal
W1
W7
D1
D7

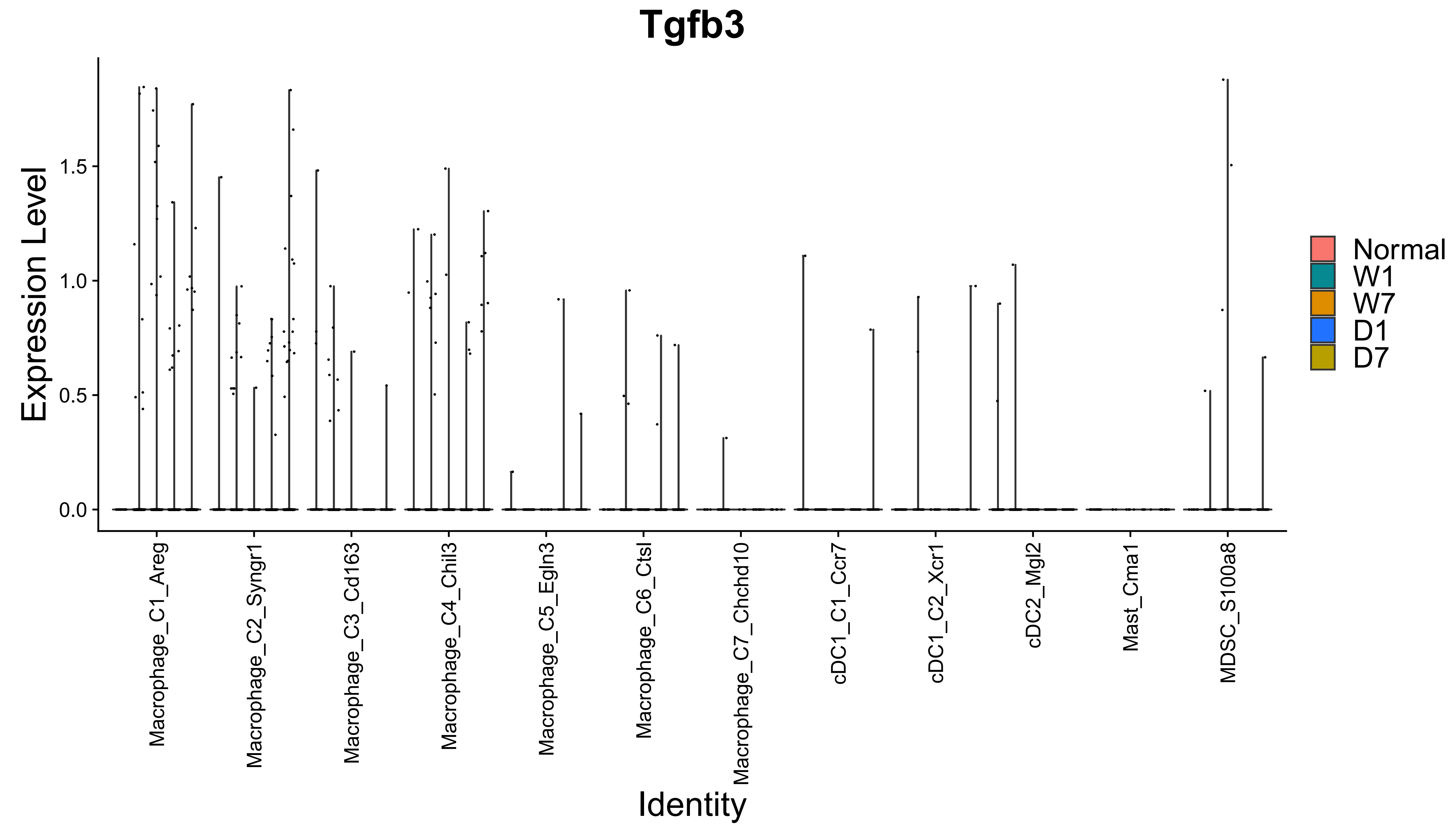

Tgfb3
Expression Level
1.5
1.0
0.5
0.0
Macrophage_C1_Areg
Macrophage_C2_Syngr1
Macrophage_C3_Cd163
Macrophage_C4_Chil3
Macrophage_C5_Egln3
Macrophage_C6_Ctsl
Macrophage_C7_Chchd10
cDC1_C1_Ccr7
cDC1_C2_Xcr1
cDC2_Mgl2
Mast_Cma1
MDSC_S100a8
Identity
Normal
W1
W7
D1
D7

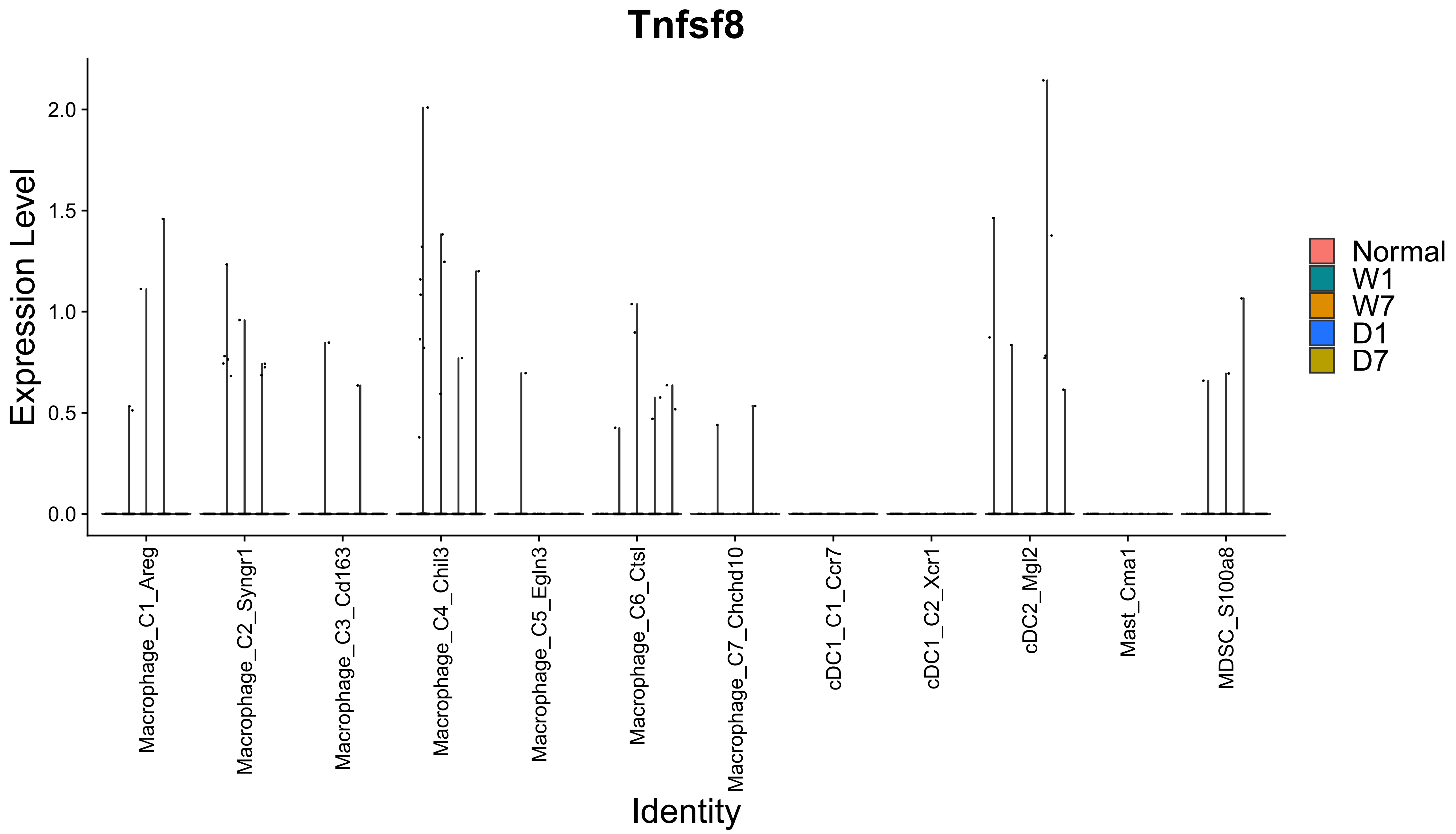

Tnfsf8
Expression Level
2.0
1.5
1.0
0.5
0.0
Macrophage_C1_Areg
Macrophage_C2_Syngr1
Macrophage_C3_Cd163
Macrophage_C4_Chil3
Macrophage_C5_Egln3
Macrophage_C6_Ctsl
Macrophage_C7_Chchd10
cDC1_C1_Ccr7
cDC1_C2_Xcr1
cDC2_Mgl2
Mast_Cma1
MDSC_S100a8
Identity
Normal
W1
W7
D1
D7

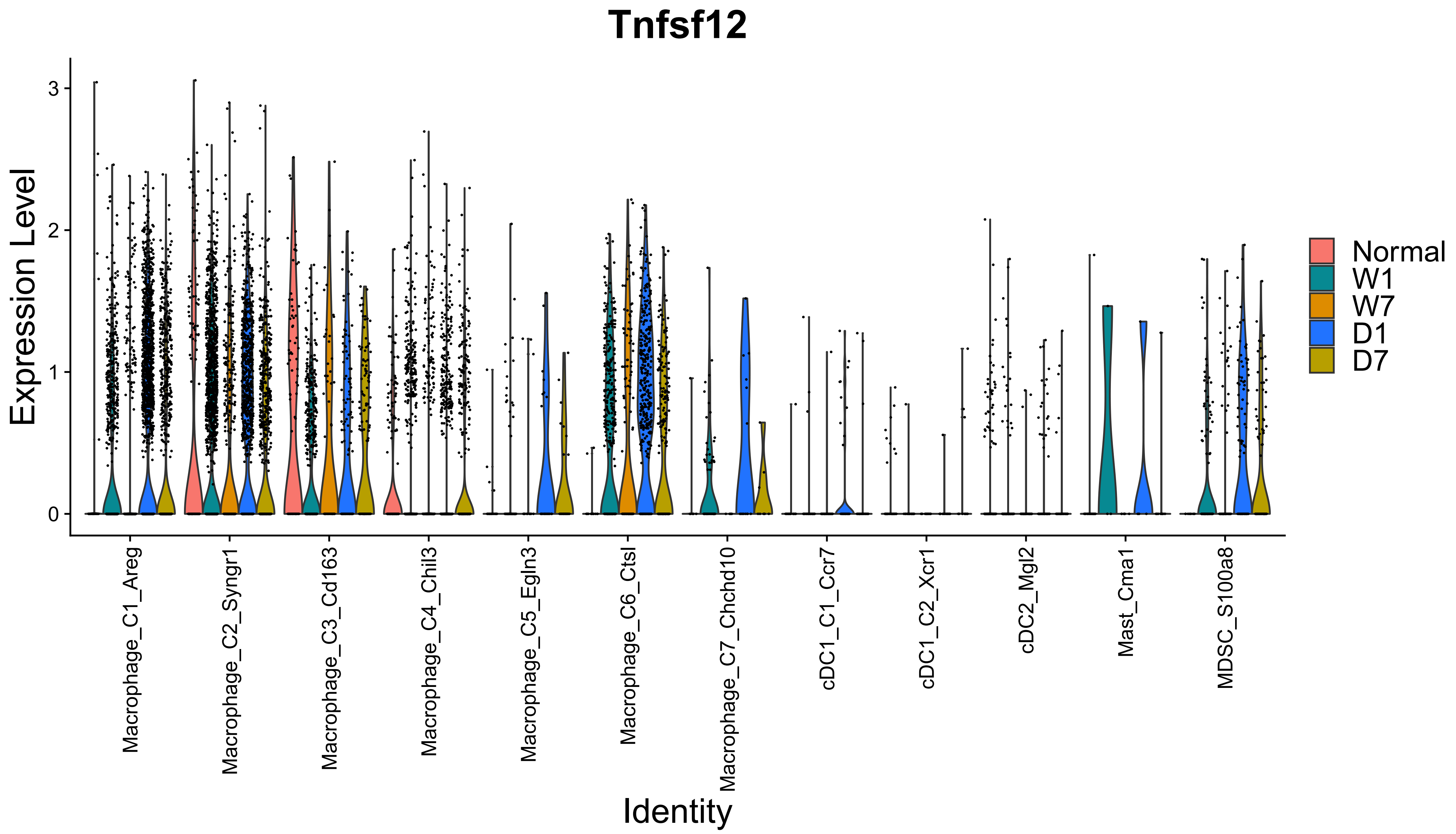
Tnfsf12
Expression Level
3
2
1
0
Identity
Macrophage_C1_Areg
Macrophage_C2_Syngr1
Macrophage_C3_Cd163
Macrophage_C4_Chil3
Macrophage_C5_Egln3
Macrophage_C6_Ctsl
Macrophage_C7_Chchd10
cDC1_C1_Ccr7
cDC1_C2_Xcr1
cDC2_Mgl2
Mast_Cma1
MDSC_S100a8
Normal
W1
W7
D1
D7

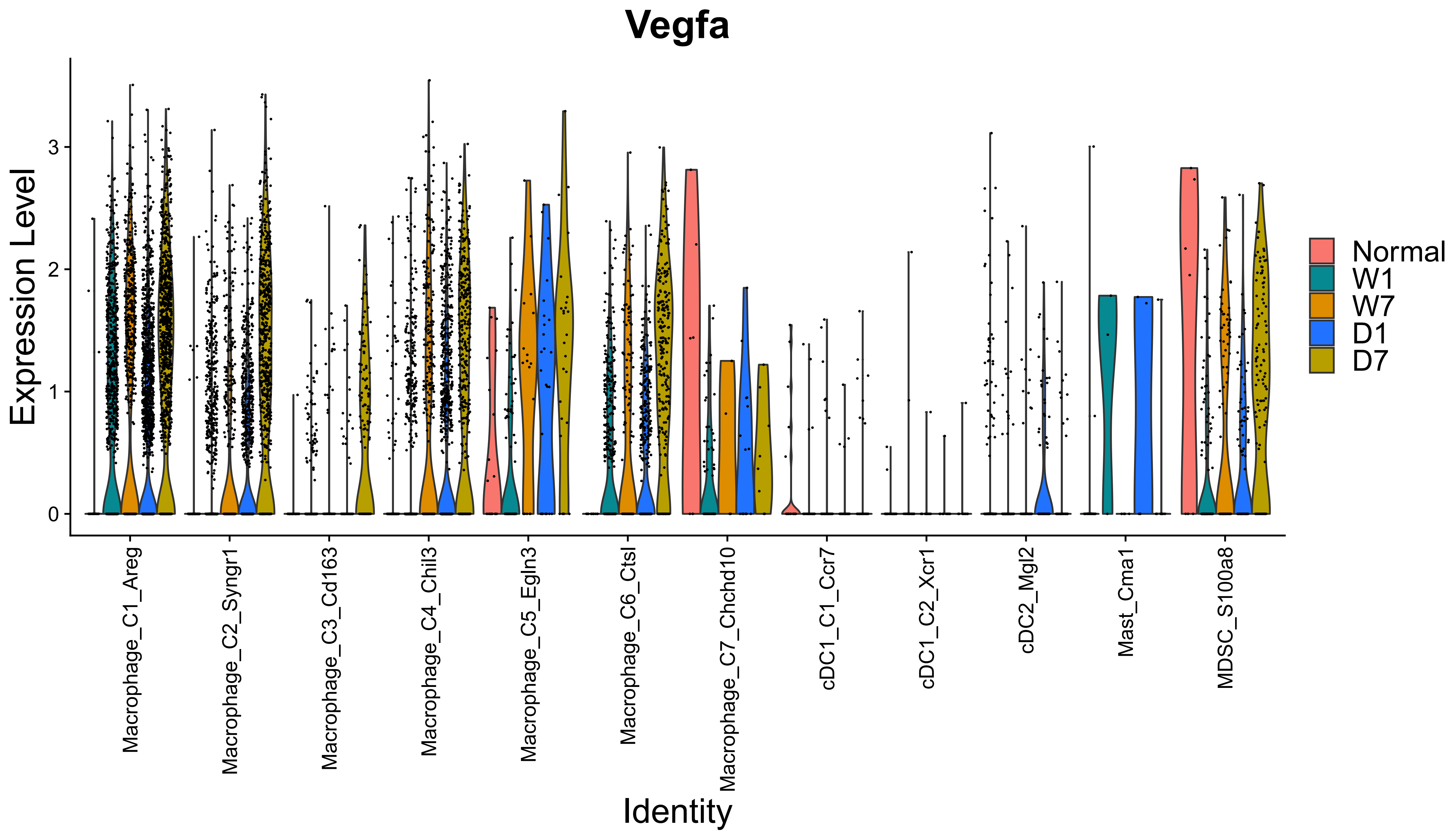

Vegfa
Expression Level
0
1
2
3
Macrophage_C1_Areg
Macrophage_C2_Syngr1
Macrophage_C3_Cd163
Macrophage_C4_Chil3
Macrophage_C5_Egln3
Macrophage_C6_Ctsl
Macrophage_C7_Chchd10
cDC1_C1_Ccr7
cDC1_C2_Xcr1
cDC2_Mgl2
Mast_Cma1
MDSC_S100a8
Identity
Normal
W1
W7
D1
D7

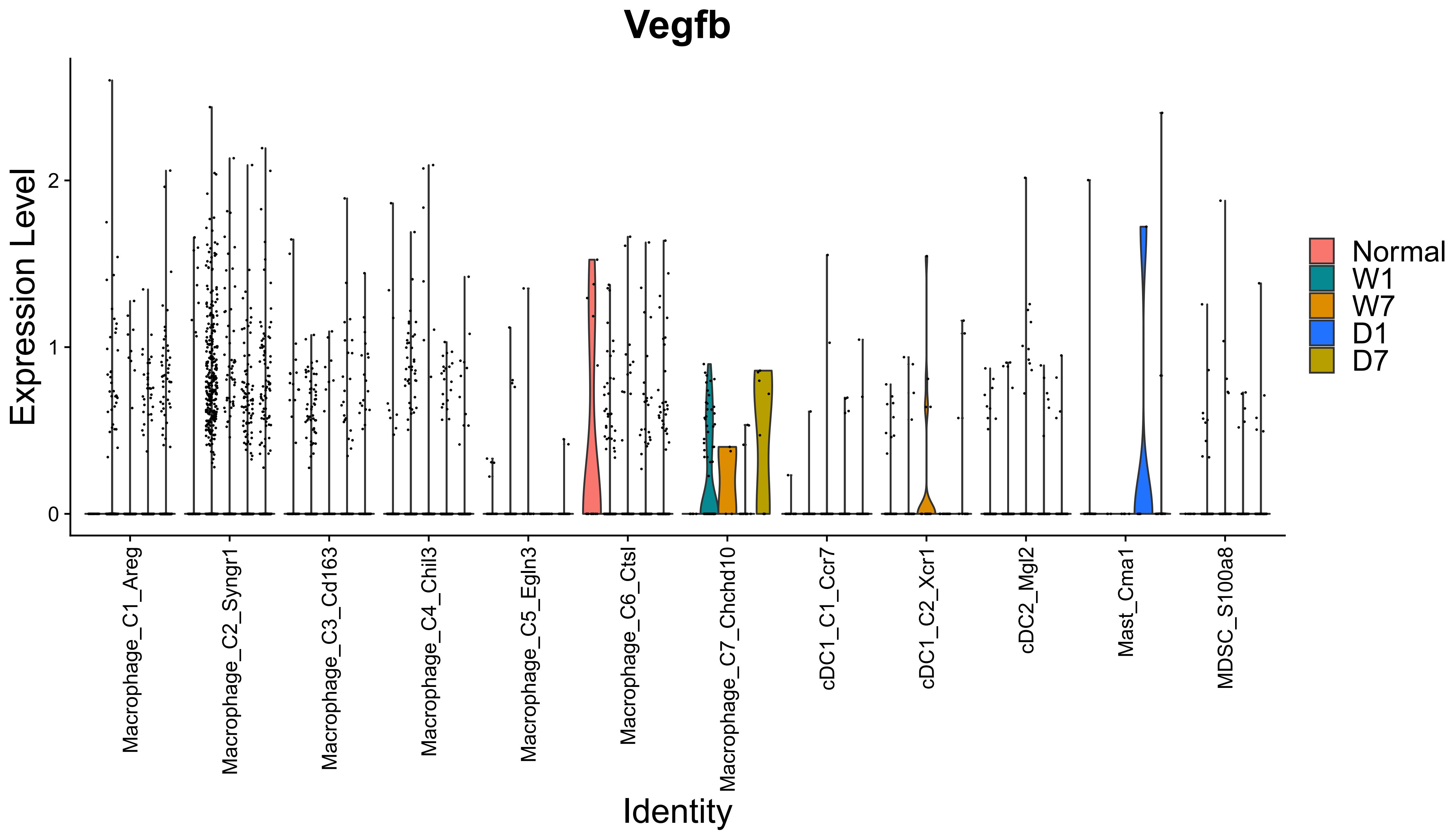
Vegfb
Expression Level
0
1
2
Identity
Macrophage_C1_Areg
Macrophage_C2_Syngr1
Macrophage_C3_Cd163
Macrophage_C4_Chil3
Macrophage_C5_Egln3
Macrophage_C6_Ctsl
Macrophage_C7_Chchd10
cDC1_C1_Ccr7
cDC1_C2_Xcr1
cDC2_Mgl2
Mast_Cma1
MDSC_S100a8
Normal
W1
W7
D1
D7

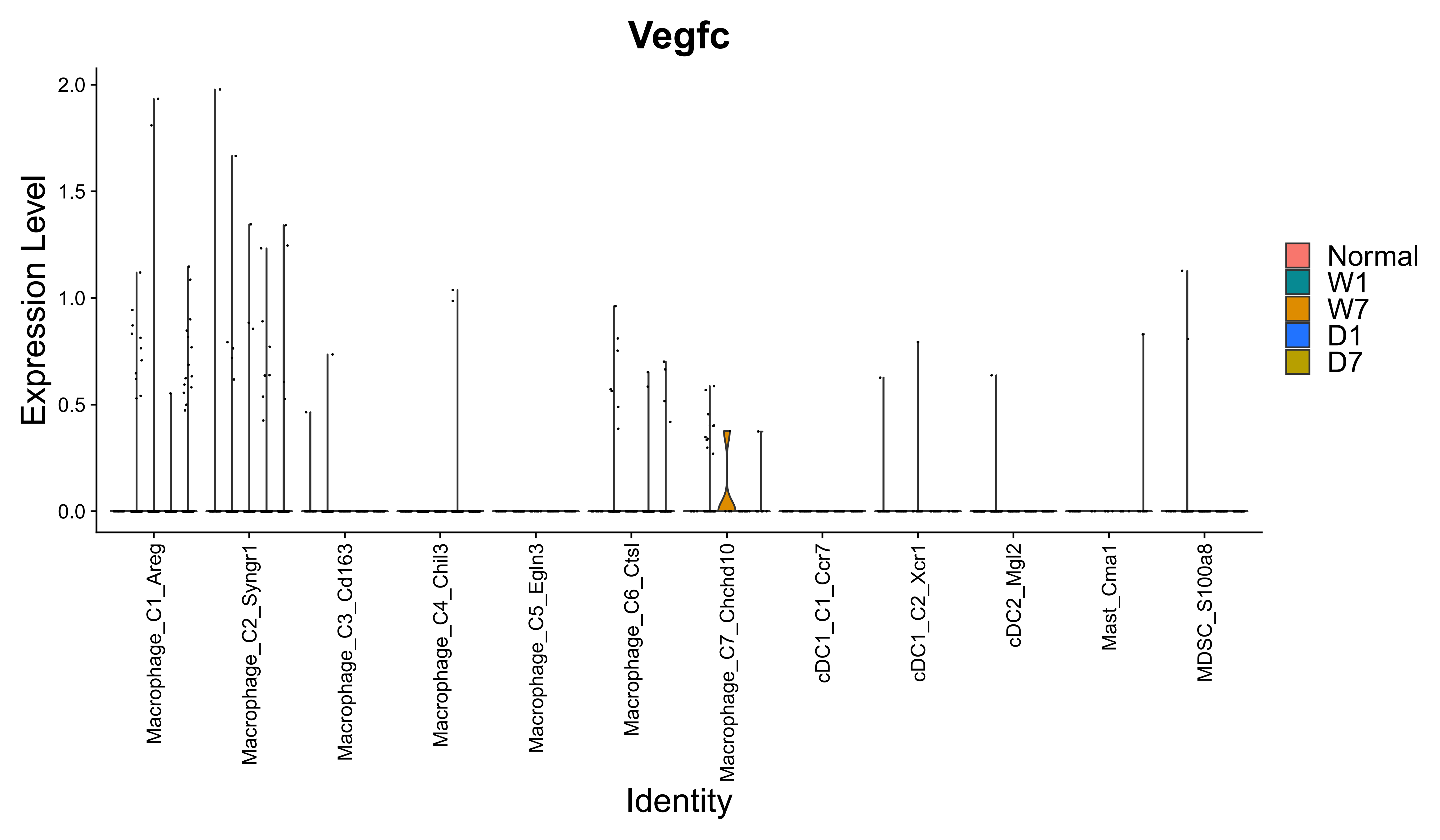

Vegfc
Expression Level
2.0
1.5
1.0
0.5
0.0
Macrophage_C1_Areg
Macrophage_C2_Syngr1
Macrophage_C3_Cd163
Macrophage_C4_Chil3
Macrophage_C5_Egln3
Macrophage_C6_Ctsl
Macrophage_C7_Chchd10
cDC1_C1_Ccr7
cDC1_C2_Xcr1
cDC2_Mgl2
Mast_Cma1
MDSC_S100a8
Identity
Normal
W1
W7
D1
D7
