# Supplementary material for: Single-cell RNA sequencing reveals that Danggui Buxue Tang decoction facilitates wound healing after anal fistula by promoting M2 macrophage polarization
Source: Hereditas. 2025 Oct 9;162:204. doi: 10.1186/s41065-025-00578-2 (PMC12512633; doi:10.1186/s41065-025-00578-2)

p-PI3K

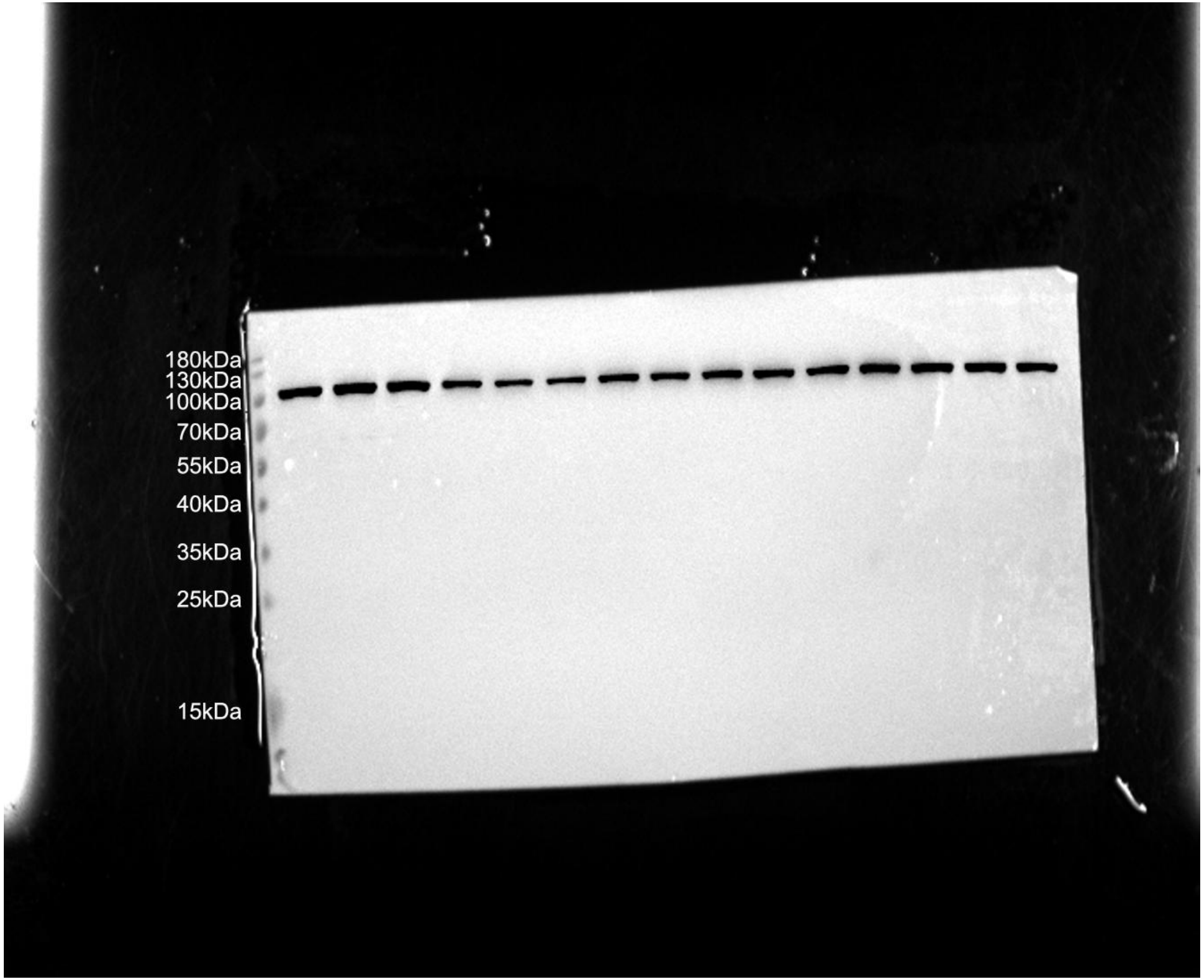

PI3K

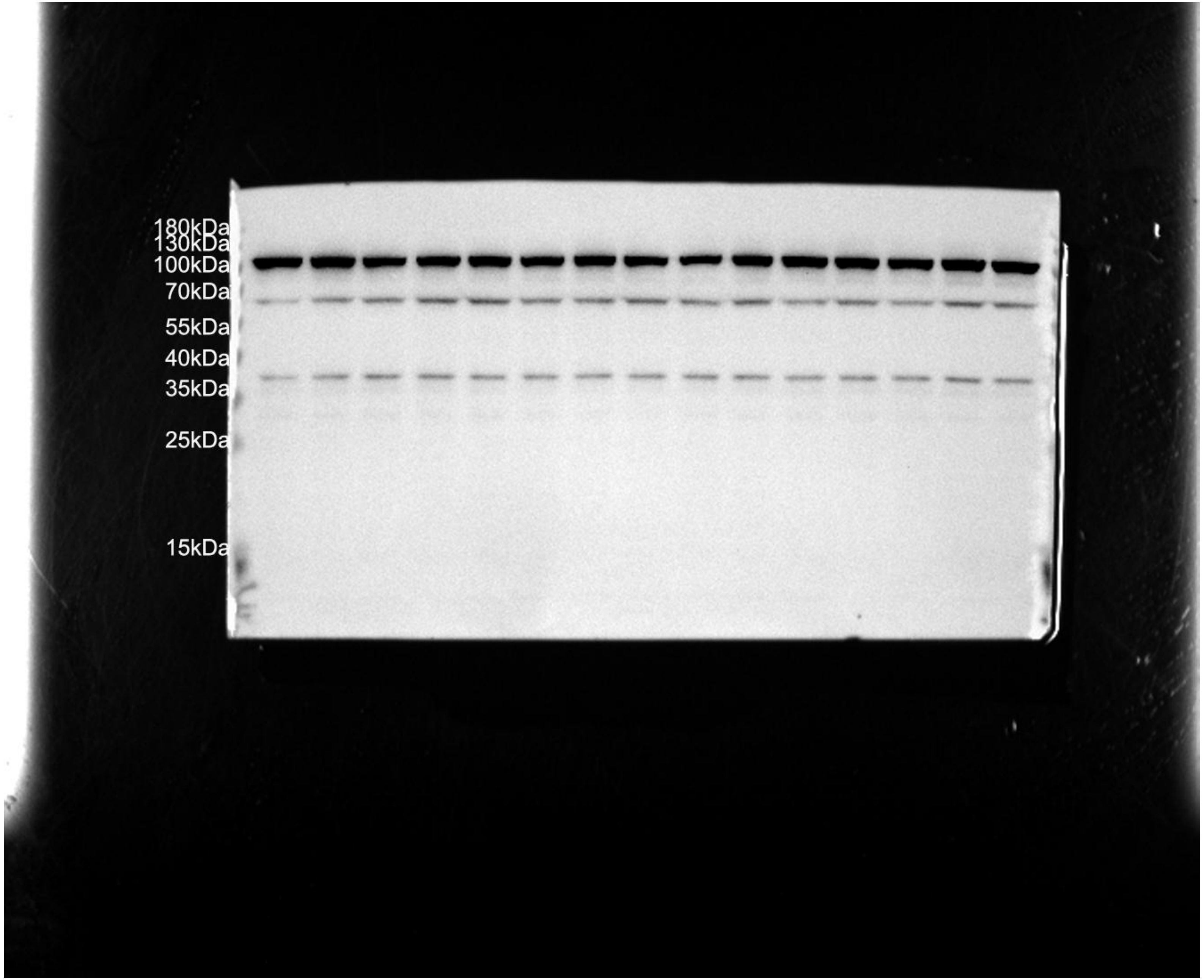

p-AKT

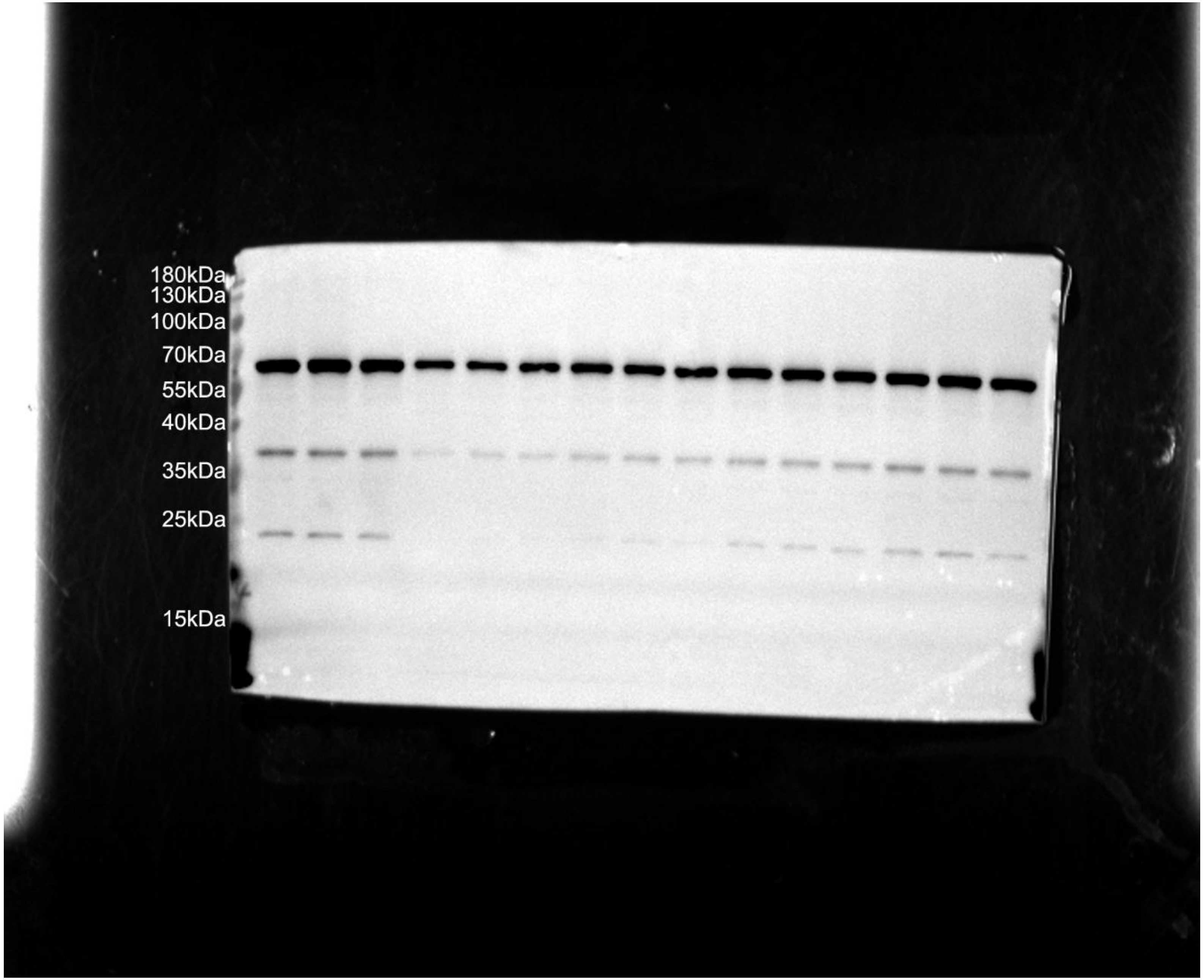

AKT

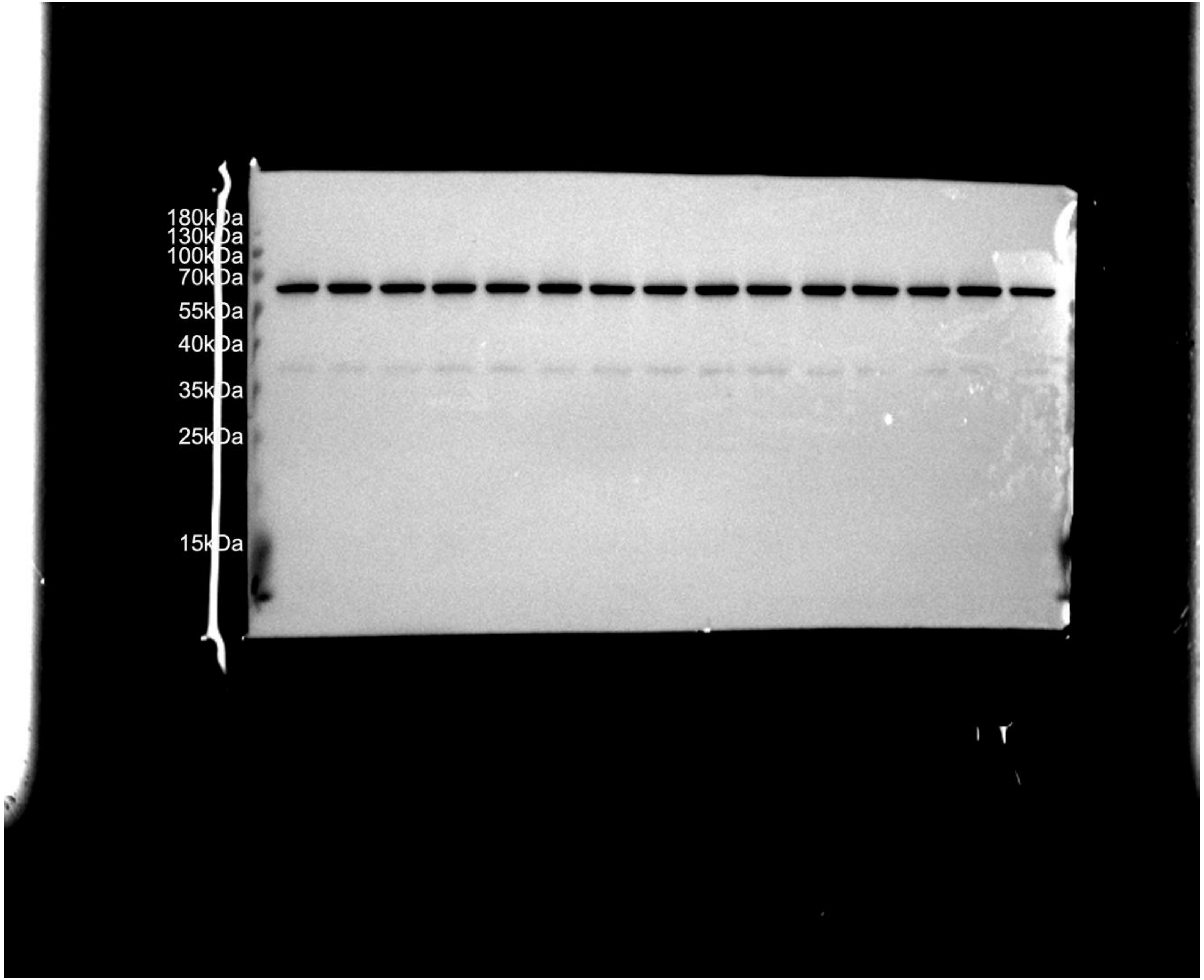

OPN

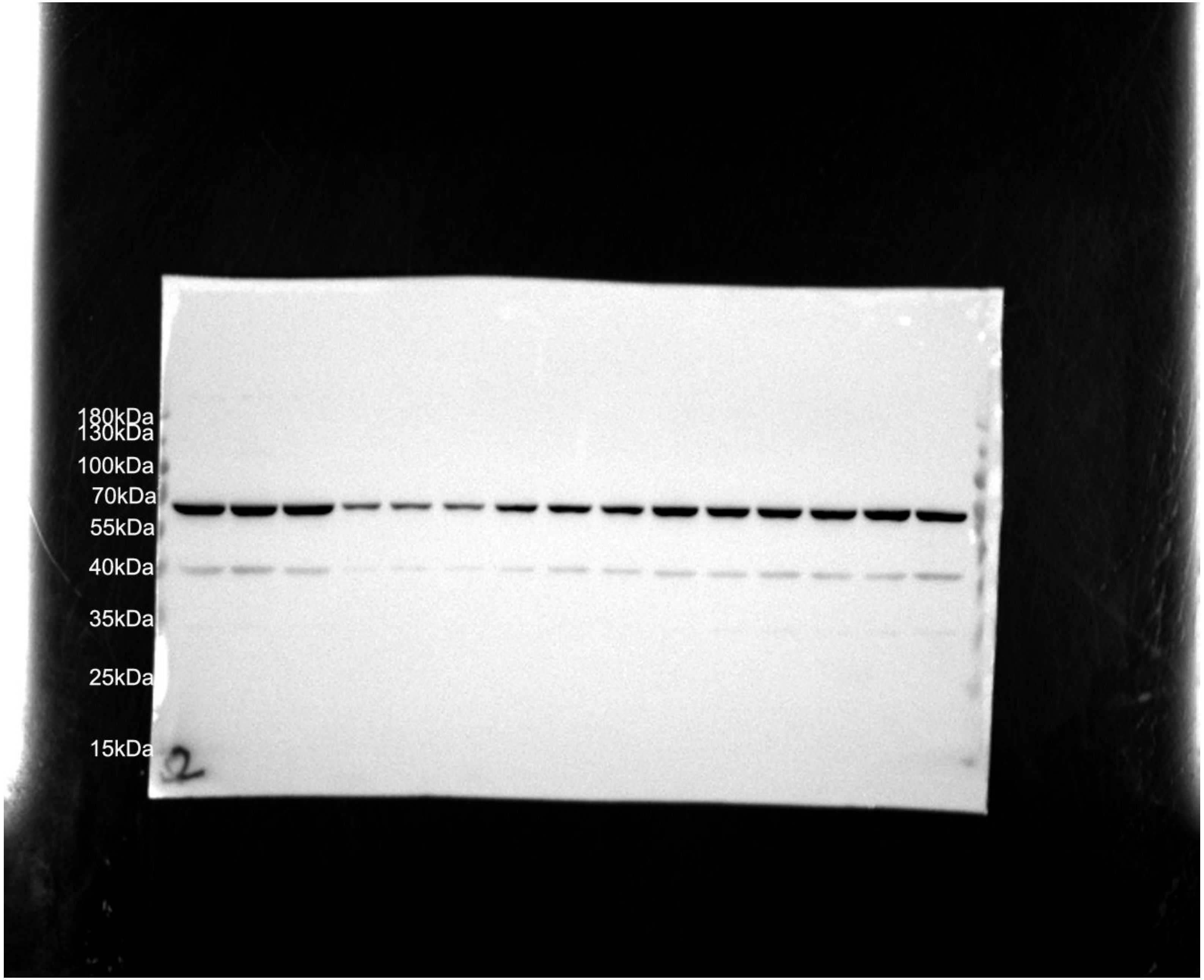

eNOS

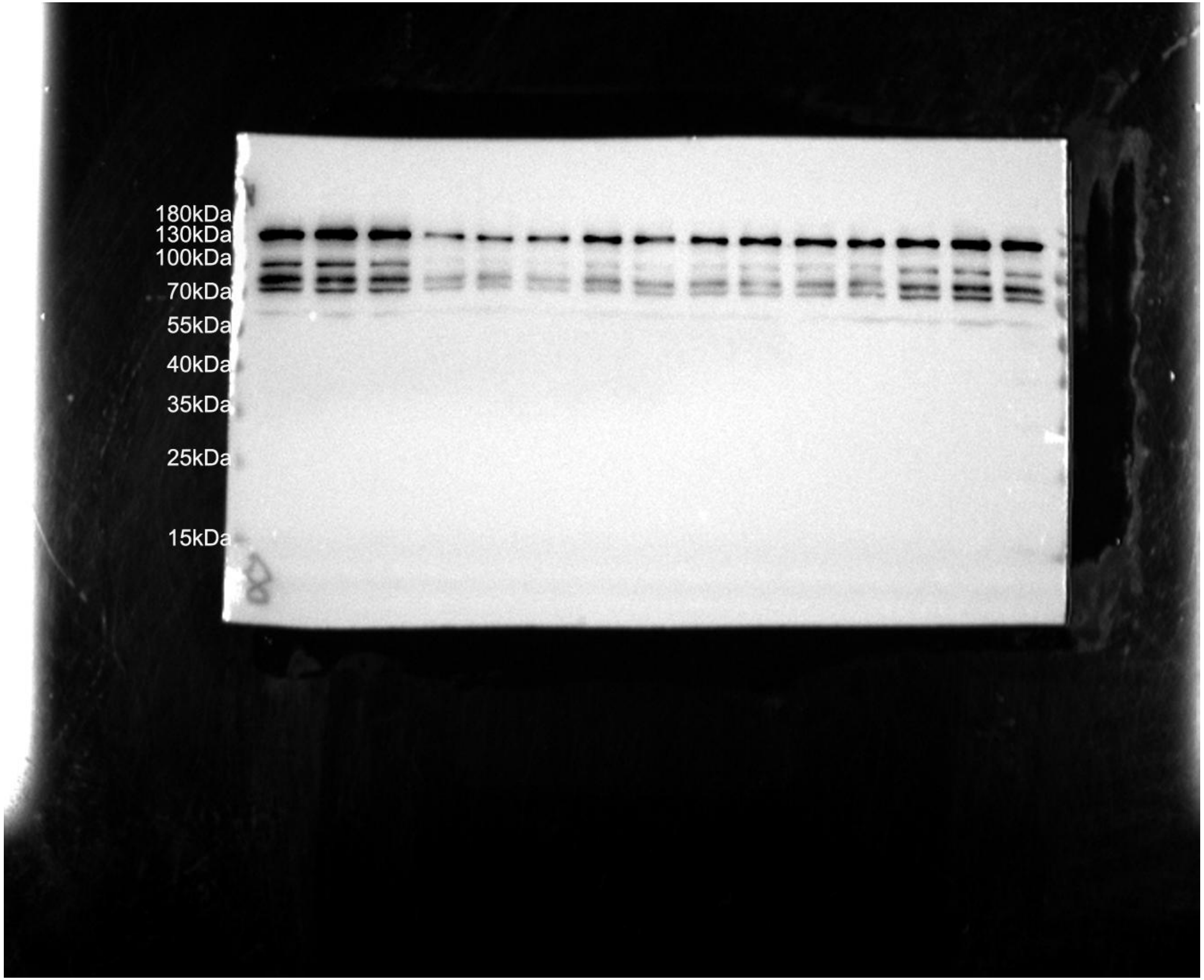

Bcl-XL

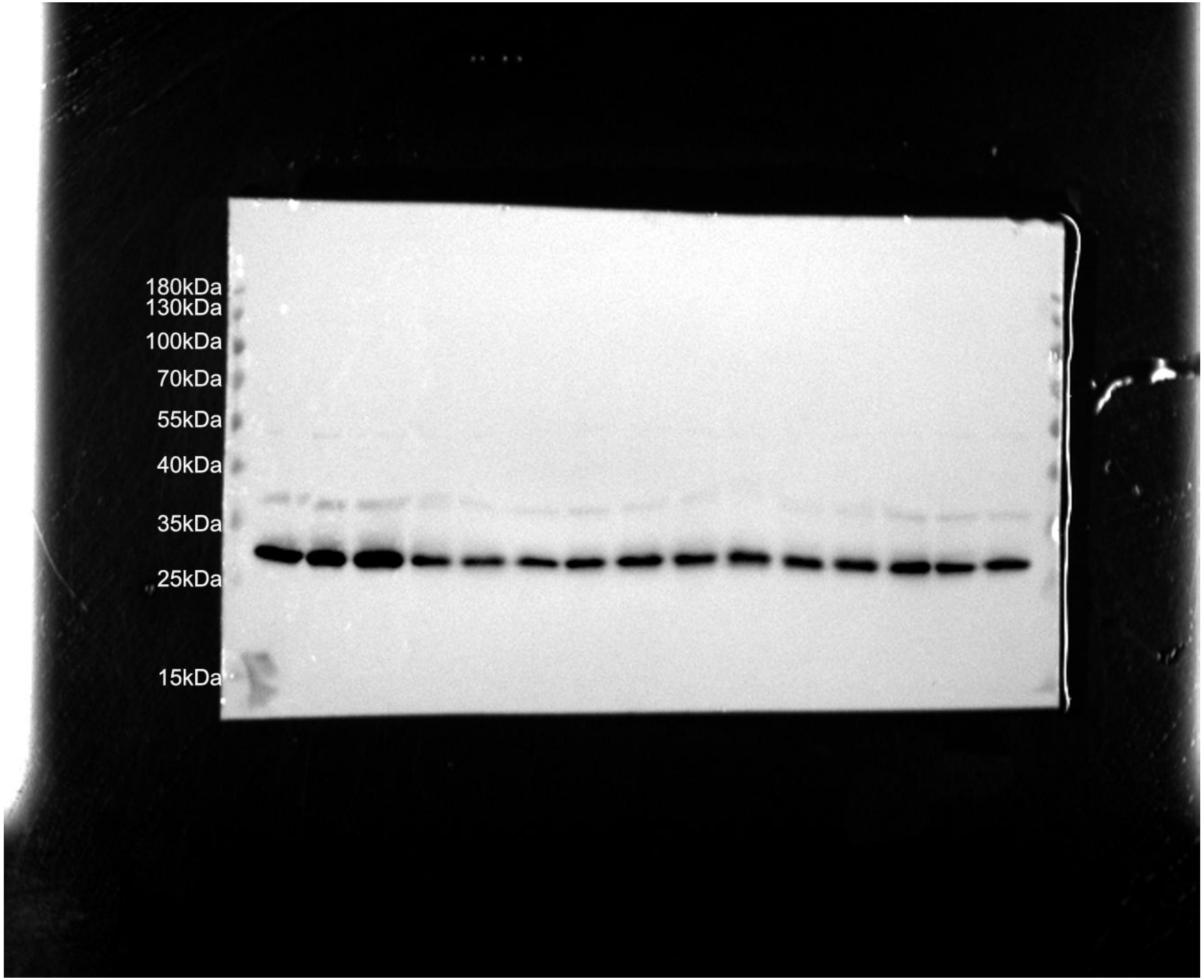

Bcl-2

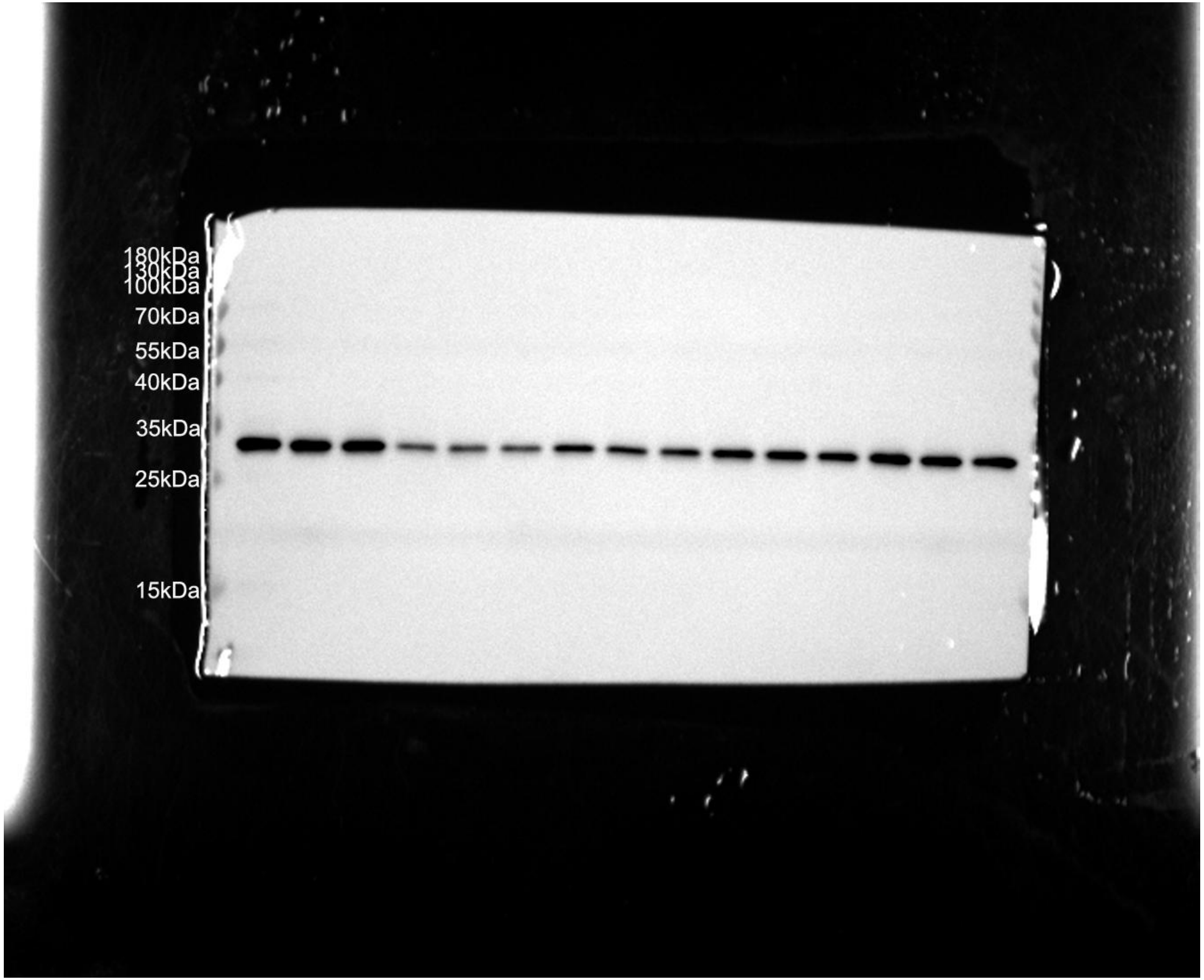

Myc

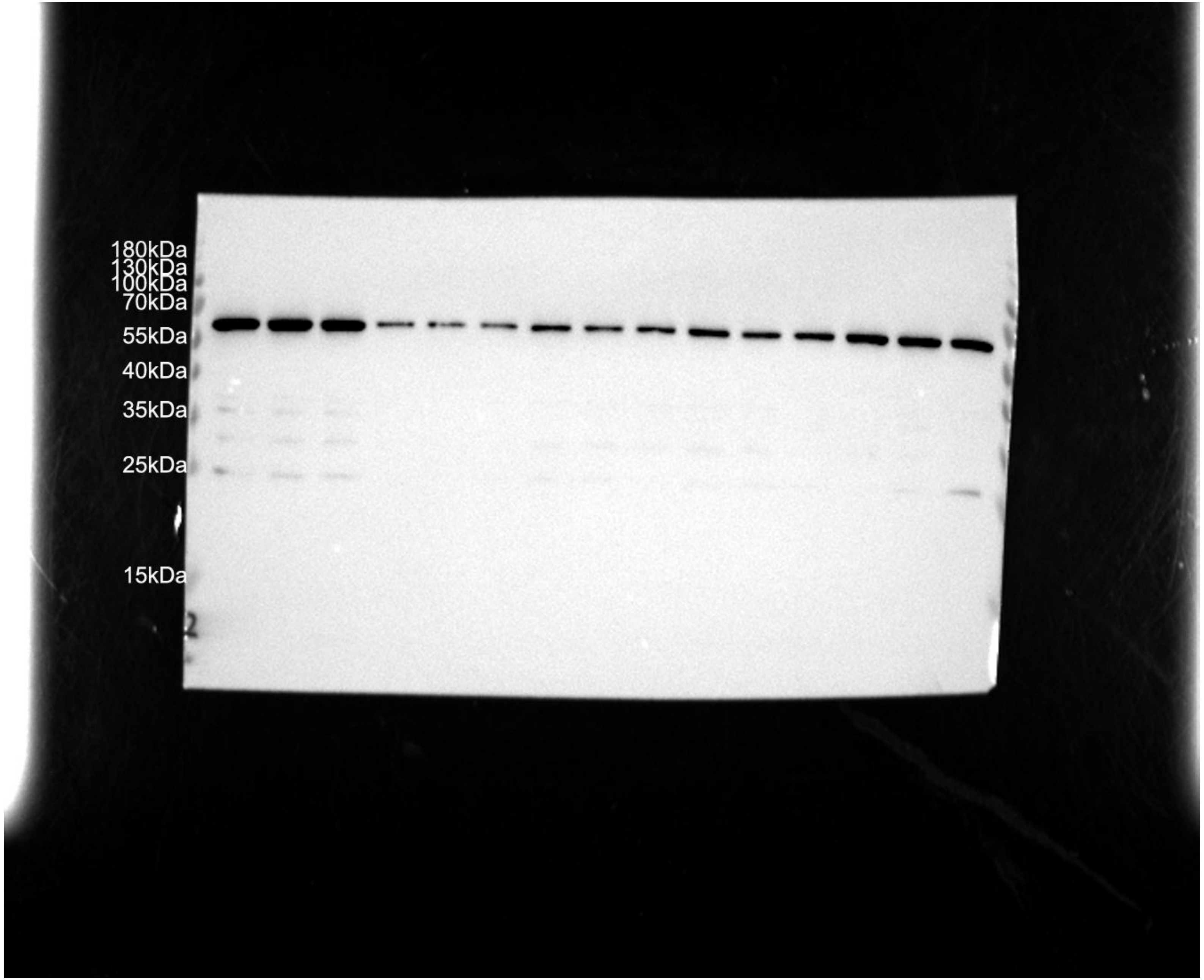

GAPDH

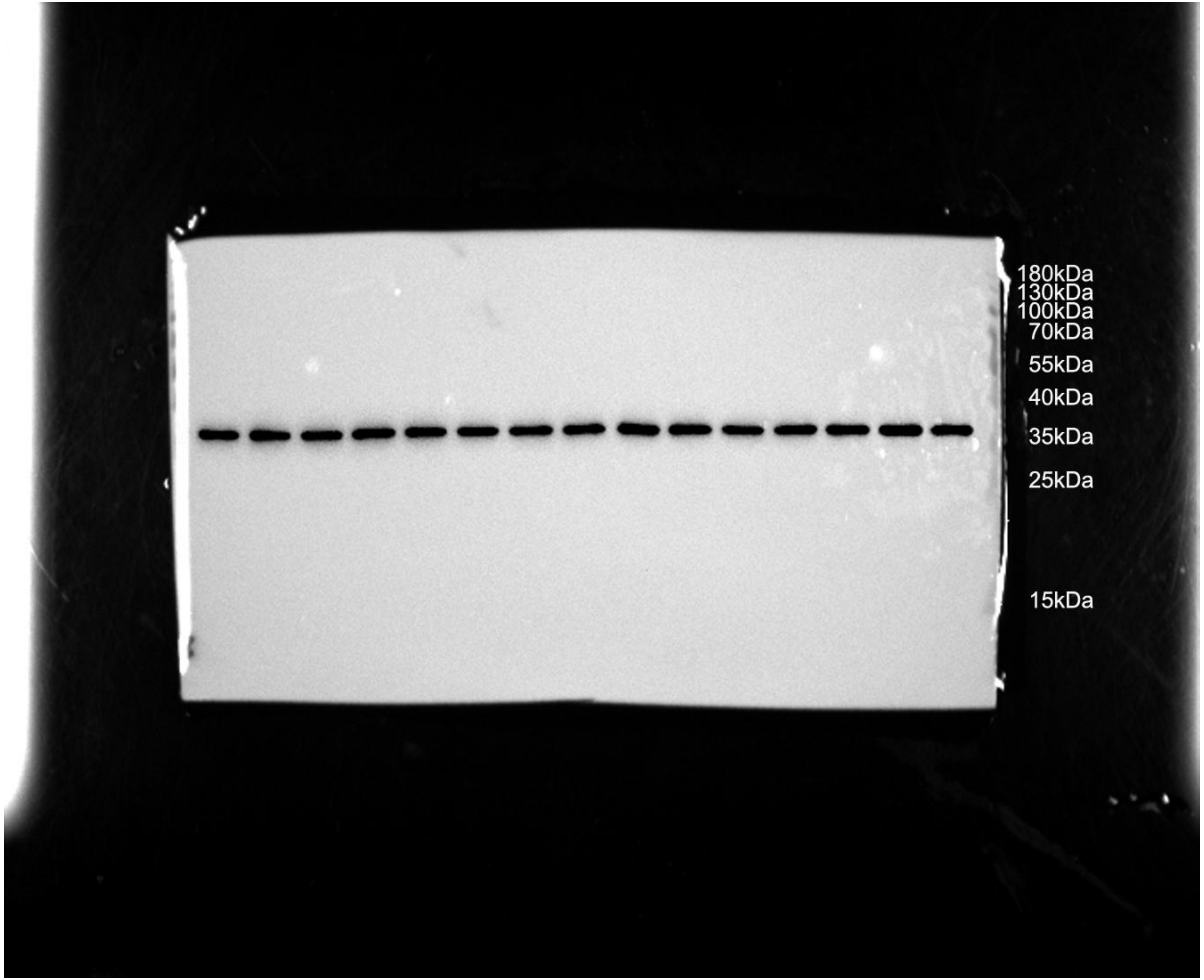

Supplement: Supplementary file 9 — Supplementary Material 9 [file 41065_2025_578_MOESM9_ESM.pdf]
