# Supplementary material for: Single-cell RNA sequencing reveals that Danggui Buxue Tang decoction facilitates wound healing after anal fistula by promoting M2 macrophage polarization
Source: Hereditas. 2025 Oct 9;162:204. doi: 10.1186/s41065-025-00578-2 (PMC12512633; doi:10.1186/s41065-025-00578-2)
Supplement: Supplementary file 4 — Supplementary Material 4: Supplementary Fig. 4. Ligand-receptor pairs analysis. (A). Ligand-receptor pairs between Macrophage_C1_Areg (M1-like (M1C1)) and other cell types. The vertical axis represents pairs of ligand and receptor genes, while the horizontal axis represents pairs of ligand-expressing cells and receptor-expressing cells. The color of the points represents the magnitude of the communication probability value, and the size of the points represents the magnitude of the P-value. (B). Ligand-receptor pairs between Macrophage_C3_Cd163 (M2-like (M2C3)) and other cell types. [file 41065_2025_578_MOESM4_ESM.pdf]

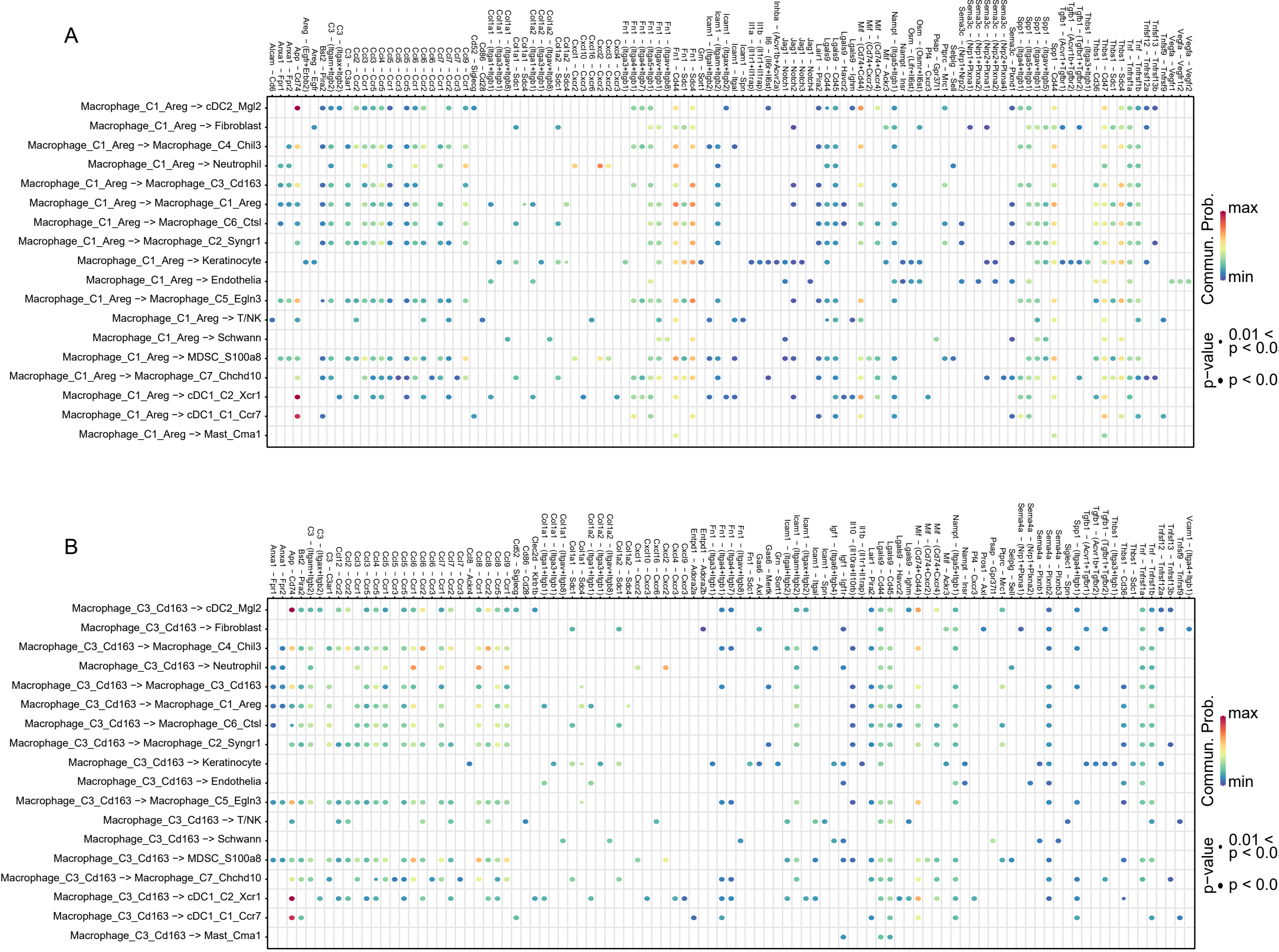
A
Macrophage_C1_Areg -> cDC2_Mgl2
Macrophage_C1_Areg -> Fibroblast
Macrophage_C1_Areg -> Macrophage_C4_Chil3
Macrophage_C1_Areg -> Neutrophil
Macrophage_C1_Areg -> Macrophage_C3_Cd163
Macrophage_C1_Areg -> Macrophage_C1_Areg
Macrophage_C1_Areg -> Macrophage_C6_Ctsl
Macrophage_C1_Areg -> Macrophage_C2_Syngr1
Macrophage_C1_Areg -> Keratinocyte
Macrophage_C1_Areg -> Endothelia
Macrophage_C1_Areg -> Macrophage_C5_Egln3
Macrophage_C1_Areg -> T/NK
Macrophage_C1_Areg -> Schwann
Macrophage_C1_Areg -> MDSC_S100a8
Macrophage_C1_Areg -> Macrophage_C7_Chchd10
Macrophage_C1_Areg -> cDC1_C2_Xcr1
Macrophage_C1_Areg -> cDC1_C1_Ccr7
Macrophage_C1_Areg -> Mast_Cma1
Commun. Prob.
max
min
p−value
0.01 < p < 0.0
p < 0.0
B
Macrophage_C3_Cd163 -> cDC2_Mgl2
Macrophage_C3_Cd163 -> Fibroblast
Macrophage_C3_Cd163 -> Macrophage_C4_Chil3
Macrophage_C3_Cd163 -> Neutrophil
Macrophage_C3_Cd163 -> Macrophage_C3_Cd163
Macrophage_C3_Cd163 -> Macrophage_C1_Areg
Macrophage_C3_Cd163 -> Macrophage_C6_Ctsl
Macrophage_C3_Cd163 -> Macrophage_C2_Syngr1
Macrophage_C3_Cd163 -> Keratinocyte
Macrophage_C3_Cd163 -> Endothelia
Macrophage_C3_Cd163 -> Macrophage_C5_Egln3
Macrophage_C3_Cd163 -> T/NK
Macrophage_C3_Cd163 -> Schwann
Macrophage_C3_Cd163 -> MDSC_S100a8
Macrophage_C3_Cd163 -> Macrophage_C7_Chchd10
Macrophage_C3_Cd163 -> cDC1_C2_Xcr1
Macrophage_C3_Cd163 -> cDC1_C1_Ccr7
Macrophage_C3_Cd163 -> Mast_Cma1
Commun. Prob.
max
min
p−value
0.01 < p < 0.0
p < 0.0
